# Polyvalent Nano-Lectin Potently Neutralizes SARS-CoV-2 by Targeting Glycans on the Viral Spike Protein

**DOI:** 10.1021/jacsau.3c00163

**Published:** 2023-06-12

**Authors:** Darshita Budhadev, James Hooper, Cheila Rocha, Inga Nehlmeier, Amy Madeleine Kempf, Markus Hoffmann, Nadine Krüger, Dejian Zhou, Stefan Pöhlmann, Yuan Guo

**Affiliations:** † School of Chemistry and Astbury Centre for Structural Molecular Biology, University of Leeds, Leeds LS2 9JT, United Kingdom; ‡ School of Food Science & Nutrition and Astbury Centre for Structural Molecular Biology, University of Leeds, Leeds LS2 9JT, United Kingdom; § Infection Biology Unit, German Primate Center − Leibniz Institute for Primate Research, 37077 Göttingen, Germany; ∥ Faculty of Biology and Psychology, Georg-August-University Göttingen, 37073 Göttingen, Germany

**Keywords:** antiviral, polyvalent nano-lectin, SARS-CoV-2, glycan, multivalency

## Abstract

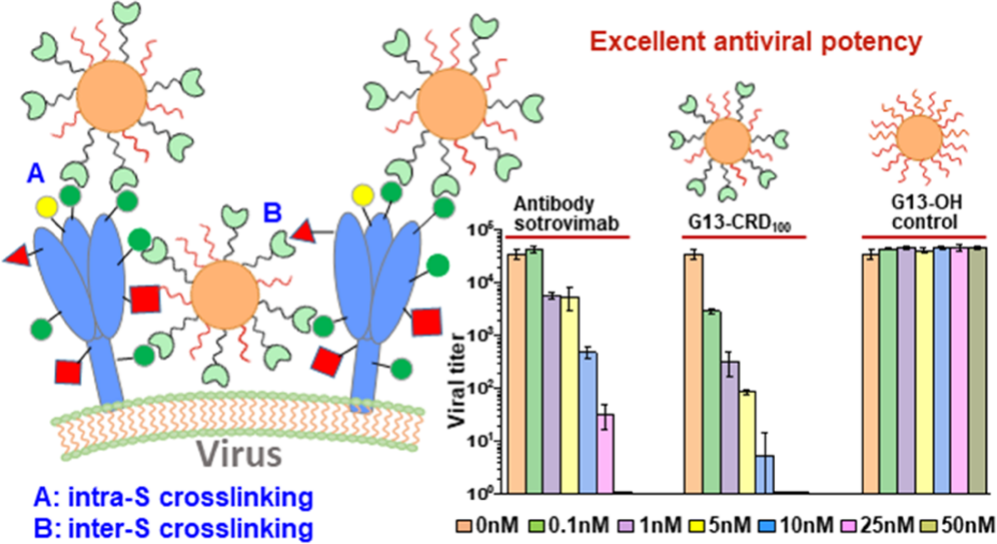

Mutations in spike (S) protein epitopes allow SARS-CoV-2 variants to evade antibody responses induced by infection and/or vaccination. In contrast, mutations in glycosylation sites across SARS-CoV-2 variants are very rare, making glycans a potential robust target for developing antivirals. However, this target has not been adequately exploited for SARS-CoV-2, mostly due to intrinsically weak monovalent protein–glycan interactions. We hypothesize that polyvalent nano-lectins with flexibly linked carbohydrate recognition domains (CRDs) can adjust their relative positions and bind multivalently to S protein glycans, potentially exerting potent antiviral activity. Herein, we displayed the CRDs of DC-SIGN, a dendritic cell lectin known to bind to diverse viruses, polyvalently onto 13 nm gold nanoparticles (named G13-CRD). G13-CRD bound strongly and specifically to target glycan-coated quantum dots with sub-nM *K*_d_. Moreover, G13-CRD neutralized particles pseudotyped with the S proteins of Wuhan Hu-1, B.1, Delta variant and Omicron subvariant BA.1 with low nM EC_50_. In contrast, natural tetrameric DC-SIGN and its G13 conjugate were ineffective. Further, G13-CRD potently inhibited authentic SARS-CoV-2 B.1 and BA.1, with <10 pM and <10 nM EC_50_, respectively. These results identify G13-CRD as the 1st polyvalent nano-lectin with broad activity against SARS-CoV-2 variants that merits further exploration as a novel approach to antiviral therapy.

## Introduction

The global Covid-19 pandemic caused by SARS-CoV-2 had a devastating impact on the healthcare systems and economies worldwide. The excess mortality associated with Covid-19 is believed to amount to 18 million from 2020 to 2021, and tens of millions are suffering from long-term physical and mental health problems (i.e., long Covid).^1^ To combat this, a number of antiviral strategies that target the viral infection process have been developed.^2−7^ As binding of the viral surface trimeric spike (S) protein to the ACE2 receptor on the host cell surface is essential for infectious SARS-CoV-2 entry into cells,^8,9^ vaccines (including those under development) and several antivirals target this interaction.^2,4,6,7^ For example, neutralizing antibodies (Abs) bind to the S protein and block infectious viral entry into cells.^4,7^ However, the emergence of SARS-CoV-2 variants with mutations in the S protein that alter antibody epitopes can allow for evasion of neutralizing Abs induced upon vaccination and/or infection.^10−14^

The SARS-CoV-2 S protein trimer is heavily glycosylated with 22 N-linked glycans on each monomer subunit, consisting of oligomannose, hybrid, and complex glycans.^15,16^ Glycosylation plays a critical role in viral pathobiology, which include mediating S protein folding and stability, camouflaging immunogenic epitopes, and facilitating ACE2 binding and viral cell entry.^17^ Unlike the frequently changing S protein epitopes targeted by neutralizing Abs, all N-glycosylation sites are conserved in SARS-CoV-2 variants of concern identified by the World Health Organization (WHO) except for the γ (having 2 extra sites at N20 and N188),^18^ and Delta and Omicron BA.2-5 variants (loss of N17 site, due to disruption of sequon from T19 mutation, see Table S1). While viral glycans are synthesized by the host cell machinery, they exhibit some unique features that differentiate them from host self-glycans, e.g., a high content of underprocessed oligomannoses and high glycan density. These make viral surface glycans an attractive target for developing antivirals. Indeed, a few rare but potent and broadly neutralizing Abs target glycans on HIV.^19−21^ For example, antibody 2G12 displays an unusual domain-exchanged structure that brings two Fabs in close proximity to create an extended glycan binding surface, allowing 2G12 to form strong multivalent interactions with densely packed glycans on the same gp160 trimer on the HIV surface.^22^ This binding is not possible with conventional Abs: their Fabs are too widely (∼15 nm) spaced to allow for simultaneous binding to the same gp160 molecule. Unfortunately, due to low natural immunogenicity, glycan-targeting Abs remain rare and to date, no anti-SARS-CoV-2 neutralizing Abs are known to be glycan-targeting. Nevertheless, the success of 2G12 and a few other Abs clearly demonstrates that targeting viral glycans by exploiting multivalency is a viable antiviral strategy.

**Figure 1:**
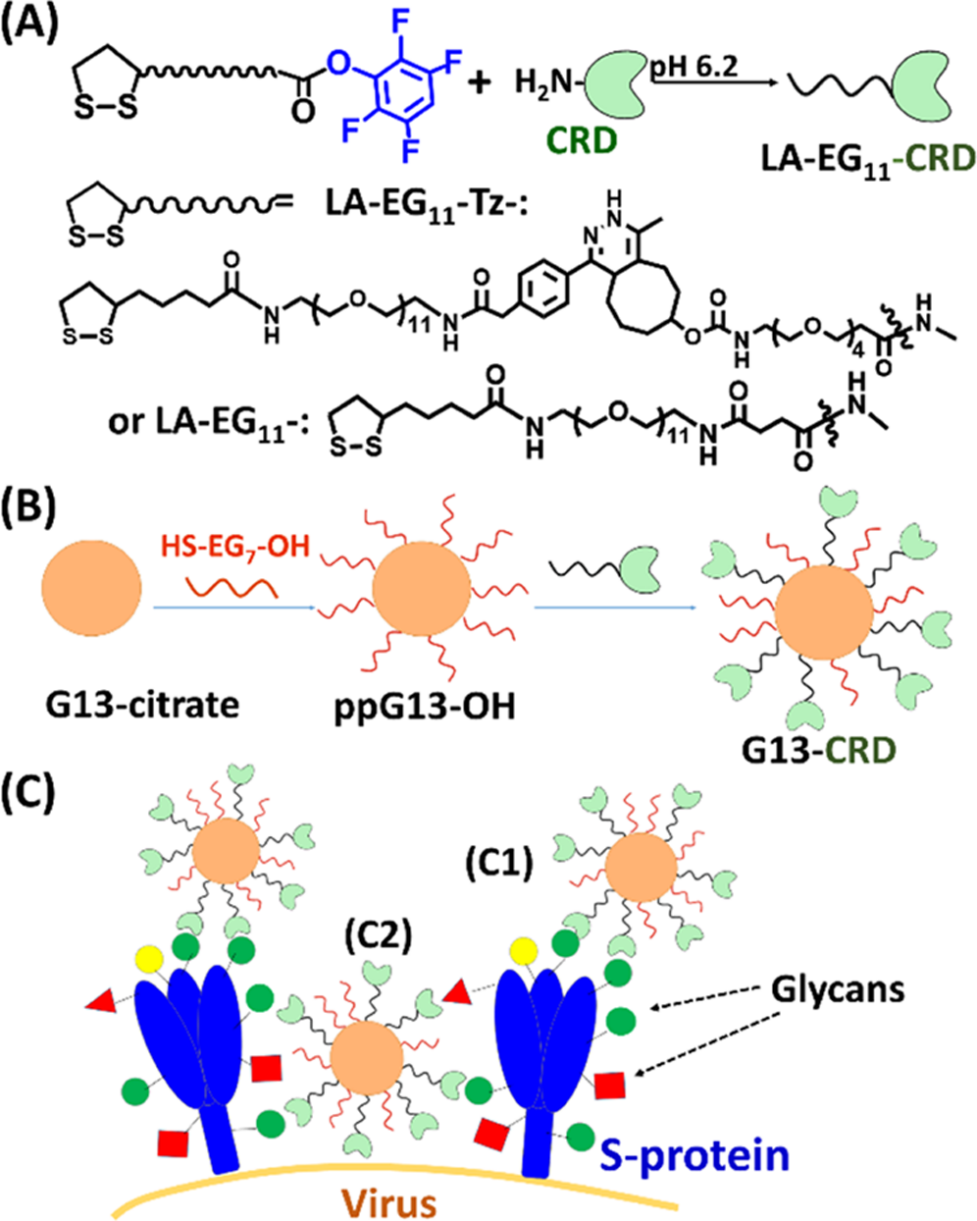
(A, B) Schematic route to prepare an N-terminal LA-EG_11_-linker-labeled lectin (A) and a polyvalent nano-lectin (B) exemplified using DC-SIGN CRD as a model lectin. Lectin N-terminal amine is selectively labeled with an LA-EG_11_-linker at pH 6.2. A citrate-stabilized 13 nm gold nanoparticle (G13) is first partially PEGylated with HS-EG_7_-OH, and then conjugated with LA-EG_11_-linker-labeled CRDs to form G13-CRD via self-assembly. (C) Schematic representation of possible interactions between G13-CRD and S protein glycans on the viral surface: (C1) steric blockade of binding of the receptor binding domain in the S protein to the host cell receptor ACE2; and (C2) crosslinking two S proteins on the virion surface to interrupt S protein conformational changes required for infectious entry.

Multivalent lectin–glycan interactions are widespread and highly effective in enhancing binding affinity and specificity.^23−25^ They also play a key role in pathogen recognition and immune regulation. It is therefore unsurprising that lectins can display useful antiviral activities by binding to viral surface glycans to block cell entry.^26,27^ However, some lectins, including a dendritic cell surface tetrameric lectin, DC-SIGN, have been shown to bind and transmit SARS-CoV-2 to target cells, albeit less effective than ACE2.^28−30^ Therefore, we propose a new polyvalent nano-lectin antiviral strategy by displaying DC-SIGN tetrameric extracellular domain (ECD) or its monomeric carbohydrate recognition domain (CRD) polyvalently and flexibly on gold nanoparticle (GNP) scaffolds. We hypothesize that the flexibly displayed ECDs or CRDs in each nano-lectin will be able to adjust their relative positions, allowing for strong multivalent binding to glycans on viral S proteins to inhibit viral entry (see Figure 1). Moreover, polyvalent nano-lectins may bind to glycans from different domains on the same trimeric S protein (i.e., intra-spike crosslinking) and/or in between neighboring S proteins on virion particles (inter-spike crosslinking). Such binding may interrupt S protein conformational changes that are essential for virus entry into cells.^31,32^ A GNP scaffold is chosen here because of excellent biocompatibility, low-/non-cytotoxicity, tunable size, and robust gold-thiol chemistry for convenient surface modification and bioconjugation.^33,34^ Hence, the key parameters (e.g., size, lectin valency and flexibility) required for potent virus neutralization can be readily tuned. In addition, GNP glycan or peptide conjugates have been successfully used to detect SARS-CoV-2 virus or antiviral IgG Abs, respectively.^35,36^

## Results

### DC-SIGN-GNP Conjugation and Specific Glycan Binding

The ECD has been shown to form a stable tetramer and retain the glycan binding properties of native DC-SIGN,^37^ while the monomeric CRD defines glycan binding specificity.^38^ DC-SIGN CRD binds specifically to mannose- and fucose-containing glycans found on virus surfaces, including SARS-CoV-2, with low to moderate monovalent affinities (*K*_d_’s: 0.1–3 mM), while the tetrameric ECD (containing 4 CRDs) displays augmented binding affinity.^39^ Both DC-SIGN ECD and CRD were recombinantly expressed in *Escherichia coli* and purified by mannose affinity columns as confirmed by high-resolution mass spectrometry (HRMS, Supporting Information, Section 2), as reported previously.^40,41^ To facilitate lectin-GNP conjugation, two linker molecules based upon a general structure of lipoic acid-undecyl(ethylene glycol)-carboxylic acid tetrafluorobenze ester (LA-EG_11_-TFP) were designed. Both linkers contain three functional domains: an LA group for strong GNP anchoring by forming two strong Au–S bonds: an EG_11_ spacer for good flexibility, water solubility, and resisting nonspecific interactions,^42,43^ and a TPF ester for protein labeling via reacting to a free surface amine (Figure 1). We first prepared linker **1**, LA-EG_11_-Tz-TFP, by reacting LA-EG_11_-tetrazine with trans-cyclooctyne-EG_4_-TFP ester (TCO-EG_4_-TFP) via the copper-free click reaction between tetrazine and TCO (Supporting Information, Section 3.1). While this reaction was rapid, the LA-EG_11_-Tz-TFP linker was unstable for long-term storage, even at −20 °C, and gradually degraded over 4 months. We therefore prepared linker **2** (LA-EG_11_-TFP) by direct esterification of LA-EG_11_-CO_2_H with TFP (Supporting Information, Section 3.2). Linker 2 was highly stable, showing minimal degradation after storage for 12 months at −20 °C as a lyophilized powder.

To ensure all CRDs conjugated on the GNP surface are oriented and available for binding, the N-terminal amine in DC-SIGN ECD or CRD was selected for linker labeling. The pKa of N-terminal α-amine is >2 pH units lower than protein surface ε-amines of lysine residues (e.g., ∼6.0 to 8.0 vs ∼10.5).^44^ Thus, labeling was conducted at pH 6.2, ensuring that only the α-amine, but not ε-amines, was nonprotonated and available to react with TFP ester to form a stable amide linkage. Incubating the protein with LA-EG_11_-Tz-TFP at a 1:1.5 molar ratio for ∼40 min was sufficient to produce single-linker-labeled proteins in ∼18 and ∼22% yields for ECD (denoted as LA-EG_11_-Tz-ECD) and CRD (denoted as LA-EG_11_-Tz-CRD), respectively. Extending the incubation time led to the formation of dual-labeled proteins. The same condition was used to label linker LA-EG_11_-TFP, giving single-linker-labeled CRD (denoted as LA-EG_11_-CRD) in ∼19% yield and a very small amount (∼2%) of dual-labeled CRD (denoted as (LA-EG_11_)_2_-CRD) (Supporting Information, Section 4).

A 13 nm GNP (G13) was synthesized by citrate reduction of H[AuCl_4_] (Supporting Information, Section 5)^45^ and used to construct antiviral nano-lectins in two steps. First, G13 was partially PEGylated with a hepta(ethylene glycol) thiol (HS-EG_7_-OH) to prevent aggregation during lectin conjugation. This was achieved by overnight incubation of G13 with 2000 molar equivalents of HS-EG_7_-OH in water to yield ppG13-OH. Second, ppG13-OH was incubated with 100 molar equivalents of linker-labeled lectins overnight to make G13-lectin-based polyvalent nano-lectins via self-assembly (Figure 1 and Supporting Information, Section 5.2.2). No linker-labeled lectins were found in any of the post conjugation supernatants from HRMS analysis. Therefore, all linker-labeled lectins added must have conjugated to G13, giving a lectin valency of 100 for LA-EG_11_-Tz-ECD, LA-EG_11_-Tz-CRD, or LA-EG_11_-CRD per G13, abbreviated as G13-Tz-ECD_100_, G13-Tz-CRD_100_ or G13-CRD_100_, respectively. To investigate the effect of CRD valency on antiviral activity, another batch of G13-CRD was prepared at an LA-EG_11_-CRD: ppG13-OH ratio of 115:1 (denoted as G13-CRD_115_).

**Figure 2:**
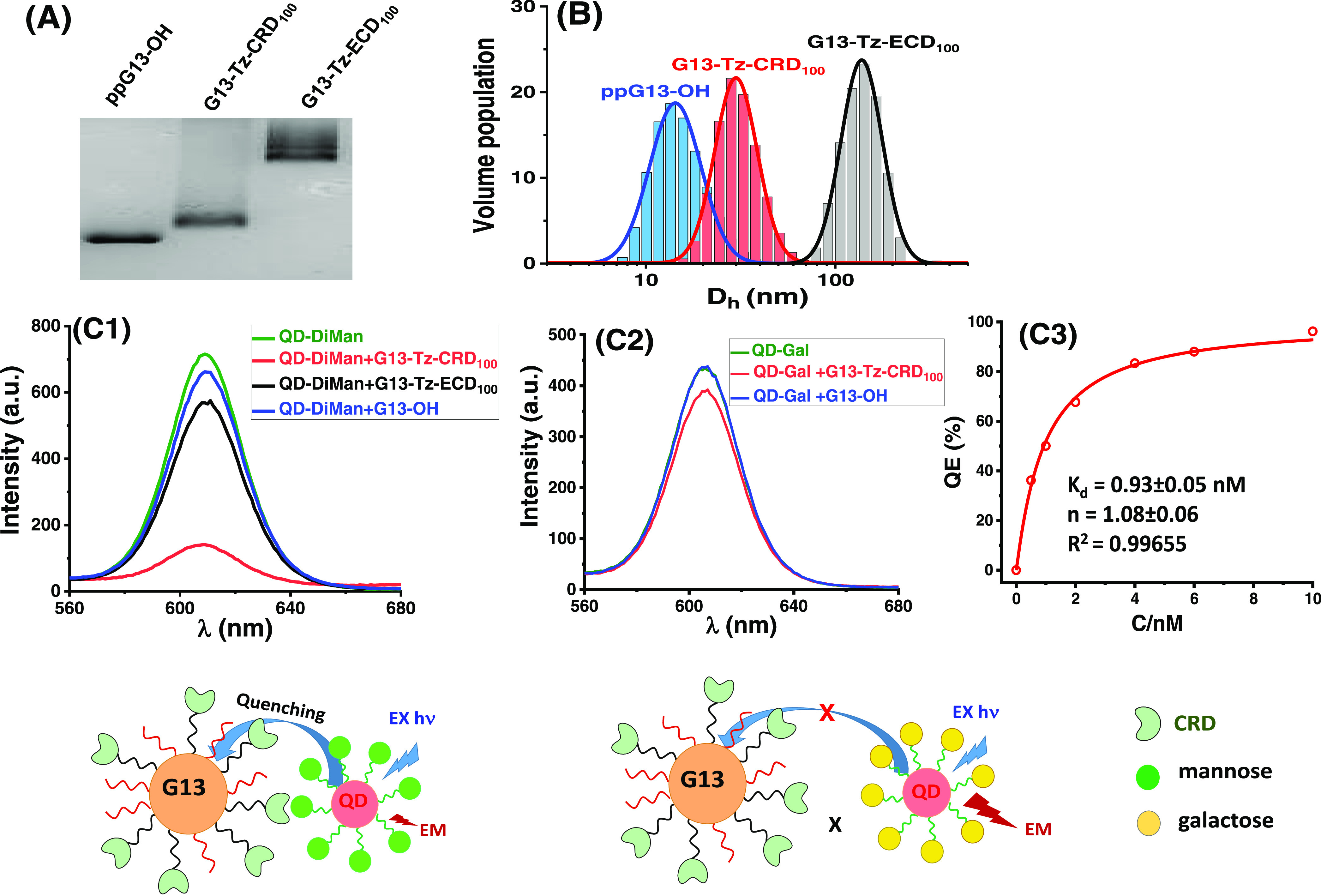
(A) 1.5% Agarose gel electrophoresis reveals ppG13-OH has the highest, followed by G13-Tz-CRD_100_, while G13-Tz-ECD_100_ shows the lowest gel mobility. The bands are visible under room light without staining. (B) Hydrodynamic diameter (*D*_h_) histograms fitted by log-normal Gaussian function, yielding a *D*_h_ of ∼17, ∼31, and ∼144 nm for ppG13-OH, G13-Tz-CRD_100_, and G13-Tz-ECD_100_, respectively. (C1) QD-DiMan (2 nM) fluorescence is efficiently quenched by 1 nM G13-Tz-CRD_100_ (>80%), but much less so by 1 nM G13-Tz-ECD_100_ (∼20%), and only marginally by 1 nM G13-OH control (∼7.5%); (C2) QD-Gal (2 nM) control is marginally quenched by G13-Tz-CRD_100_ (1 nM, ∼9.5%), but not by G13-OH control. (C3) Quenching efficiency (QE)–concentration (*C*) relationship of 1:1 molar mixed G13-Tz-CRD_100_ and QD-DiMan fitted by Hill’s equation (QE_max_ fixed to 100), giving an apparent binding *K*_d_ of 0.93 ± 0.05 nM.

The success of G13-lectin conjugation was supported by the reduced gel mobility over ppG13-OH (Figure 2 and Supporting Information, Section 5.3.1), and increased hydrodynamic diameters (*D*_h_s) following each conjugation step. For example, *D*_h_ was increased from *∼*15 nm (G13-citrate) to ∼17 nm (ppG13-OH), and then to ∼31 nm for G13-Tz-CRD_100_ or ∼140 nm for G13-Tz-ECD_100_ (Figure 2) or ∼22 nm for G13-CRD_100_ and ∼26 nm for G13-CRD_115_ (Supporting Information, Section 5.3.2).

To confirm that polyvalent nano-lectins retained CRD’s native glycan binding specificity, we also prepared a CdSe/ZnS quantum dot (QD, λ_em_ ∼ 600 nm) coated with a DHLA-EG_4_-mannose-α-1,2-mannose^39,40^ (DiMan, a DC-SIGN CRD binding glycan, Supporting Information, Section 3.3) or a DHLA-EG_4_-galactose (Gal, a DC-SIGN CRD nonbinding glycan) ligand (denoted as QD-DiMan or QD-Gal) as a positive- or a negative- control, respectively (Figure 2 and Supporting Information, Sections 3.3 and 3.4). These are based on our earlier findings that QD-DiMan binds strongly to tetrameric DC-SIGN ECD (low nM *K*_d_’s), but shows no measurable binding to monomeric CRD (due to CRD-DiMan monovalent binding (*K*_d_ = 0.9 mM), being too weak to measure with 20 nM QD).^39^ Moreover, the tetrameric DC-SIGN ECD showed no apparent binding with Gal in a glycan microarray format.^46^

GNP is well known for its strong fluorescence quenching properties via a nanoscale surface energy transfer mechanism (QE ∝ 1/*R*^4^, where QE and *R* are the quenching efficiency and dye-GNP distance, respectively), which is more effective and covers a longer distance range than the Förster resonance energy transfer (QE ∝ 1/*R*^6^).^47,48^ Therefore, binding of G13-CRD with DiMan-QD will bring the QD and GNP into close proximity, resulting in efficient quenching of QD fluorescence. The QE here represents the percentage of added QDs that have bound to G13-CRD (Supporting Information, Section 5.3.3).^40^ As expected, the fluorescence of QD-DiMan (2 nM) was quenched efficiently (>80%) upon mixing with G13-Tz-CRD_100_ (1 nM, Figure 2C1). In contrast, QD-Gal was quenched much less efficiently (∼10%, Figure 2C2), and a nonglycosylated QD-EG_4_-OH control (a DHLA-EG_4_-OH ligand capped QD) showed no apparent quenching under such conditions. Fitting the QE-concentration relationship for 1:1 mixed G13-Tz-CRD_100_ and QD-DiMan by Hill’s equation yielded an apparent binding *K*_d_ of 0.93 ± 0.05 nM (Figure 2C3).^39,40^ This represents an impressive ∼1 million-fold enhancement of affinity over that of monovalent CRD-DiMan binding (*K*_d_ = 0.9 mM).^39^ A similar sub-nM *K*_d_ was also obtained for G13-CRD_100_ binding with QD-DiMan (Figure S5.4). These results confirm that G13-CRDs not only retained CRD’s native binding specificity with DiMan but also drastically enhanced the affinity via multivalent binding (Figure 2C). We have found previously that free tetrameric ECD (before GNP conjugation) binds strongly with QD-DiMan with low- to sub-nM *K*_d_.^39^ However, G13-Tz-ECD_100_ only gave a QE about ∼1/4 of that obtained with G13-Tz-CRD_100_ after QD-DiMan binding (Figure 2C1). The ineffective quenching here is attributed to the long rigid coiled-coil neck (>20 nm) in ECD which projects CRDs away from the GNP surface, resulting in a large GNP-QD separation distance and hence ineffective quenching.

### GNP-CRD Inhibition of SARS-CoV-2 Pseudotypes Entry into Vero76 Cells

**Figure 3:**
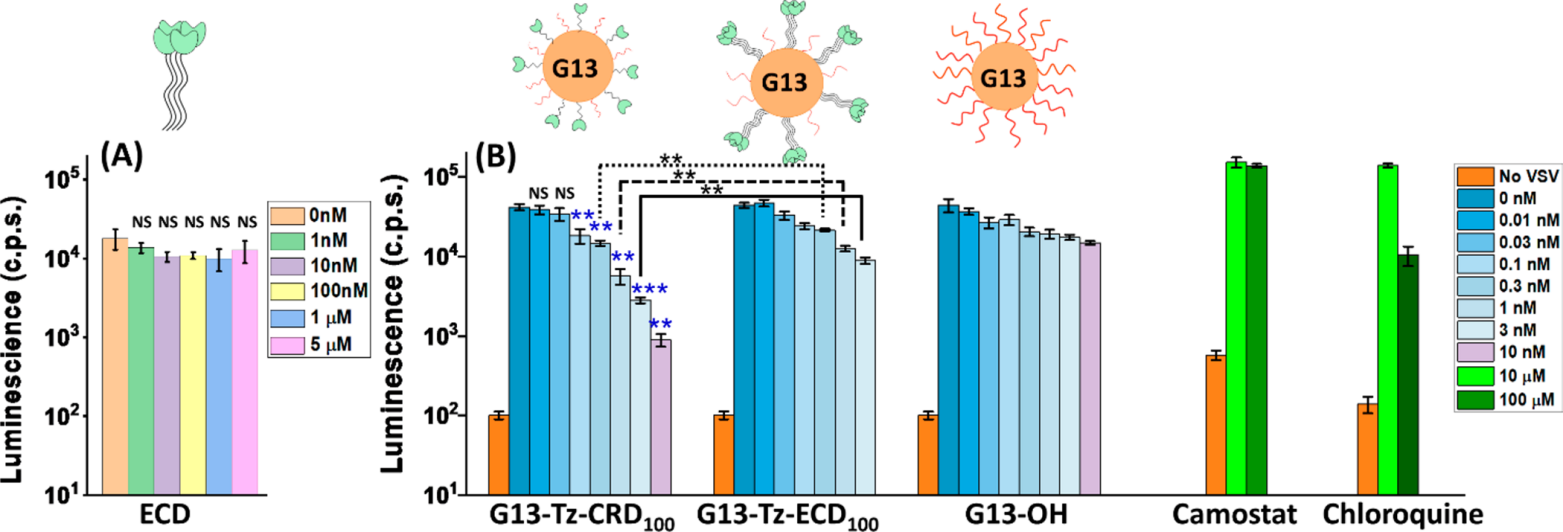
Dose-dependent inhibition of SARS-CoV-2 S protein-driven entry into Vero76 cells. VSV particles bearing SARS-CoV-2 S protein (Wuhan wild-type, Hu-1) were pre-incubated with (A) tetrameric DC-SIGN ECD or (B) G13-Tz-CRD_100_, G13-Tz-ECD_100_, or G13-OH before addition to target cells. As control, target cells were pre-incubated with Camostat or Chloroquine before addition of pseudotype particles. Entry efficiency was determined by quantifying luciferase activity in cell lysates. The orange bars in (B) represent the background luminescence measured in the absence of viral particles. The results of a representative experiment performed with technical quadruplicates are shown and were confirmed in two separate experiments. Errors bars indicate standard errors. No significant differences (*p* > 0.05) were observed for cells treated with viral particles without and with ECD (A). No significant differences (*p* > 0.05) between G13-Tz-CRD_100_ and G13-Tz-ECD_100_ were observed at doses of ≤0.1 nM, but significant differences (*p* < 0.01) were measured at doses of ≥0.3 nM. No significant differences (*p* > 0.05) were observed between G13-Tz-CRD_100_ and G13-OH at doses of ≤0.3 nM, but significant differences were observed at high doses, e.g., 1 nM (*p* < 0.01); 3 nM (*p* < 0.01) and 10 nM (*p* < 0.001). All statistical analysis was performed with a Brown–Forsythe and Welch ANOVA analysis with Dunnett’s T3 multiple comparison test: NS (not significant) *p* > 0.05; *p ≤ 0.05; **p ≤ 0.01; ***p ≤ 0.001.

Replication-defective single-cycle Vesicular Stomatitis Virus (VSV) reporter particles encoding luciferase and bearing the S protein of SARS-CoV-2 were employed to evaluate polyvalent nano-lectins’ inhibitory effect against SARS-CoV-2 S protein-driven entry into Vero76 cells (Supporting Information, Section 6). Previously, we and others have shown that these particles adequately model SARS-CoV-2 entry into cells and its inhibition.^9,14,49^ All inhibition studies were performed in Dulbecco’s modified Eagle medium (DMEM) supplemented with 10% fetal bovine serum (FBS) and penicillin (100 U/mL)/streptomycin (0.1 mg/mL) solution (P/S). The natural tetrameric ECD did not inhibit entry even at high doses of 5 μM (no statistically significant differences were observed, Figure 3A). In contrast, both G13-Tz-CRD_100_ and G13-Tz-ECD_100_ dose-dependently inhibited SARS-CoV-2 S protein-driven cell entry and inhibition by G13-Tz-CRD_100_ was found to be more potent than that by G13-Tz-ECD_100_ at higher doses (Figure 3B). However, neither of them showed significant inhibition against entry driven by the control VSV glycoprotein (VSV-G; Figure S6.1), indicating that inhibition of SARS-CoV-2 S protein-driven entry was specific.

The dose-dependent inhibition data were fitted by a modified inhibition model (eq 1)
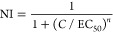
1where NI, *C*, EC_50_, and *n* are normalized infection, inhibitor concentration, effective inhibitor concentration giving 50% inhibition, and inhibition coefficient (with *n* >1, = 1 and <1 indicating positive-, none-, and negative-inhibition cooperativity),^25,40^ respectively. While the EC_50_ value is clearly important for viral inhibition, the inhibiting *n* value is also of great importance. For example, if three inhibitors have the same EC_50_ value but different *n* values of 0.5, 1, and 2, then the *C* required for 99% inhibition will be 9801, 99, and 9.9 times the EC_50_ value, respectively. The inhibitor with *n* = 0.5 is much less effective than that with *n* = 1 or 2, and requires ∼100- or ∼1000-fold higher dose in order to achieve the same 99% inhibition, despite having the same EC_50_ value. Therefore, viable inhibitors should have *n* ≥ 1 (with *n* = 1 being the most widely observed) in order to achieve complete inhibition with a reasonable *C*. However, inhibitors with *n* < 1 are unlikely to become viable inhibitors because of the difficulty to achieve complete inhibition.

The fit gave comparably low sub-nM EC_50_ values for both G13 -Tz-ECD_100_ and G13-Tz-CRD_100_, e.g., 0.25 ± 0.04 nM vs 0.19 ± 0.02 nM (see Figure S6.2), indicating a high antiviral potency. However, the inhibition profile of G13-Tz-ECD_100_ gave *n* = 0.57 ± 0.06, meaning it is difficult to achieve complete inhibition. In contrast, the inhibition profile of G13-Tz-CRD_100_ yielded *n* = 1, meaning it can achieve complete viral inhibition by increasing *C*. This is evident from that, despite having similar sub-nM EC_50_ values, the normalized infection for G13-Tz-ECD_100_ is >3-fold that for G13-Tz-CRD_100_ at 3 nM (Figure S6.2). This is further backed up by statistical analysis: their inhibition data are significantly different statistically at doses of ≥ 0.3 nM (*p* < 0.01, see Figure 3B). Therefore, presenting monomeric CRDs flexibly in a polyvalent nano-lectin, with each CRD serving as an independent binder, is key to potent viral inhibition. This is presumably because such flexible CRD binding units can readily adjust their relative positions to accommodate viral surface glycans and form strong multivalent binding. In contrast, the minimal independent binding unit in G13-Tz-ECD_100_ is a tetrameric ECD containing 4 CRDs. The CRD positions are fixed in each ECD unit and cannot readily adjust their relative positions to adapt to viral surface glycans, making it difficult to form strong simultaneous multivalent binding. In fact, most natural multimeric lectins are known to display fixed CRD presentations, allowing them to recognize specific, spatially matched multivalent glycans. As a result, their CRDs often lack the flexibility and adaptability required to achieve complete viral inhibition, making them ineffective as antiviral reagents. The G13-OH control showed no significant inhibition as expected (Figure 3B), demonstrating that viral inhibition was due to specific lectin–glycan interactions. Camostat (an inhibitor of the SARS-CoV-2 S protein activating protease TMPRSS2)^8,9^ exhibited no inhibitory effect even at 100 μM, as expected, since Vero cells do not express TMPRSS2. In contrast, chloroquine displayed significant inhibition at ∼100 μM, as expected (Figure 3B).

**Figure 4:**
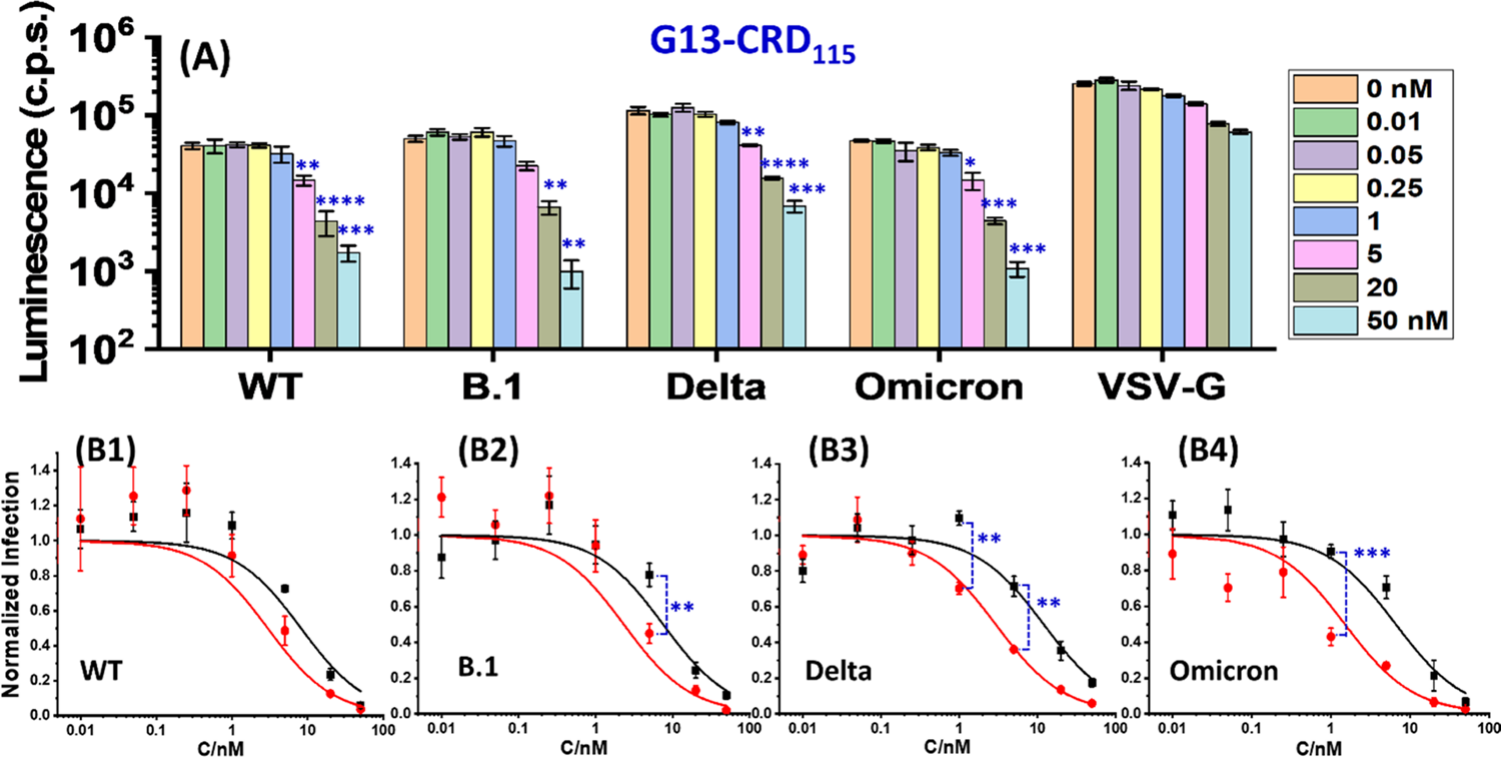
Dose-dependent inhibition of cell entry driven by the S proteins of SARS-CoV-2 variants. VSV particles bearing the indicated SARS-CoV-2 S proteins (Wuhan Hu-1 (WT), B.1, Delta (B.1.617.2), Omicron (BA.1)) were pre-incubated with G13-CRD_115_ (A) at the indicated concentrations in DMEM containing 10% FBS, and then added to Vero76 cells. Entry into Vero76 cells was determined by quantifying luciferase activity in cell lysates. The results of a single representative experiment performed with technical quadruplicates are shown and were confirmed in two separate experiments. Error bars indicate standard errors. Statistical significant differences between luciferase activities measured with pseudotyped viral particles without and with varying doses of G13-CRD_115_ were assessed by a Brown–Forsythe and Welch ANOVA analysis with Dunnett’s T3 multiple comparison test. No significant differences (NS, *p* > 0.05) were observed for G13-CRD_115_ doses of ≤1 nM, but significant differences were observed at doses of ≥5 nM (*p < 0.05; **p < 0.01; ***p < 0.001; ****p < 0.0001). (B) Normalized dose-dependent luciferase activities fitted by eq 1 for G13-CRD_115_ (red curves) and G13-CRD_100_ (black curves), and the fitting parameters are given in Table 1. Significant differences were observed for B.1 at 5 nM (**p < 0.01), Delta at 1 nM (**p < 0.01) and 5 nM (**p < 0.01), and Omicron at 1 nM (***p < 0.001). All other doses gave no significant statistical differences (*p* > 0.05).

The lack of long-term stability for LA-EG_11_-Tz-TFP linker means it has to be prepared fresh each time before lectin conjugation, making its use inconvenient. Therefore, we prepared the more stable LA-EG_11_-TFP linker for protein labeling and G13 conjugation. We prepared G13-CRDs with two CRD valencies, G13-CRD_100_ and G13-CRD_115_. Their inhibition of Vero cell entry of VSV particles pseudotyped with the S proteins of four SARS-CoV-2 variants (i.e., Wuhan wild-type Hu-1, B.1, Delta, and Omicron BA.1) was investigated (Figures 4A and S6.3). Their dose-dependent inhibition data were fitted by eq 1, which yielded comparable low nM EC_50_ values and *n* = 1 for G13-CRD_115_ against pseudotypes bearing the S proteins of all four SARS-CoV-2 variants tested (Figure 4B1–B4 and Table 1). Interestingly, G13-CRD_115_ showed consistently higher potencies (lower EC_50_ values, ∼3- to 4-fold) than G13-CRD_100_ (also yielding *n* = 1 in all inhibition fittings) against all S protein-bearing pseudotypes tested (Table 1 and Figure S6.3), suggesting that a higher CRD valency (larger *D*_h_) improves G13-CRD’s antiviral potency. Moreover, G13-CRD_115_’s inhibitory activity (against pseudotypes bearing B.1 S protein) was significantly and dose-dependently reduced by glycans such as mannose and mannan, which compete with the viral S protein for binding to the DC-SIGN CRD, confirming that the antiviral activity of G13-CRD_115_ originated from specific CRD-sugar binding as proposed (Supporting Information, Section 6 and Figure S6.5).

**Table 1:** Summary of Inhibition Data of G13-CRD_115_ and G13-CRD_100_ against Four Different SARS-CoV-2 Pseudotypes Entry of Vero76 cell (*n* = 1 for Cases)^a^

	G13-CRD_100_ (*D*_h_ ∼22 nm)		G13-CRD_115_ (*D*_h_ ∼26 nm)	
SARS-CoV-2 variant	EC_50_ (nM)	*R*^2^	EC_50_ (nM)	*R*^2^
wild-type (Hu-1)	8.2 ± 1.7	0.933	3.0 ± 0.5	0.943
D614G wild-type (B.1)	7.6 ± 1.3	0.961	2.3 ± 0.6	0.947
Delta (B.1.617.2)	12.1 ± 2.7	0.952	3.0 ± 0.1	0.992
Omicron (BA.1)	6.6 ± 1.3	0.976	1.5 ± 0.3	0.922

a A higher CRD valency and bigger hydrodynamic size appear to boost G13-CRD’s antiviral potency.

### GNP-CRD Inhibition of Authentic SARS-CoV-2 Entry into Vero76 Cells

**Figure 5:**
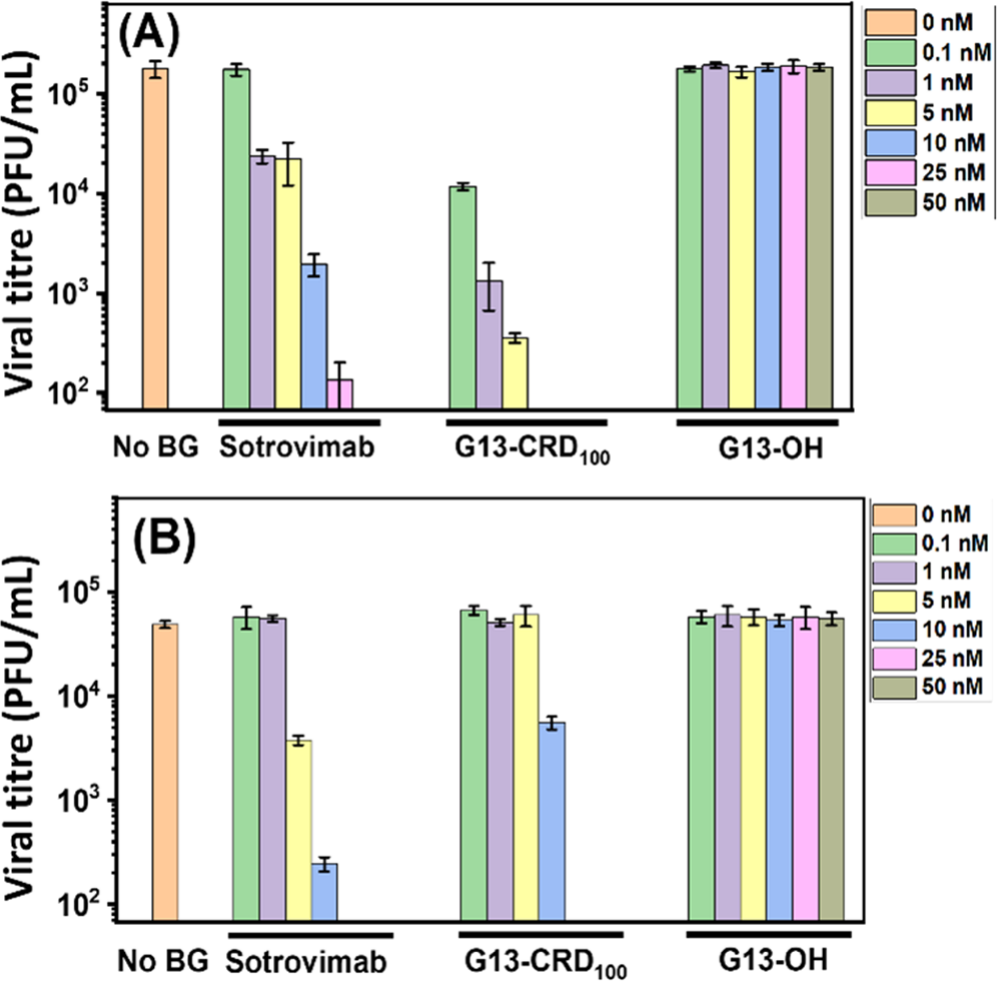
G13-CRD_100_ and Sotrovimab inhibition of authentic (A) early pandemic SARS-CoV-2 (lineage B.1.513) and (B) Omicron BA.1 variant infections. All viral inhibition studies were performed in DMEM containing 10% FBS. The lowest Y-scale indicates the assay limit of detection. The absence of bar chart data at high doses indicates no measurable infection. The bar labeled as No BG indicates the infection level in the absence of inhibitors. The results of a representative experiment performed with technical triplicates are shown and were confirmed in a separate experiment.

The inhibitory effects of G13-CRD_100_ against the authentic early pandemic B.1 and Omicron BA.1 variants were also investigated (Supporting Information, Section 7). Sotrovimab, a clinically approved monoclonal antibody for Covid-19 treatment, was also analyzed as a positive control under identical experimental conditions. G13-CRD_100_ was highly potent against the wild-type virus, resulting in ∼92% inhibition at 0.1 nM (equivalent to ∼9 pM EC_50_ for noncooperative inhibition, *n* = 1) and complete inhibition at 10 nM (Figure 5). In contrast, Sotrovimab was less effective, showing apparently no inhibition at 0.1 nM, although significant inhibition was observed at 1 nM (∼85%) and above. The inhibition data of authentic viruses did not follow the classical potency-dose dependence. The potency increased more rapidly with dose once inhibition was observed, making it difficult to fit the data with inhibition models to derive EC_50_ values. Against Omicron variant BA.1, both Sotrovimab and G13-CRD_100_ showed reduced efficacy, where significant inhibition was observed at 5 and 10 nM, respectively, although they both completely inhibited viral infection at 25 nM. Importantly, the G13-OH control gave no inhibition across the whole concentration range tested (Figure 5), demonstrating that G13-CRD’s inhibitory effect originates from specific CRD-glycan interactions, as expected.

## Discussion

The approved Covid-19 neutralizing Abs or sera from recovered Covid-19 patients or vaccinated individuals were found to be either ineffective or exhibiting greatly reduced potency against the Omicron variants (both pseudotypes and authentic viruses).^12,14^ In contrast, our G13-CRD-based antivirals have demonstrated potent and broad anti-SARS-CoV-2 activity against all four pseudotyped viruses tested. This is attributed to the largely conserved N-glycosylation sites across the S proteins of SARS-CoV-2 variants and careful design of G13-CRD, allowing its flexibly presented CRDs to readily adapt to viral surface glycans to form strong multivalent binding for potent viral neutralization. Viral surface glycans can be heterogeneous, where one N-glycosylation site may be occupied by structurally distinct glycans, and spike protein mutations may alter glycan processing;^15,50^ thus, the ability for CRD to adapt to viral surface glycans is important for viral neutralization. The fact that all four viral variants, in the pseudotype model, were consistently neutralized by G13-CRD with comparable EC_50_ and identical *n* (=1) values clearly demonstrates G13-CRD’s adaptability.

A large potency difference for G13-CRD against the authentic- over the pseudotype- B.1 variant could be due to differences in virion size/shape (∼95 nm sphere,^51^ vs ∼80 nm × 170 nm bullet-shaped^52^), number of S proteins, and inter-S protein spacing. While individual S protein-G13-CRD interactions may be similar, differences in inter-S protein spacing and surface curvature will affect G13-CRD’s ability to crosslink neighboring S proteins on the virion surface, which is critical to interrupt S protein conformational changes and membrane fusion, and hence viral infection. In fact, our G13-CRD was designed to target the inter-S protein spacing (∼30 nm, see Supporting Information, Section 8) of the B.1 variant based on its cryo-EM structure (i.e. ∼40 spikes randomly distributed on a spherical virion of ∼95 nm),^51^ and it exhibited a great potency against this virus (∼9 pM equivalent EC_50_). These results clearly demonstrate the great potential of our design strategy for G13-CRD-based antivirals. The lower potency of Sotrovimab against the authentic BA.1 over B.1 variant is assigned to immune evasion mutations of S proteins in BA.1, which weakened Sotrovimab’s binding affinity and hence neutralizing potency.^14^ This result is fully consistent with the significantly reduced potencies observed for most neutralizing Abs and sera from past infections and/or vaccinations against the Omicron variant over the early pandemic B.1 variant.^12,14,53^

As G13-CRD’s antiviral action is binding to S protein glycans to block viral entry, it was expected to exhibit similar potencies against both the authentic BA.1 and B.1 variants as their S protein glycans are mostly conserved. However, our results show this is not the case, implying that BA.1 variant must have evolved in other ways (besides immune evasion mutations in S proteins) to evade neutralization by G13-CRD. A likely mechanism is altering the number of S proteins (inter-S protein spacing) incorporated into virion particles, making our current G13-CRD no longer spatially matched to crosslink neighboring S proteins for potent neutralization. This is supported by the observation that a potent anti-Ebola virus antibody indeed cross-links neighboring S proteins via its two Fab arms in cryo-EM tomography.^54^ Another potential mechanism could be mutation-induced changes of S protein structure and glycan procession to affect their G13-CRD multivalent binding. Although this is unlikely to be the main reason here because significant potency differences were only observed with the authentic B.1 and BA.1 variants, but not their pseudotype counterparts. Unfortunately, the cryo-EM structure of whole authentic Omicron variant remains to be reported. We therefore call for urgent comparative cryo-EM analysis of authentic SARS-CoV-2 variants with intact S proteins, including the Omicron variants, to help the design of spatially matched polyvalent nano-lectins for potent, specific neutralization of each SARS-CoV-2 variant.

The potential of exploiting multivalency to design potent, broad-spectrum anti-SARS-CoV-2 agents has been demonstrated. Linking IgGs together into an engineered pentameric IgM has shown to not only greatly enhance antiviral potency (up to 230-fold), but also make it insensitive to a range of known immune evasion mutations. The engineered IgM exhibited high potency against several SARS-CoV-2 variants, e.g., B.1.1.7 (α), P.1 (γ), and B.1.351 (β), with *in vivo* rodent models.^55^ The IgM’s superior antiviral property over IgG is assigned to its larger size and higher binding valency, allowing it to bind and crosslink multiple S proteins on virion surfaces that is not possible by individual IgGs. The potential of exploiting multivalent binding in viral neutralization has been further demonstrated with HIV, well known for its ability to evade IgG neutralization, due to its small number of densely glycosylated trimeric spike proteins which effectively prevent both inter- and intra-spike crosslinking by individual IgGs. By linking two Fabs together via a rigid DNA spacer, greatly enhanced anti-HIV potency has been achieved for a Fab dimer having the correct inter-Fab distance for intra-spike crosslinking.^56^ Moreover, an engineered tetravalent DVD antibody (containing four variable domains) displays 100-fold higher potency over its component divalent antibody against Crimean-Congo hemorrhagic fever virus.^57^ These examples, as well as our G13-CRD-based antivirals, clearly demonstrate that exploiting multivalency is a viable antiviral approach. While the immune evasion ability of SARS-CoV-2 variants has been almost exclusively considered on the basis of individual Ab-S protein interactions, we believe contributions from multivalency evasion should also be considered seriously in order to develop more robust antivirals.

Compared to other antiviral agents, our GNP-CRD-based antivirals have several advantages. First, the GNP scaffold size, shape, and lectin valency and flexibility can be easily tuned to match the virus of interest. Second, lectins can be mass- and cheaply produced by recombinant bacterial expression without using animals. Third, viral glycosylation is common and viral glycosylation sites are mostly conserved, and hence may not be strongly affected by viral variations. This makes viral glycans a potentially more robust target for developing antivirals than peptide epitopes targeted by most neutralizing Abs. Finally, our GNP-CRD-based antivirals are particularly useful against viral infections that lack effective neutralizing Abs, or display Ab-enhanced viral infection (e.g., Dengue, Zika).^58,59^ Therefore, we believe the polyvalent nano-lectin-based antivirals reported herein represent a highly attractive, robust, and economical alternative to neutralizing Abs in the fight against a wide range of viral infections. It should also be noticed that, compared to GNP-based antivirals, Abs can also have a few potential advantages, such as a longer blood circulation time (hence less frequent dosages) due to Fc-receptor-mediated recycling *in vivo*, Fc-receptor-activated viral clearance, and Fc-receptor-mediated complement activation, etc.^60,61^ While this current work has established polyvalent nano-lectins as a novel antiviral agent in cell culture, future studies will need to investigate their antiviral potencies, biodistribution, circulation half-time, body clearance, and potential cytotoxicity and long-term toxicity issues with *in vivo* animal models. This information is important to demonstrate their potential as a novel, viable antiviral agent.

## Experimental Section

### Materials

A CdSe/ZnS core/shell quantum dot (QD, λ_EM_ ∼605 nm) coated with mixed ligands of trioctylphosphine oxide (TOPO), hexadecylamine, and oleic acid was purchased from PlasmaChem GmbH (Germany). 2-(2-(2-Chloroethoxy)ethoxy)ethanol (>96%), 2-(2-Aminoethoxy)ethanol (>98%), di-*tert*-butyl dicarbonate (>99%), O-(6-Chlorobenzotriazol1-yl)-*N*,*N*,*N*′,*N*′-tetramethyluronium hexafluorophosphate (HCTU, >98%); tris(2-carboxyethyl)phosphine hydrochloride (TCEP·HCl, >98%); tris[(1-benzyl-1H-1,2,3-triazol-4-yl)methyl]amine (TBTA, >97%), sodium sulfate (>99%), sodium hydride (60% dispersion in mineral oil), 3-bromo-1-propyne (>98%), α-lipoic acid (LA, >98%), copper sulfate (>99%), sodium ascorbate (>98%), anhydrous DMF (>99.8%) and other reagents were purchased from Sigma-Aldrich or Alfa Aesar (U.K.). Azido-EG_11_-amine (>95% monomer purity) and hepta(ethylene glycol) thiol (HS-EG_7_-OH) were purchased from Polypure AS (Norway). 4-Methyltetrazine acid (>95%) and TCO-EG_4_-TFP ester (>95%) were purchased from Click Chemistry Tools. All chemicals and reagents were used as received unless stated otherwise. Solvents (>99%) were purchased from Fischer Scientific (U.K.) and used as received. Anhydrous THF and CH_2_Cl_2_ solvents used in reactions were dried and deoxygenated using a PureSolv solvent purification system (Innovative Technology, Inc.). Ultrapure water (resistance >18.2 MΩ·cm) purified by an ELGA Purelab classic UVF system was used for all experiments and making buffers.

### Methods

All moisture-sensitive reactions were performed under a N_2_ atmosphere. Evaporations were performed under reduced pressure on a BUCHI rotary evaporator. Lyophilization was performed using a Virtis Benchtop K freeze dryer. The progress of the reactions was monitored by TLC on commercially available precoated aluminum plates (Merck silica Kieselgel 60 F254) and stained by either iodine or 10% (v/v) sulfuric acid in ethanol solution, depending on the compound. All ^1^H and ^13^C NMR spectra were recorded in deuterated solvents either on a Bruker AV4 NEO 11.75 T (500 MHz for ^1^H, 125 MHz for ^13^C) or on a Bruker AV3HD 9.4 T (400 MHz for ^1^H, 100 MHz for ^13^C NMR). All chemical shifts (δs) are quoted in parts per million (ppm) downfield of tetramethylsilane, and reference to residual solvent peaks (CDCl_3_: δ ^1^H = 7.26 ppm, δ ^13^C = 77.16 ppm, CD_3_OD: δ ^1^H = 3.31 ppm, δ ^13^C = 49.15 ppm, D_2_O: δ ^1^H = 4.80 ppm) and the coupling constants (J) are reported to the nearest 0.1 Hz. Assignment of spectra was based on expected chemical shifts and coupling constants, aided by COSY, HSQC, and HMBC measurements, where appropriate. The abbreviations used in ^1^H NMR analysis are: s = singlet, br = broad, d = doublet, t = triplet, q = quartet, p = quintet, m = multiplet, dd = doublet of doublets, dt = doublet of triplets, td = triplet of doublets, dq = doublet of quartets, ddd = doublet of doublet of doublets, dtd = doublet of triplet of doublets. High-resolution mass spectra (HRMS) were obtained on a Bruker Daltonics MicroTOF mass spectrometer and the *m*/*z* values were reported in Daltons to four decimal places. UV–vis absorption spectra were recorded on a Varian Cary 50 bio UV–visible spectrophotometer using 1 mL quartz cuvette with an optical path length of 1 cm or on a Nanodrop 2000 spectrophotometer (Thermo Scientific) using 1 drop of the solution with an optical path length of 1 mm. Proteins and gold nanoparticle conjugates were concentrated or purified in Amicon ultra-S2 centrifugal filter tubes with a cut-off MW of 10 and 100 kDa, respectively. Dynamic light scattering (DLS) was measured on a Zetasizer Nano (Malvern) using disposable PMMA cuvettes. The hydrodynamic diameters (*D*_h_s) of the nanoparticles without or with conjugated proteins were measured in water or in a binding buffer (20 mM HEPES, 100 mM NaCl, 10 mM CaCl_2_, pH 7.8). Fluorescence spectra were measured on a Cary Eclipse Fluorescence Spectrophotometer using a 0.70 mL quartz cuvette. All measurements were done in a binding buffer containing 1 mg/mL of bovine serum albumin (BSA) to reduce nonspecific interactions and prevent adsorption of GNP on the surface of cuvette.^62^

### Linker and Ligand Synthesis

The LA-EG_11_-Tz-FTP and LA-EG_11_-TFP linker molecules were synthesized using standard coupling chemistries via the routes shown in Schemes S1 and S2, respectively. The synthesis of LA-EG_4_-DiMan and LA-EG_4_-Gal glycan ligands was described in detail in the Supporting Information, Sections 3.3 and 3.4, respectively. The chemical structures of all key intermediates and final products were confirmed by MS and ^1^H/^13^C NMR spectroscopies. The detailed spectroscopic data for the final linker molecules and glycan ligands are as follows:

#### LA-EG_11_-Tz-FTP

^1^H NMR (CDCl_3_, 500 MHz): δ = 8.57–8.53 (m, 2H), 7.71–7.68 (m, 1H), 7.55–7.51 (m, 2H), 7.00 (tt, 1H, *J* = 9.8, 7.0 Hz), 6.44 (s, 1H), 6.21 (s, 2H), 5.14 (s, 1H), 3.90–3.87 (m, 1H), 3.70–3.61 (m, 53H), 3.60–3.43 (m, 17H), 3.19–3.16 (m, 1H), 3.13–3.09 (m, 4H), 2.96–2.93 (m, 1H), 2.45 (dtd, 2H, *J* = 13.0, 6.6, 5.3 Hz), 2.19 (td, 3H, *J* = 7.5, 1.4 Hz), 1.90 (q, 7H, *J* = 6.9 Hz), 1.72–1.64 (m, 7H), 1.51–1.40 (m, 2H) ppm. ^13^C NMR (125 MHz, CDCl_3_) δ: 172.8(2), 170.9, 170.2 (4× C=O), 167.5, 167.2, 163.9, 156.4, 141.5, 140.2, 135.4, 135.3, 135.0, 134.9, 130.6, 130.2, 129.8, 124.4, 129.4, 128.3, 126.3, 126.2, 103.8, 103.4, 103.3, 103.1, 95.7, 77.2, 70.7(2), 70.6, 70.5, 70.2(2), 69.9, 69.7, 66.1, 56.4, 43.5, 43.4, 40.8, 40.2, 39.5, 39.4, 39.2, 36.3, 36.0, 34.7, 34.5, 28.9, 25.4, 21.3, 21.2, 14.5. HRMS: calculated *m/z* for C_69_H_107_F_4_N_5_NaO_21_S_2_ (M + Na)^+^ 1504.6728; found 1504.6725.

#### LA-EG_11_-TFP

^1^H NMR (500 MHz, CDCl_3_) δ: 6.99 (tt, 1H, *J* = 9.9, 7.0 Hz), 6.67 (s, 1H, amide N*H*), 6.34 (s, 1H, amide N*H*), 3.74–3.70 (m, 1H), 3.66–3.62 (m, 38H, C*H*_2_s in EG_11_ units), 3.55 (td, 4H, *J* = 5.5, 4.5 Hz), 3.46 (dtd, 4H, *J* = 13.1, 5.6, 4.4 Hz), 3.17 (ddd, 1H, *J* = 11.0, 7.1, 5.4 Hz), 3.11 (dt, 1H, *J* = 11.0, 6.9 Hz), 3.04 (t, 2H, *J* = 7.1 Hz), 2.64 (t, 2H, *J* = 7.1 Hz), 2.45 (dtd, 1H, *J* = 13.0, 6.6, 5.4 Hz), 2.19 (td, 2H, *J* = 7.5, 1.4 Hz), 1.90 (dq, 2H, *J* = 12.6, 7.0 Hz), 1.73–1.62 (m, 4H), 1.53–1.40 (m, 2H), 1.25 (s, 1H) ppm; ^13^C NMR (125 MHz, CDCl_3_) δ: 172.9, 170.4, 169.0 (3× C=O), 103.3, 103.1, 103.0, 72.7, 70.6, 70.5(2), 70.4, 70.2, 70.1, 69.9, 69.8, 61.6, 56.4, 40.2, 39.4, 39.2, 38.4, 36.3, 34.7, 30.4, 29.7, 28.9, 28.8, 25.4 ppm. HRMS: calculated *m/z* for C_42_H_69_F_4_N_2_O_15_S_2_ (M + H)^+^ 981.4075; found 981.4098.

#### LA-EG_4_-Gal

^1^H NMR (D_2_O, 500 MHz): δ = 8.11 (s, 1H, triazole-H), 4.70 (s, 2H), 4.65 (t, 2H, *J* = 5.1 Hz), 4.40 (d, 1H, *J* = 7.9 Hz, H-1), 3.99 (t, 2H, *J* = 5.0 Hz), 3.92 (d, 1H, *J* = 3.4 Hz), 3.82–3.58 (m, 28H, CH_2_s in EG_n_ units), 3.56–3.50 (m, 2H), 3.38 (t, 2H, *J* = 5.3 Hz), 3.26–3.15 (m, 2H), 2.48 (dq, 1H, *J* = 12.2, 6.0 Hz), 2.25 (t, 2H, *J* = 7.3 Hz), 2.00–1.94 (m, 1H), 1.78–1.69 (m, 1H), 1.66-1.60 (m, 3H), 1.44–1.38 (p, 2H, *J* = 7.6 Hz) ppm. ^13^C NMR (D_2_O, 125 MHz): δ = 176.9 (C=O), 102.8 (C-1), 75.1, 72.7, 70.7, 69.7(2), 69.6(3), 69.5(2), 69.4, 69.2, 68.9, 68.8, 68.7, 68.6(2), 63.1, 60.9, 56.5, 50.1, 50.0, 40.2, 38.9, 38.0, 35.4, 33.7, 27.8, 25.0 ppm. HRMS: Expected C_31_H_56_N_4_O_13_S_2_*m/z* 757.3319, found 757.3380.

#### LA-EG_4_-DiMan

^1^H NMR (D_2_O, 400 MHz): δ = 8.11 (s, 1H, triazole-H), 5.12 (s, 1H, Man H-1′), 5.03 (s, 1H, Man H-1), 4.71 (s, 2H), 4.66 (t, 2H, *J* = 5.1 Hz), 4.08 (d, 1H, *J* = 3.1 Hz), 3.99 (m, 3H), 3.95–3.83 (m, 5H), 3.82–3.60 (m, 28H, CH_2_s in EG_n_ units), 3.40 (t, 2H, *J* = 5.3 Hz), 3.27-3.15 (m, 2H), 2.49 (m, 1H), 2.26 (t, 2H, *J* = 7.3 Hz), 1.98 (m, 1H), 1.75 (m, 1H), 1.64 (dd, 3H, *J* = 14.0, 7.6 Hz), 1.41 (p, 2H, *J* = 7.7 Hz) ppm. ^13^C NMR (D_2_O, 100 MHz): δ = 176.9 (C=O), 143.9 (C=CH), 125.5 (C=CH), 102.3 (Man C-1), 98.4 (Man-C-1′), 78.6, 73.3, 72.7, 70.3, 70.2, 69.9, 69.6, 69.5, 69.4, 69.0, 68.9, 68.8, 66.9, 66.5, 63.1, 61.1, 60.89, 56.5, 50.0, 40.3, 38.9, 38.1, 35.5, 33.7, 27.8, 25.0 ppm. HRMS: calculated *m/z* for C_37_H_67_N_4_O_18_S_2_ (M + H)^+^ 919.3886; found 919.3899.

### N-Terminal Linker Labeling of DC-SIGN ECD or CRD

DC-SIGN tetrameric ECD and its monomeric CRD were expressed in *E. coli* and purified by sepharose-mannose affinity column, and their concentrations were estimated from the UV absorbance at 280 nm using an extinction coefficient of 281 600 and 52 980 M^–1^ cm^–1^ for ECD and CRD, respectively.^39−41^ Proteins were then dissolved in a low pH labeling buffer (20 mM HEPES, 150 mM NaCl, and 10 mM CaCl_2_, pH 6.2) to ensure that only N-terminal amine is nonprotonated and is available for labeling.^44^ This was achieved by adding LA-EG_11_-Tz-TFP or LA-EG_11_-TFP linker (in dry DMSO) to the ECD or CRD in the labeling buffer at a linker:ECD monomer or CRD molar ratio of 1.5:1. The mixture was mixed on a rotating mixer at room temperature for 40 min, and then diluted with the binding buffer (20 mM HEPES, 100 mM NaCl, 10 mM CaCl_2_, pH 7.8). Any unlabeled free linker molecules were removed by washing with the binding buffer using a 10 kDa MWCO ultrafiltration unit. HRMS analysis revealed that the labeling mixture contained both singly labeled and unlabeled proteins. Using the relevant peak areas of each species, the single-linker labeling efficiencies for LA-EG_11_-Tz-TFP linker were estimated as 18 and 22% for ECD and CRD (denoted as LA-EG_11_-Tz-ECD and LA-EG_11_-Tz-CRD), respectively (Figure S4.1) with no doubly labeled species. While for the LA-EG_11_-TFP linker, the single-linker labeling efficiency was estimated as 19% with a small amount (∼2%) of double-linker-labeled species, denoted as LA-EG_11_-CRD and (LA-EG_11_)_2_-CRD, respectively (see Figure S4.2).

### Preparation of Antiviral Polyvalent Nano-Lectins

Citrate-stabilized 13 nm gold nanoparticles (G13) were prepared by citrate reduction of HAuCl_4_ following our established procedures.^45^ Its concentration was calculated by the Beer–Lambert law using peak absorbance at 519 nm and an extinction coefficient of 2.32 × 10^8^ M^–1^ cm^–1^.^45^ To prepare antiviral nano-lectins, G13 was first partially PEGylated to enhance its stability in the binding buffer. This was achieved by incubating citrate-stabilized G13 with 2000 molar equivalent of HS-EG_7_-OH in an aqueous solution under stirring for 48 h at room temperature. The resulting G13 dispersion was concentrated using 100 kDa MWCO filter tubes and washed with 100 mL of pure water to remove any unbound free ligands. This yielded partially PEGylated G13 (denoted as ppG13-OH) which was found to be highly stable and monodisperse in the binding buffer. A fully PEGylated G13 negative control (G13-OH) was also prepared by incubating citrate-stabilized G13 with 5000 molar equivalent of HS-EG_7_-OH using the same conditions as that of ppG13-OH preparation.

The partially PEGylated ppG13-OH in pure water was added 1/4 of its volume of a 5× binding buffer (100 mM HEPES, 750 mM NaCl, 50 mM CaCl_2_, pH 7.8) to make it in final 1× binding buffer. Then, the linker-labeled ECD or CRD was added to ppG13-OH (in 1× binding buffer) at a linker-labeled protein:ppG13-OH molar ratio of 100:1 for LA-EG_11_-Tz-ECD, LA-EG_11_-Tz-CRD, and LA-EG_11_-CRD. To investigate how the CRD:G13 molar ratio affects conjugation and viral inhibition, another batch of G13-CRD was prepared at an LA-EG_11_-CRD:ppG13-OH molar ratio of 115:1. The resulting mixed solution was stirred at 4 °C overnight (∼16 h) and then transferred to a 100 kDa MWCO ultrafiltration tube and centrifuged to collect the G13-lectin conjugates. The flow through filtrate was collected and analyzed by HRMS. Only unlabeled ECD or CRD was detected in all of the filtrates (Figure S5.1), suggesting that all linker-labeled ECD or CRD were bound to G13. Thus, G13-Tz-ECD, G13-Tz-CRD, and G13-CRD prepared under a linker-labeled protein:G13 molar ratio of 100 and 115 should have a lectin valency of ∼100 and ∼115 per G13, abbreviated as G13-Tz-ECD_100_, G13-Tz-CRD_100_, G13-CRD_100_, and G13-CRD_115_, respectively. The resulting G13-lectin conjugates were washed three times with binding buffer using the same 100 kDa MWCO ultrafiltration tube, before being transferred to sample vials. The concentration of each G13-lectins was calculated from its UV absorbance at 520 nm using G13’s extinction coefficient (2.32 × 10^8^ M^–1^·cm^–1^).

### Inhibition of Pseudo-SARS-CoV-2 Infection

Vesicular stomatitis virus (VSV) particles pseudotyped with SARS-CoV-2 S protein and encoding a luciferase gene were generated as described previously.^8,9^ We and others have shown previously that these particles adequately model SARS-CoV-2 entry into cells and its inhibition.^9,12−14^ All cell culture was performed in Dulbecco’s modified Eagle medium (DMEM) (PAN-Biotech, Aidenbach, Germany), supplemented with 10% fetal bovine serum (FBS) (Biochrom Berlin, Germany) and penicillin (100 U/mL)/streptomycin (0.1 mg/mL) solution (P/S) (PAA Laboratories GmbH, Cölbe, Germany) as reported previously.^9^

To evaluate G13-CRD’s inhibitory effect on SARS-CoV-2 S protein-driven cell entry, Vero76 cells were seeded in 96-well plates at a density of 2 × 10^5^ cells per well. Equal volumes of pseudotype preparations and G13-CRD were incubated in DMEM containing 10% FBS at 37 °C for 2 h. Medium was aspirated from the cells (at 24 h post seeding), then pseudotype viral particles and G13-CRD mixture (100 μL) were added to each well, and cells were incubated at 37 °C for 16–18 h. After that, the cell medium was removed and cells were lysed using PBS supplemented with 0.5% Triton X-100 (Carl Roth, Germany) for 30 min at RT. Then 30 μL of cell lysates were transferred into white 96-well plates, mixed with luciferase substrate (Beetle-Juice, PJK) and then luminescence signals were measured with a Hidex Sense Plate luminometer (Hidex). The luciferase activities in cell lysates from each treatment were normalized against the corresponding control measured in the absence of G13-CRD. The normalized infection (NI)–*C* relationship was fitted by the modified inhibition model (eq 1) to derive the apparent viral inhibition potencies (EC_50_ and *n* values) as described in the main text.

The same protocol was used to evaluate how DC-SIGN-binding glycan molecules (mannose, glucose, and mannan) may compete with pseudotypes bearing SARS-CoV-2 (B.1 variant) S protein for binding to G13-CRD_115_ (see Figure S6.5A), thereby reducing G13-CRD_115_ ability to inhibit viral transduction. Each glycan competitor (various doses) was pre-incubated with G13-CRD_115_ (50 nM final dose) at 37 °C for 1 h before being added to B.1 pseudotype particles and further incubated for 2 h at 37 °C. Finally, the B.1 pseudotype/glycan/G13-CRD1 mixture was added to Vero76 cells to evaluate their antiviral properties using the same steps as above.

### Inhibition of Authentic SARS-CoV-2 Infection

All work with infectious SARS-CoV-2 was conducted under BSL-3 conditions at the German Primate Centre, Göttingen, Germany. Vero76 cells were seeded in 96-well plates at a density of 2 × 10^5^ cells per well. Different doses (ranging from 0.1 to 50 nM) of G13-CRD_100_, G13-OH, or Sotrovimab (kindly provided by Sebastian Schulz and Hans-Martin Jäck from Friedrich-Alexander University of Erlangen-Nürnberg, Germany) were each incubated with SARS-CoV-2 isolate NK, Pango lineage B.1.513 (kindly provided by Stephan Ludwig, Institute of Virology, University of Münster, Münster, Germany) or SARS-CoV-2 isolate Omicron BA.1, Pango lineage BA.1 (kindly provided by Christian Drosten, Institute of Virology, Charité-Universitätsmedizin Berlin, Germany) at 37 °C for 2 h in an inoculation volume of 100 μL. Afterward, Vero76 cells were infected with the virus-inhibitor mixtures at an MOI of 0.01 at 37 °C. After 1 h incubation, the inoculum was removed, cell cultures were washed with PBS two times, and 100 μL of culture medium was added to the cells. Supernatants were collected at 0 and 48 h post infection (hpi) and stored at −80 °C until further usage. Viral titers were determined by plaque assay on Vero76 cells as described previously,^9,14^ and are given as PFU/mL.

### Statistical Analysis

Microsoft Excel (as part of the Microsoft Office software package, version 2019, Microsoft Corporation) and GraphPad Prism 9 version 9.0.2 (GraphPad Software) were used to analyze the data. Statistical analysis was carried out by a Brown–Forsythe and Welch ANOVA analysis with Dunnett’s T_3_ multiple comparison test. Only *p*-values of 0.05 or less were considered to be statistically significant. NS (not significant) *p* > 0.05; *p ≤ 0.05; **p ≤ 0.01; ***p ≤ 0.001; ****p ≤ 0.0001.

## Supporting Information Available

Materials and instruments, production and characterization of DC-SIGN ECD and CRD, chemical synthesis and characterization, ECD and CRD N-terminal labeling with LA-EG_11_-Tz-TFP or LA-EG_11_-TFP, preparation and modification of 13 nm diameter gold nanoparticle (G13), GNP-CRD characterization, pseudo-SARS-CoV-2 virus preparation and inhibition studies, inhibition of authentic SARS-CoV-2 infection by G13-CRD, description of software and methods used for statistical analysis, and estimation of inter-spike distance on virion surface (PDF)
