# Supplementary material for: Polyvalent Nano-Lectin Potently Neutralizes SARS-CoV-2 by Targeting Glycans on the Viral Spike Protein
Source: JACS Au. 2023 Jun 12;3(6):1755–66. doi: 10.1021/jacsau.3c00163 (PMC10302749; doi:10.1021/jacsau.3c00163)
Supplement: Supplementary file 1 — au3c00163_si_001.pdf [file au3c00163_si_001.pdf]

Supplementary Information

A Polyvalent Nano-Lectin Potently Neutralizes SARS-CoV-2 by Targeting Glycans on the Viral Spike Protein

Darshita Budhadev,[1,5] James Hooper,[2,5] Cheila Rocha,[3,4,5] Inga Nehlmeier,[3] Amy Madeleine Kempf,[3,4] Markus Hoffmann,[3,4] Nadine Krüger,[3] Dejian Zhou,[1]* Stefan Pöhlmann,[3,4]* Yuan Guo[2]*

[1] School of Chemistry and Astbury Centre for Structural Molecular Biology, University of Leeds, Leeds LS2 9JT, United Kingdom.

[2] School of Food Science & Nutrition and Astbury Centre for Structural Molecular Biology, University of Leeds, Leeds LS2 9JT, United Kingdom.

[3] Infection Biology Unit, German Primate Center - Leibniz Institute for Primate Research, Göttingen, 37077, Germany

[4] Faculty of Biology and Psychology, Georg-August-University Göttingen, Göttingen 37073, Germany.

[5] These authors contributed equally: Darshita Budhadev, James Hooper, Cheila Rocha,

Emails of Corresponding authors

Dejian Zhou: d.zhou@leeds.ac.uk.

Stefan Pöhlmann: SPoehlmann@dpz.eu.

Yuan Guo: y.guo@leeds.ac.uk.

Table S1. Summary of the SARS-CoV-2 S protein glycosylation sites on a recombinant S protein expressed from human embryonic kidney 293F cells. Figure was redrawn based on the literature data (Watanabe et al. *Science* **2020**, *369*, 330). The S1 subunit contains the angiotensin-converting enzyme 2 receptor (ACE2) binding domain, red bar), and the S2 subunit contains the fusion peptide (FP, blue bar) and the transmembrane segment (TM, grey bar). The color codes for the N-linked glycans are: oligomannose (green); complex (dark red) and hybrid (black). The oligomannose-type glycans are found in certain positions and its expression is largely independent of the expressing system, and is closely related to the protein structure and glycan density.

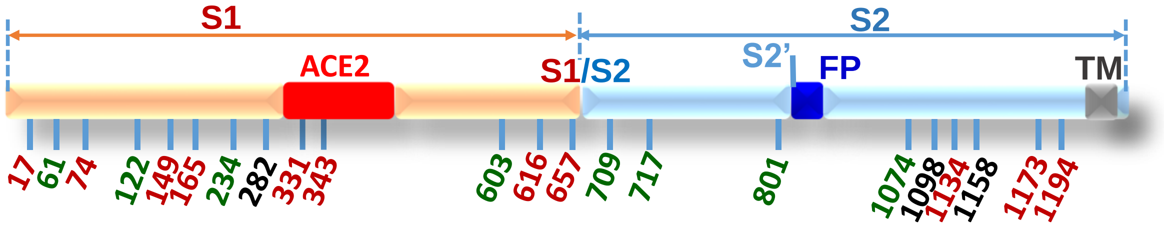

Key mutation sites on S protein of different SARS-CoV-2 variants (data obtained from https://viralzone.expasy.org/9556).

WHO label	Lineage	Spike mutations or deletions (Δ)
Alpha	B.1.1.7	Δ69-70; Δ144; N501Y; A570D; D614G; P681H; T716I; S982A; D1118H
Beta	B.1.351	D80A; D215G; Δ241-243; K417N; E484K; N501Y; D614G; A701V
Gamma	P1	L18F; T20N, P26S, D138Y; R190S; K417N/T; E484K; N501Y; D614G; H655Y; T1027I; V1176F
Delta	B.1.617.2	T19R; G142D; Δ156-157; R158G; L452R; T478K; D614G; P681R; D950N
Omicron	B.1.1.529<br>BA.1	A67V; Δ69-70; T95I; GVYY142-145D; NL211-212I; ins214EPE; G339D; S371L; S373P; S375F; K417N; N440K; G446S; S477N; T478K; E484A; Q493R; G496S; Q498R; N501Y; Y505H; T547K; D614G; H655Y; N679K; P681H; N764K; D796Y; N856K; Q954H; N969K; L981F
	BA.2	T19I; LPPA24-27S; G142D; V213G; G339D; S371F; S373P; S375F; T376A; D405N; R408S; K417N; N440K; S477N; T478K; E484A; Q493R; Q498R; N501Y; Y505H; D614G; H655Y; N679K; P681H; N764K; D796Y; Q954H; N969K
	BA.4	T19I; LPPA24-27S; Δ69-70; G142D; V213G; G339D; S371F; S373P; S375F; T376A; D405N; R408S; K417N; N440K; L452R; S477N; T478K; E484A; F486V; Q498R; N501Y; Y505H; D614G; H655Y; N679K; P681H; N764K; D796Y; Q954H; N969K
	BA.5	T19I; LPPA24-27S; Δ69-70; G142D; V213G; G339D; S371F; S373P; S375F; T376A; D405N; R408S; K417N; N440K; L452R; S477N; T478K; E484A; F486V; Q498R; N501Y; Y505H; D614G; H655Y; N679K; P681H; N764K; D796Y; Q954H; N969K

1. Materials and Instruments

2-(2-(2-Chloroethoxy)ethoxy)ethanol (>96%), 2-(2-Aminoethoxy)-ethanol (>98%), di-tert-butyl dicarbonate (>99%), O-(6-Chlorobenzotriazol1-yl)-N,N,N′,N′-tetramethyluronium hexafluorophosphate (HCTU, >98%); tris(2-carboxyethyl)phosphine hydrochloride (TCEP.HCl, >98%); tris[(1-benzyl-1H-1,2,3-triazol-4-yl)methyl]-amine (TBTA, >97%), sodium sulfate (>99%), sodium hydride (60% dispersion in mineral oil), 3-bromo-1-propyne (>98%), potassium hydroxide (>99%), trifluoroacetic acid (>99%), triethylamine (>99%), sodium bicarbonate, DL-lipoic acid (LA, >98%), copper sulfate (>99%), sodium ascorbate (>98%), tris(hydroxyl-methyl)aminomethane (Tris base, >99%), guanidine hydrochloride (>99%), anhydrous DMF (>99.8%) and other reagents were purchased commercially from Sigma Aldrich or Alfa Aesar (UK). Compound **1** (azido-EG_{11}-amine, >95% monomer purity) and hepta(ethylene glycol) thiol (HS-EG_7-OH) were purchased from Polypure AS (Norway). Compounds **4** (Methyltetrazine acid, >95%) and **6** (TCO-EG_4-TFP ester, >95%) were purchased from Click Chemistry Tools (U.S.A.). All chemicals and reagents were used as received without further purification unless specified elsewhere. All the solvents of >99% purity were purchased from Fischer Scientific (UK) and used as received. Anhydrous THF and CH_2Cl_2 solvents used in reactions were dried and deoxygenated using a PureSolv® solvent purification system (Innovative Technology Inc). Ultra-pure water (resistance >18.2 MΩ.cm) purified by an ELGA Purelab classic UVF system, was used for all experiments and making buffers.

All moisture-sensitive reactions were performed under a N_2 atmosphere using oven-dried glasswares. Evaporations were performed under reduced pressure on a BUCHI rotary evaporator. Lyophilisation was performed using a Virtis Benchtop K freeze dryer. The progress of the reactions was monitored by TLC on commercially available precoated aluminium plates (Merck silica Kieselgel 60 F254) and stained by either iodine or 10% (v/v) sulphuric acid in ethanol solution depending on the compound. All ^{1}H and ^{13}C NMR spectra were recorded in deuterated solvents either on a Bruker AV4 NEO 11.75 T (500 MHz for ^{1}H, 125 MHz for ^{13}C) or on a Bruker AV3HD 9.4 T (400 MHz for ^{1}H, 100 MHz for ^{13}C NMR). All chemical shifts (δs) are quoted in parts per million (ppm) downfield of tetramethylsilane and reference to residual solvent peaks ($CDCl_3$: δ ^{1}H = 7.26 ppm, δ ^{13}C = 77.16 ppm, CD_3OD: δ ^{1}H = 3.31 ppm, δ ^{13}C = 49.15 ppm, D_2O: δ ^{1}H = 4.80 ppm) and the coupling constants (J) are reported to the nearest 0.1 Hz. Assignment of spectra was based on expected chemical shifts and coupling constants, aided by COSY, HSQC and HMBC measurements, where appropriate. The abbreviations used in ^{1}H NMR analysis are: s = singlet, br = broad, d = doublet, t = triplet, q = quartet, p = quintet, m = multiplet, dd = doublet of doublets, dt = doublet of triplets, td = triplet of doublets, dq= doublet of quartets, ddd = doublet of doublet of doublets, dtd = doublet of triplet of doublets. High-resolution mass spectra (HR-MS) were obtained on a Bruker Daltonics MicroTOF mass spectrometer and the m/z values were reported in Daltons to four decimal places. UV-vis absorption spectra were recorded on a Varian Cary 50 bio UV-Visible Spectrophotometer over 200-800 nm using 1 mL quartz cuvette with an optical path length of 1 cm or on a Nanodrop 2000 spectrophotometer (Thermo Scientific) over the range of 200-800 nm using 1 drop of the solution with an optical path length of 1 mm. The protein concentrating and gold nanoparticle conjugates purification were performed in Amicon ultra-S2 centrifugal filter tubes with a cut-off MW of 10kDa and 100kDa, respectively. Dynamic light scattering (DLS) was measured on a Zetasizer Nano (Malvern) using disposable PMMA cuvettes. The hydrodynamic diameters (D_hs) of the GNPs without or with the protein conjugated were measured in water or in a binding buffer (20 mM HEPES, 100 mM NaCl, 10 mM $CaCl_2$, pH 7.8). Fluorescence spectra were measured on a Cary Eclipse Fluorescence Spectrophotometer using a 0.70 mL quartz cuvette. All measurements were done in a binding buffer (20 mM HEPES, 100 mM NaCl, 10 mM $CaCl_2$, pH 7.8) containing 1 mg/mL of bovine serum albumin (BSA) to reduce non-specific interactions and prevent adsorption of GNP on the surface of cuvette.

2. Production and characterization of DC-SIGN ECD and CRD

DC-SIGN homotetrameric extracellular domain (ECD) and its monomeric carbohydrate recognition domain (CRD) were expressed in *E. coli* (BL21/DE3) and purified by sepharose-mannose affinity chromatography as described previously.[1,2] The identity and purity of both proteins are confirmed by High Resolution Mass spectroscopy (HR-MS, as shown in **Figure S1** below). Calculated molecular weights (MWs) are 39197.22 for ECD monomer (oligomeric proteins are denatured into monomer under MS conditions), 17404.25 for CRD and 17804.25 for CRD+Ca^{2+}, respectively. MWs found from HRMS are 39201.68 for ECD monomer and 17793.32 for CRD+Ca^{2+}, respectively. The protein concentrations were estimated from their UV absorbance at 280 nm using an extinction coefficient of 281,600 and 52,980 M^{-1} cm^{-1} for ECD and CRD, respectively.[1,2]

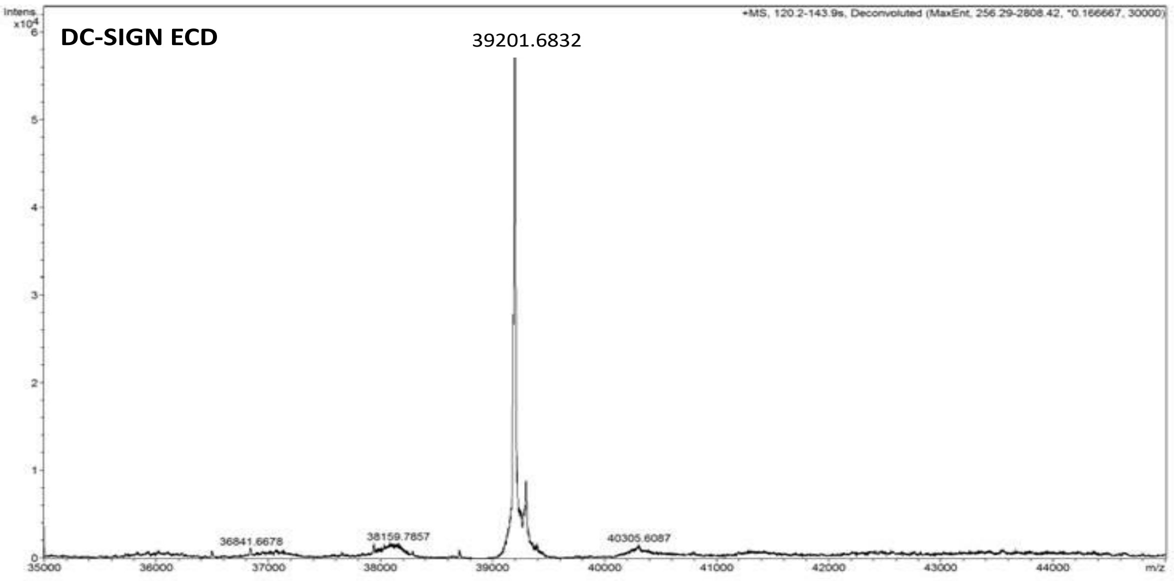

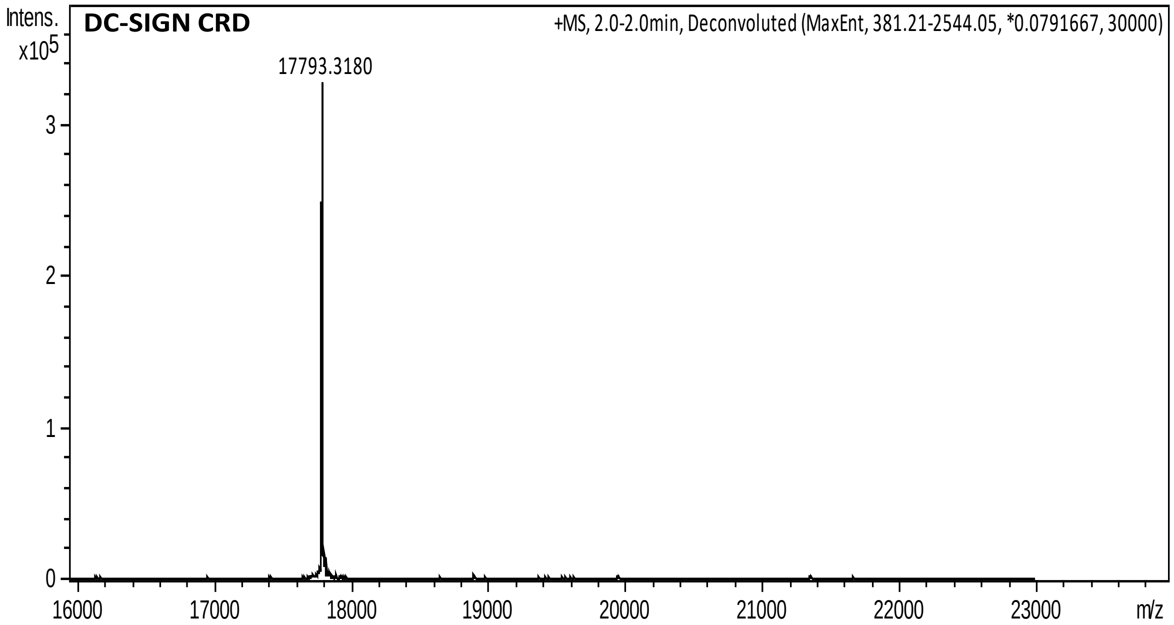

Figure S1. De-convoluted HRMS spectra of DC-SIGN ECD (top) and CRD (bottom) confirming that the correct proteins are obtained.

3. Chemical synthesis and characterization

3.1 Synthesis of linker 1: LA-EG$_{11}$-Tz-TFP (compound 7):

The synthetic route to compound 7 is illustrated in Scheme S1 below:

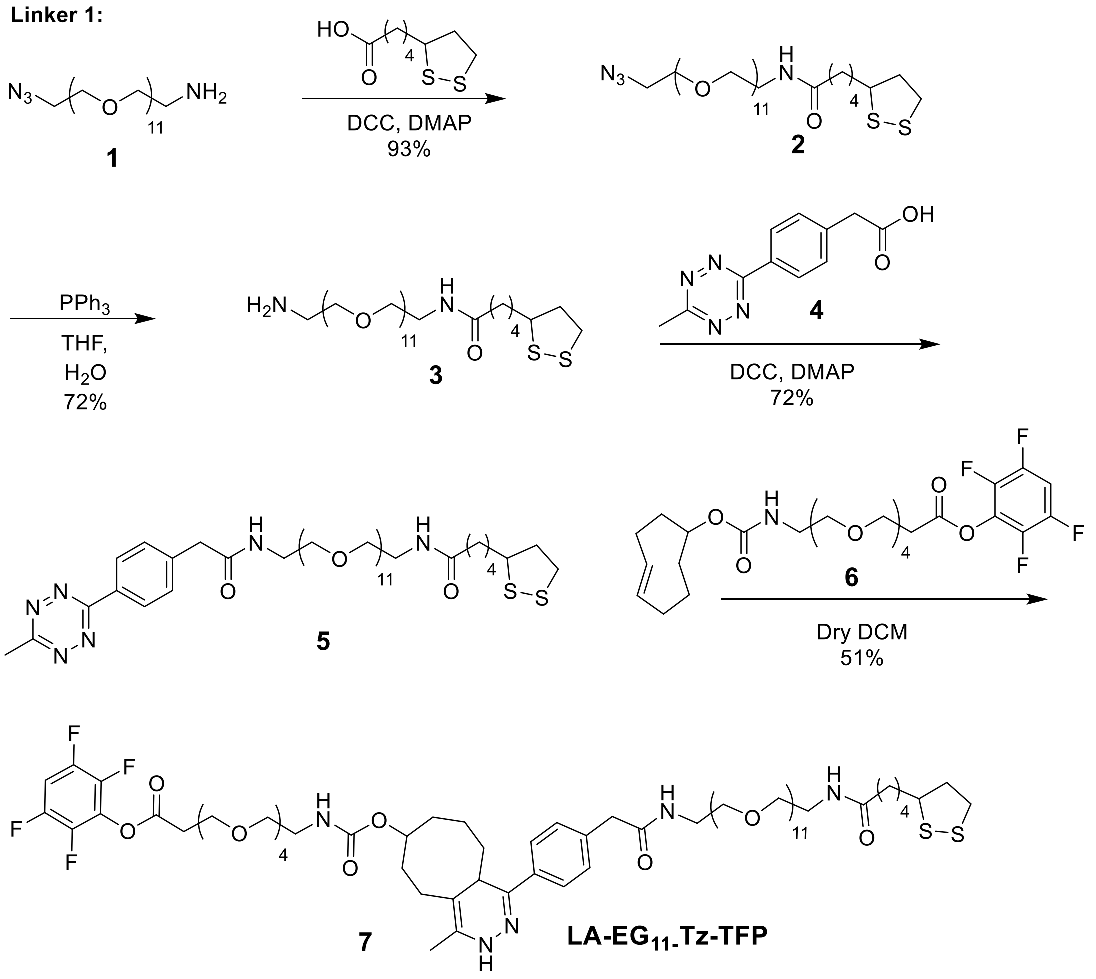

Scheme S1. Synthetic route to the LA-EG$_{11}$-Tz-TFP linker molecule.

LA-EG$_{11}$-N$_3$ (compound **2)**

To an anhydrous CH_2Cl_2 solution (20 mL) of H_2N-EG$_{11}$-N_3, **1** (1.0 g, 4.58 mmol), lipoic acid, **4** (0.95g, 4.58 mmol) and DMAP (0.11g, 0.92 mmol) were added, and the reaction mixture was stirred at 0 °C on an ice bath under nitrogen for 15 min. To this reaction mixture, DCC (1.13g, 5.50 mmol) dissolved in anhydrous CH_2Cl_2 (5 mL) was added dropwise over 20 min and stirred at 0 °C for one hour before being allowed to gradually warm up to room temperature and stirred overnight. Upon completion, the reaction mixture was filtered over celite, and the solvent evaporated to afford the crude product, which was then purified by flash silica chromatography ($CHCl_3$: CH_3OH = 20:1) to furnish product **2** (1.24g, 93%) as a yellowish oil.

^{1}H NMR (CDCl$_3$, 500 MHz): δ = 6.44 (1H, *amide NH*), 3.70 – 3.63 (m, 40H, C*H$_2$*s in EG$_{11}$ unit), 3.61 – 3.58 (m, 2H), 3.45 (dd, 2H, *J*=5.5, 4.4 Hz), 3.41 (t, 2H, *J*=5.1 Hz), 3.17 (ddd, 1H, *J*=11.0, 7.1, 5.4 Hz), 3.10 (dt, 1H, *J*=11.0, 6.9 Hz), 2.45 (dtd, 1H, *J*=13.0, 6.6, 5.3 Hz), 2.19 (td, 2H, *J*=7.5, 1.3 Hz), 1.93 – 1.86 (m, 2H), 1.72 - 1.65 (m, 4H), 1.52 – 1.40 (m, 2H) ppm. ^{13}C NMR (125 MHz, CDCl$_3$) δ: 172.9 (C=O), 70.7(2), 70.6(2), 70.5(2), 70.2, 70.0(2), 56.5, 50.7, 40.2, 39.2, 38.5, 36.3, 34.7, 28.9, 25.4 ppm. **HRMS:** calculated *m/z* for $C_{32}H_{66}N_5O_{12}S_2$ $(M+NH_4)^+$ 776.4144; found 776.4161.

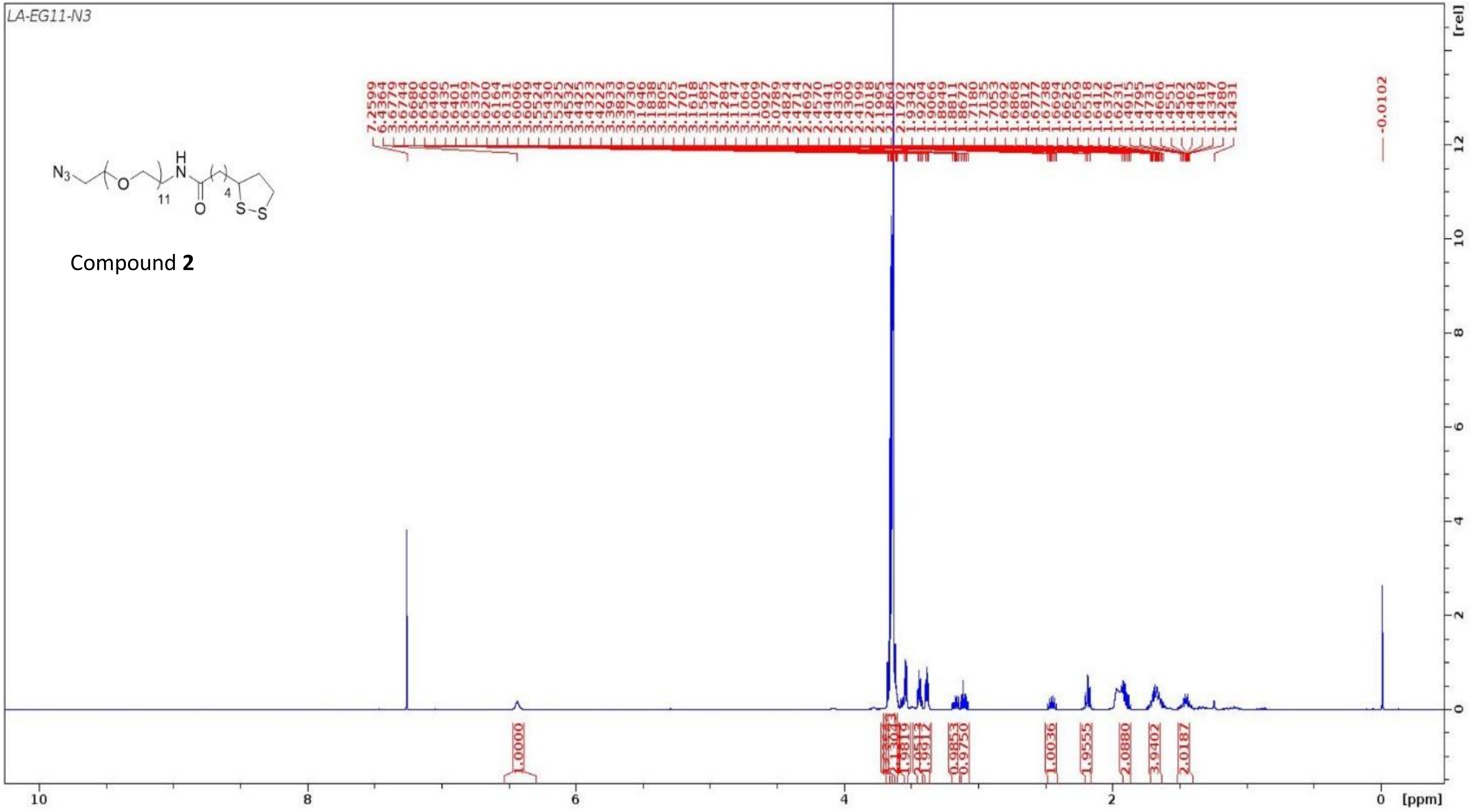

Compound **2**

The ^{1}H NMR spectrum of compound **2** in $CDCl_3$

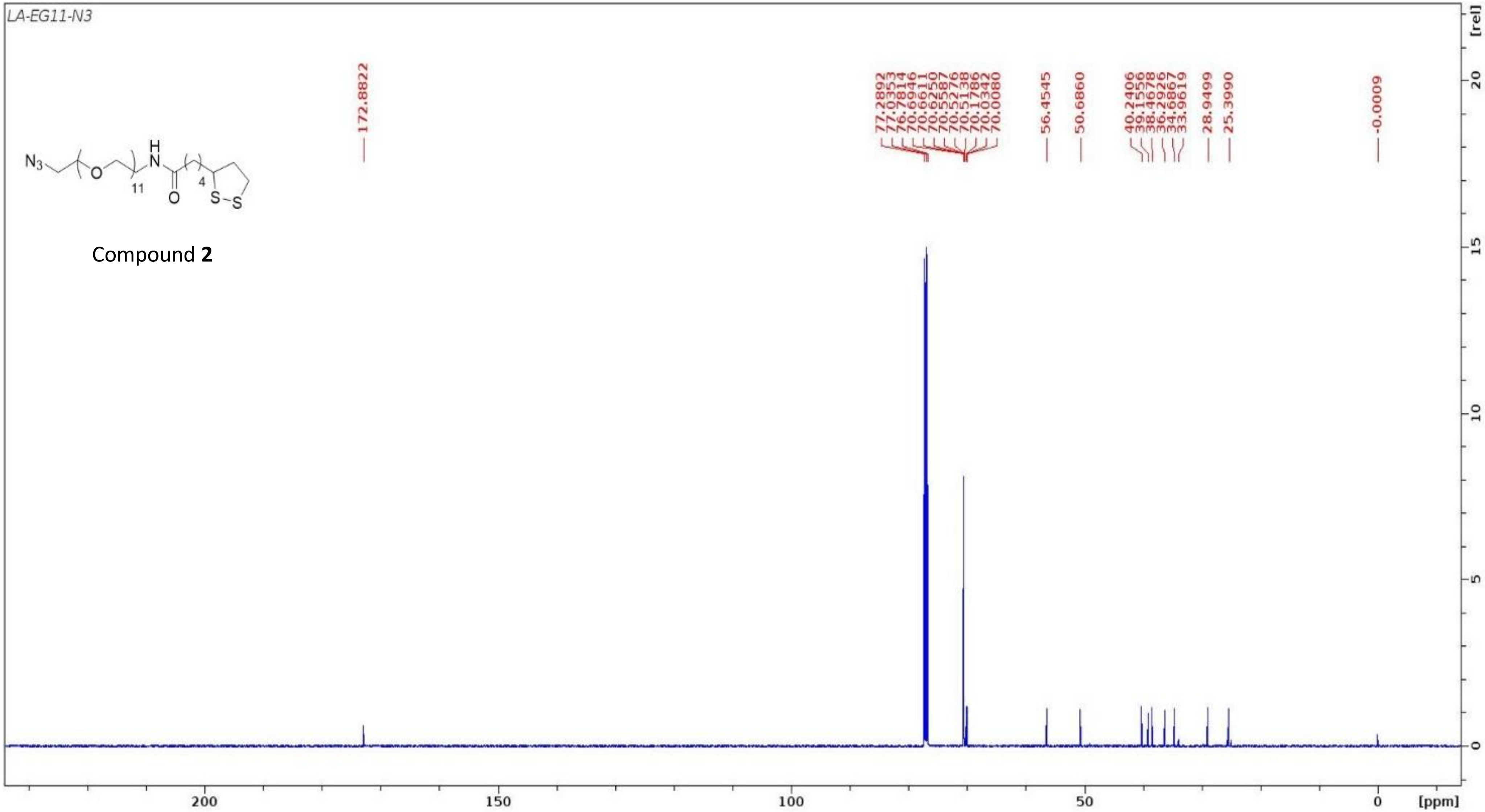

The ^{13}C NMR spectrum of compound 2 in $CDCl_3$

LA-EG_{11}-NH_2 (compound 3):

Compound **2** (1.24g, 1.64 mmol) and PPh_3 (0.86 g, 3.28 mmol) were dissolved in anhydrous THF (30 mL) and stirred under nitrogen at room temperature for 30 min. After that, deionized water (0.60 mL, 32.7 mmol) was added, and the reaction was stirred for further 20 hours till TLC ($CHCl_3$:CH_3OH = 4:1) confirmed the complete reduction of the azide. The solvents were then evaporated, and the crude product was purified using silica gel chromatography (CH_2Cl_2: CH_3OH: ammonia= 8: 1: 0.1) to yield product **3** as a yellow oil (0.86g, 72%).

^{1}H NMR ($CDCl_3$, 500 MHz): δ = 6.20 (1H, *amide NH*), 3.65 – 3.59 (m, 40H, C*H*$_2$s in the EG_{11} unit), 3.57 – 3.49 (m, 5H), 3.43 (td, 2H, *J*=5.6, 4.5 Hz), 3.20 – 3.13 (m, 1H), 3.09 (dt, 1H, *J*=11.0, 6.9 Hz), 2.89 – 2.85 (m, 2H), 2.44 (dtd, 1H, *J*=13.0, 6.6, 5.3 Hz), 2.17 (td, 4H, *J*=7.4, 1.4 Hz), 1.89 (dq, 1H, *J*=12.7, 7.0 Hz), 1.73 – 1.58 (m, 4H), 1.50 – 1.38 (m, 2H) ppm. ^{13}C NMR (125 MHz, $CDCl_3$) δ: 172.8 (C=O), 77.3, 72.9, 70.6(2), 70.6, 70.5, 70.3, 70.2, 69.9, 56.4, 41.7, 40.2, 39.2, 38.5, 36.3, 34.7, 28.9, 25.4 ppm. **HRMS:** calculated *m/z* for $C_{32}H_{64}N_2NaO_{12}S_2$ $(M+Na)^+$ 755.3793; found 755.3792.

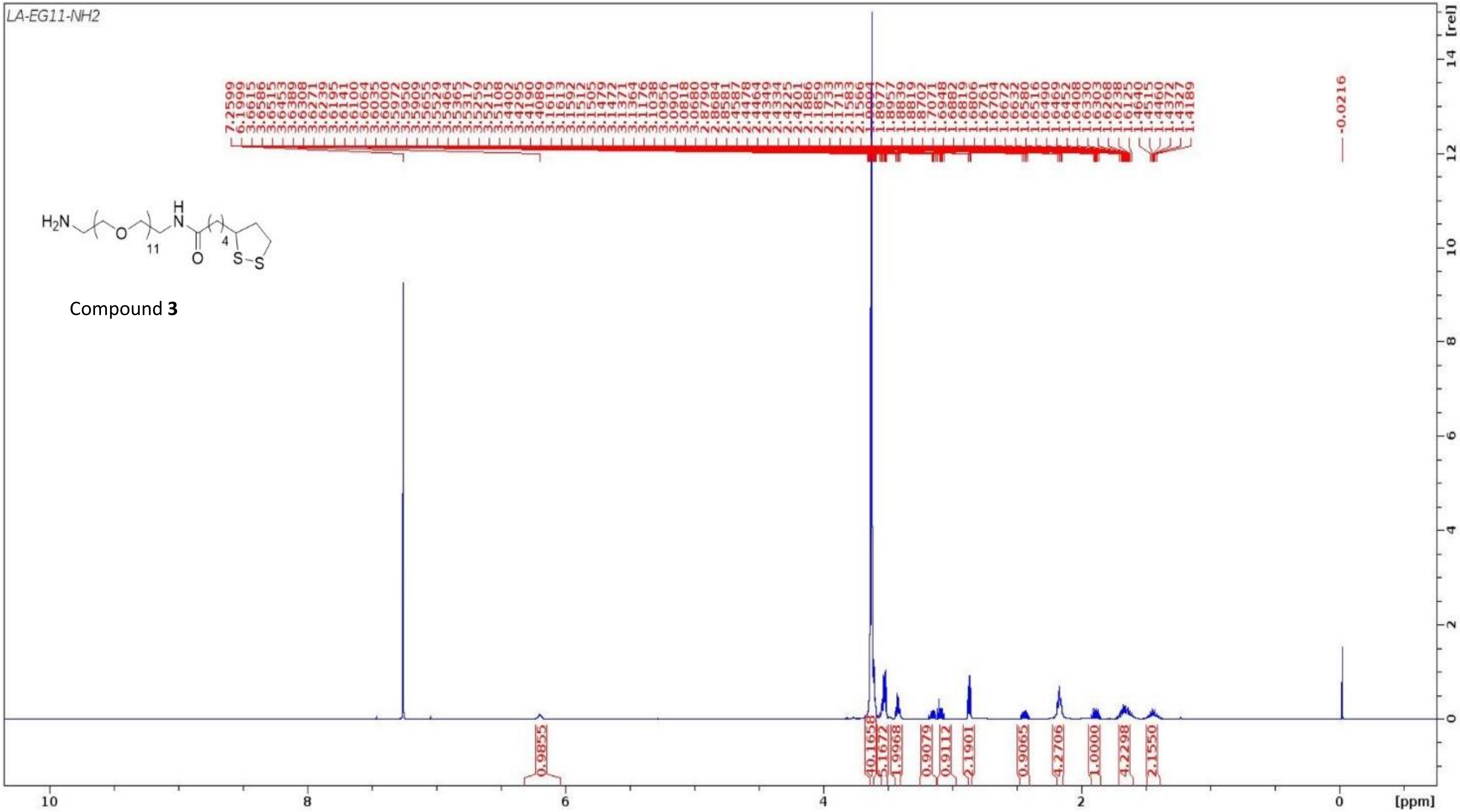

The ^1H NMR spectrum of compound **3** in $CDCl_3$

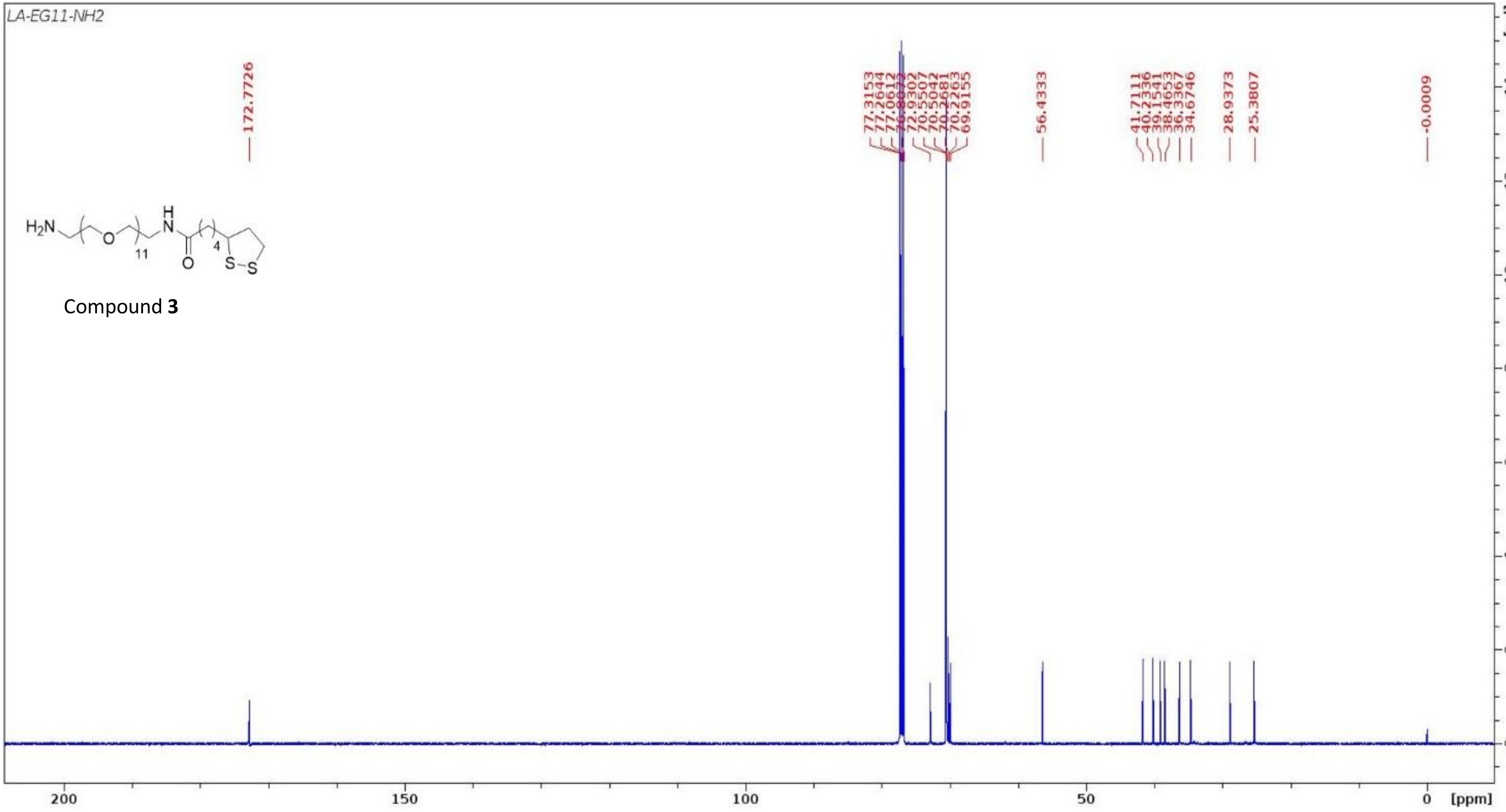

The ^{13}C NMR spectrum of compound **3** in $CDCl_3$.

LA-EG$_{11}$-MTz (compound 5):

Compound **3** (30 mg, 0.04 mmol) was dissolved in anhydrous CH_2Cl_2 (5 mL), then methyltetrazine acid, **4** (9.4 mg, 0.04 mmol) followed by DMAP (1 mg, 0.01 mmol) were added to the reaction under nitrogen at 0 ^oC. After stirring at 0 ^oC for 30 min, DCC (12.7 mg, 0.06 mmol) in dry CH_2Cl_2 (0.5 mL) was added dropwise and the reaction mixture was stirred at 0 ^oC for one hour before it was allowed to gradually warm up to room temperature and stirred overnight. After confirmation of full conversion of the starting material by TLC, the reaction mixture was filtered over celite, and the solvent was evaporated to yield the crude product, which was then purified by using flash silica gel chromatography (CH_2Cl_2: CH_3OH = 15: 1) to furnish LA-EG$_{11}$-MTz, **5** (28 mg, 72%) as a pink solid.

^{1}H NMR ($CDCl_3$, 500 MHz): δ = 8.55 (d, 2H, *J*=7.9 Hz), 7.53 (d, 2H, *J*=8.1 Hz), 6.42 (s, 1H, amide N*H*), 6.19 (s, 1H amide N*H*), 3.66 – 3.61 (m, 31H), 3.58 (s, 4H), 3.55 (ddd, 5H, *J*=6.6, 5.4, 2.6 Hz), 3.47 – 3.42 (m, 4H), 3.17 (ddd, 1H, *J*=11.6, 7.0, 5.6 Hz), 3.14 – 3.06 (m, 4H), 2.52 – 2.39 (m, 1H), 2.22 – 2.13 (m, 2H), 1.96 – 1.87 (m, 3H), 1.74 – 1.63 (m, 5H), 1.60 (dd, 1H, *J*=8.4, 3.9 Hz), 1.50 – 1.40 (m, 2H), 1.34 (ddt, 1H, *J*=12.4, 3.9, 2.2 Hz), 1.14 – 1.04 (m, 2H) ppm. ^{13}C NMR (125 MHz, $CDCl_3$) δ: 172.8, 170.1 (2 x C=O), 167.2, 163.9, 156.7, 140.2, 130.6, 130.2, 128.3, 70.6, 70.2, 69.9, 69.7, 56.4, 49.1, 40.2, 39.5, 39.2, 38.5, 36.3, 34.7, 34.0, 28.9, 25.6, 25.4, 25.0, 21.2. **HRMS:** calculated *m/z* for $C_{43}H_{72}KN_6O_{13}S_2$ $(M+K)^+$ 983.4230; found 983.4236.

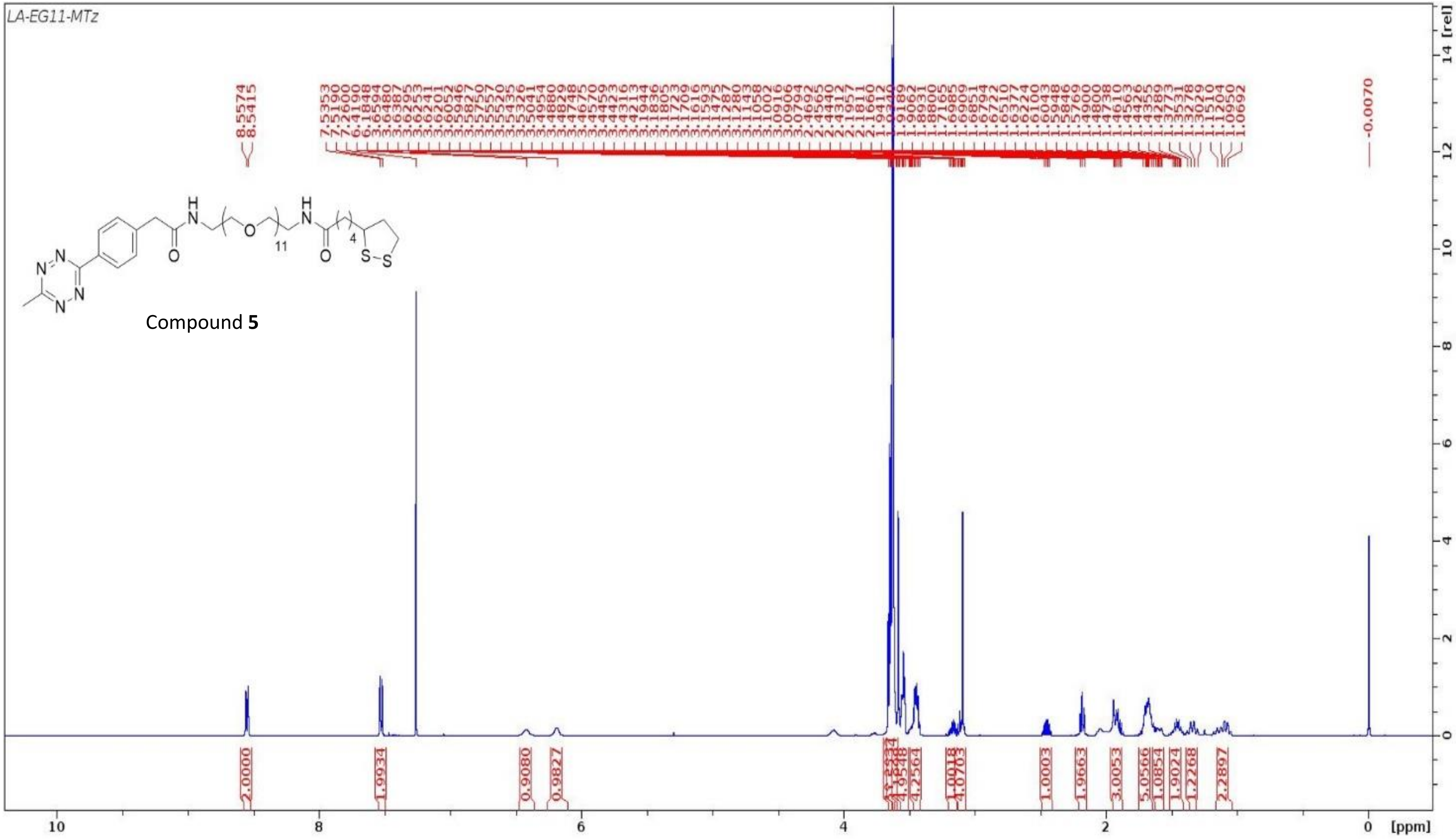

The ^{1}H NMR spectrum of compound **5** in $CDCl_3$.

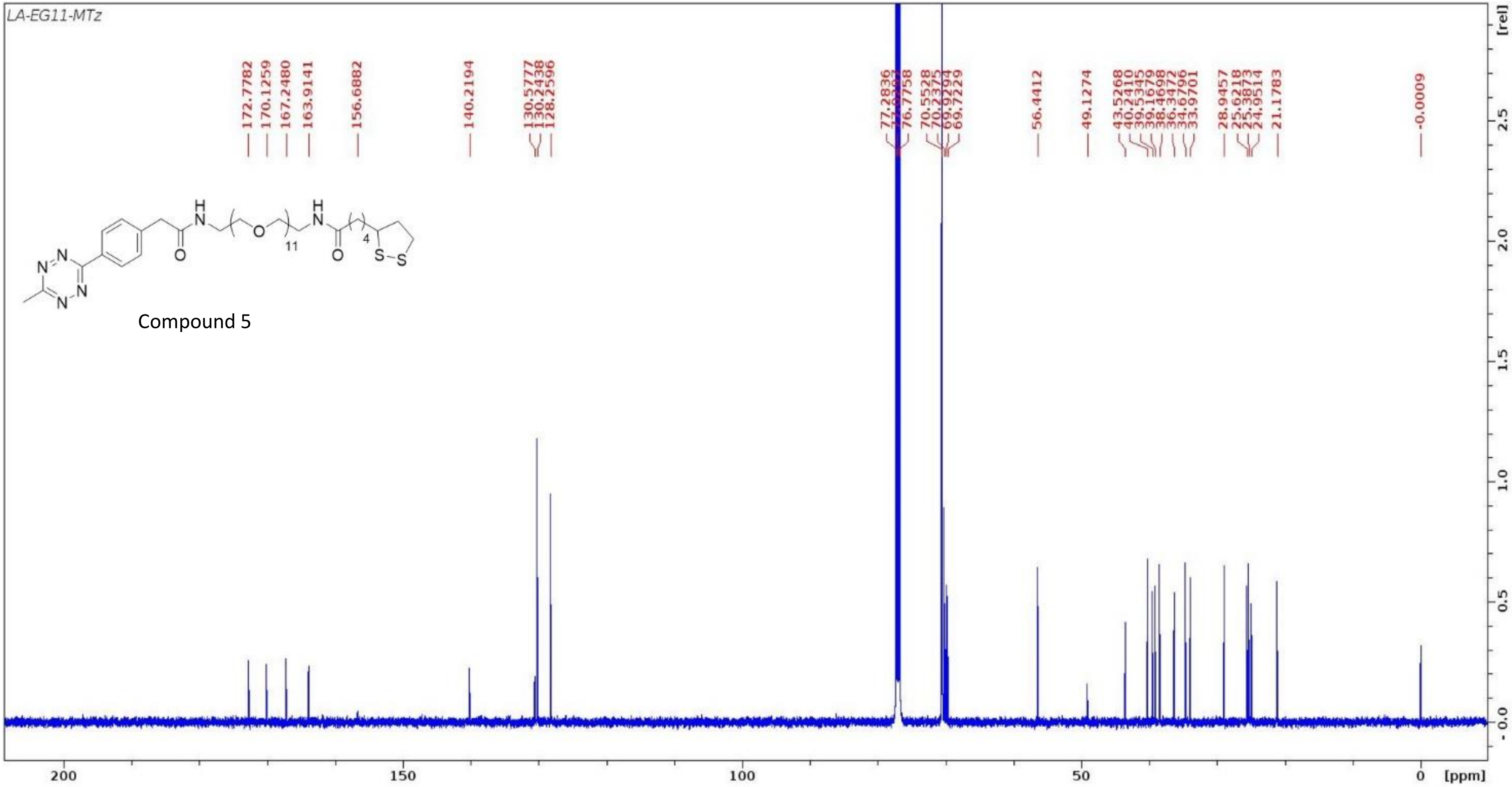

The ^{13}C NMR spectrum of compound **5** in $CDCl_3$.

LA-EG$_{11}$-Tz-TFP (compound 7):

TCO-EG$_4$-TFP, **6** (5.0 mg, 8.84 µmol) and LA-EG$_{11}$-MTz, **5** (12.3 mg, 8.84 µmol) were dissolved in anhydrous CH_2Cl_2 (400 µL) and stirred at room temperature in darkness (wrapped with aluminium foil) for 2 days till TLC ($CHCl_3$: CH_3OH = 10:1) indicated the complete conversion of the starting materials. The solvent was then evaporated, and the crude residue was purified using flash silica gel chromatography ($CHCl_3$: CH_3OH = 20:1) to yield the desired LA-EG$_{11}$-Tz-TFP linker **7** (9.0 mg, 51%) as an orange solid.

^{1}H NMR ($CDCl_3$, 500 MHz): δ = 8.57 – 8.53 (m, 2H), 7.71 – 7.68 (m, 1H), 7.55 – 7.51 (m, 2H), 7.00 (tt, 1H, *J*=9.8, 7.0 Hz), 6.44 (s, 1H), 6.21 (s, 2H), 5.14 (s, 1H), 3.90 – 3.87 (m, 1H), 3.70-3.61 (m, 53H), 3.60-3.43 (m, 17H), 3.19 – 3.16 (m, 1H), 3.13 – 3.09 (m, 4H), 2.96 – 2.93 (m, 1H), 2.45 (dtd, 2H, *J*=13.0, 6.6, 5.3 Hz), 2.19 (td, 3H, *J*=7.5, 1.4 Hz), 1.90 (q, 7H, *J*=6.9 Hz), 1.72 – 1.64 (m, 7H), 1.51-1.40 (m, 2H) ppm. ^{13}C NMR (125 MHz, $CDCl_3$) δ: 172.8(2), 170.9, 170.2 (4 x C=O) , 167.5, 167.2, 163.9, 156.4, 141.5, 140.2, 135.4, 135.3, 135.0, 134.9, 130.6, 130.2, 129.8, 124.4, 129.4, 128.3, 126.3, 126.2, 103.8, 103.4, 103.3, 103.1, 95.7, 77.2, 70.7(2), 70.6, 70.5, 70.2(2), 69.9, 69.7, 66.1, 56.4, 43.5, 43.4, 40.8, 40.2, 39.5, 39.4, 39.2, 36.3, 36.0, 34.7, 34.5, 28.9, 25.4, 21.3, 21.2, 14.5. **HRMS:** calculated *m/z* for $C_{69}H_{107}F_4N_5NaO_{21}S_2$ $(M+Na)^+$ 1504.6728; found 1504.6725.

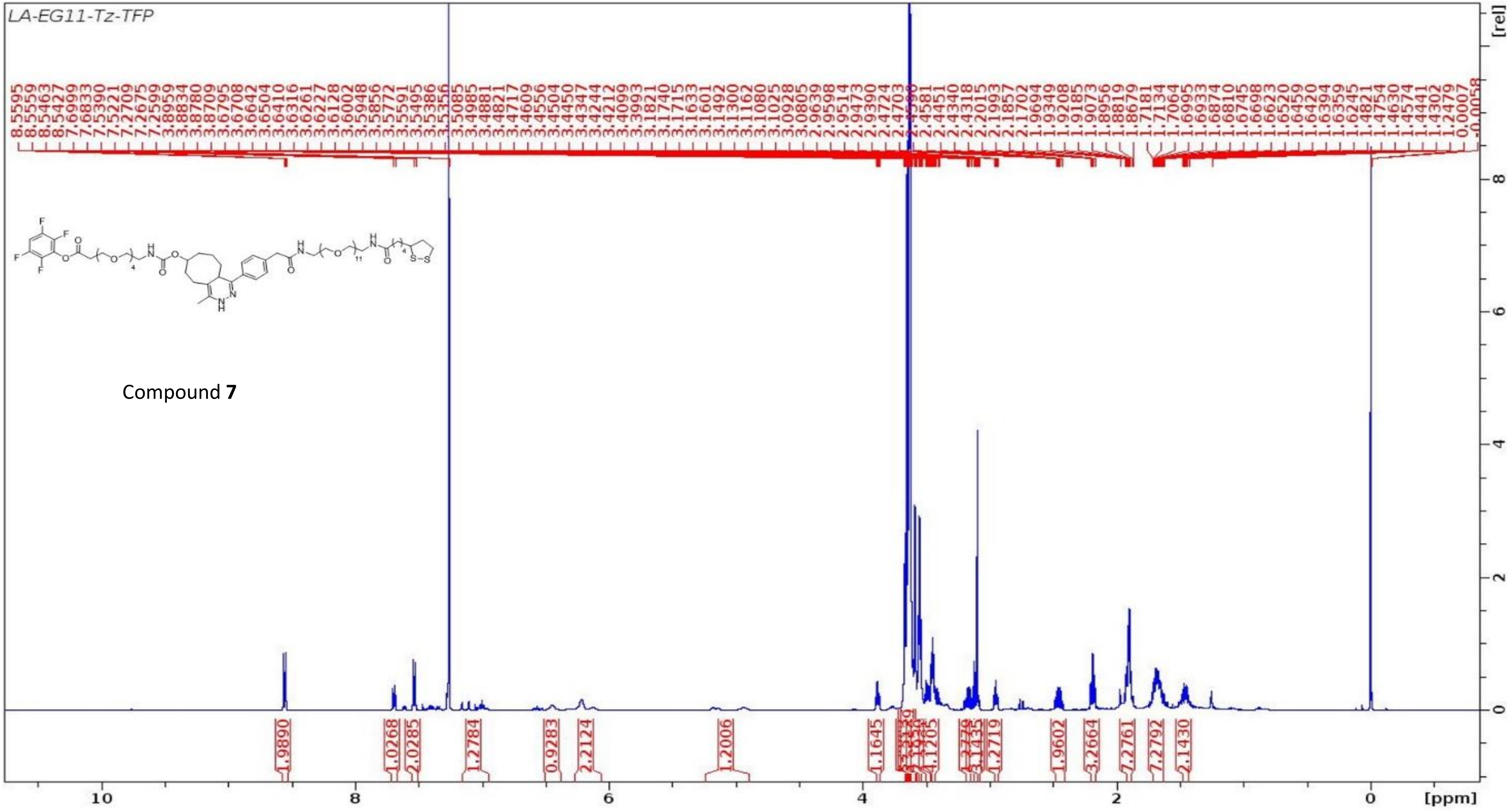

The ^{1}H NMR spectrum of compound **7** in $CDCl_3$.

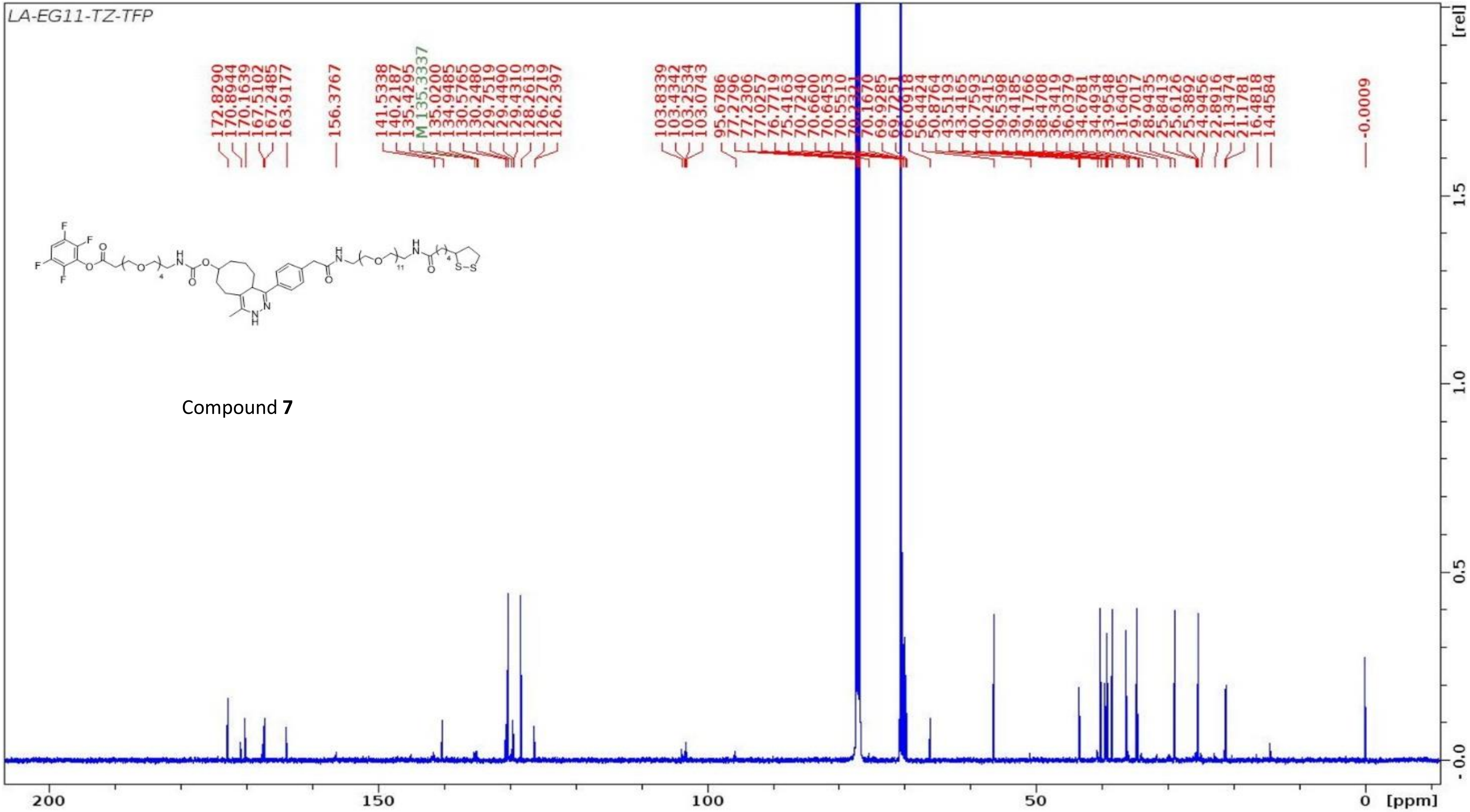

The ^{13}C NMR spectrum of compound **7** in $CDCl_3$.

3.2 Synthesis of linker 2, LA-EG$_{11}$-TFP (compound 10)

The synthetic route to the LA-EG$_{11}$-TFP linker is shown in Scheme S2 below:

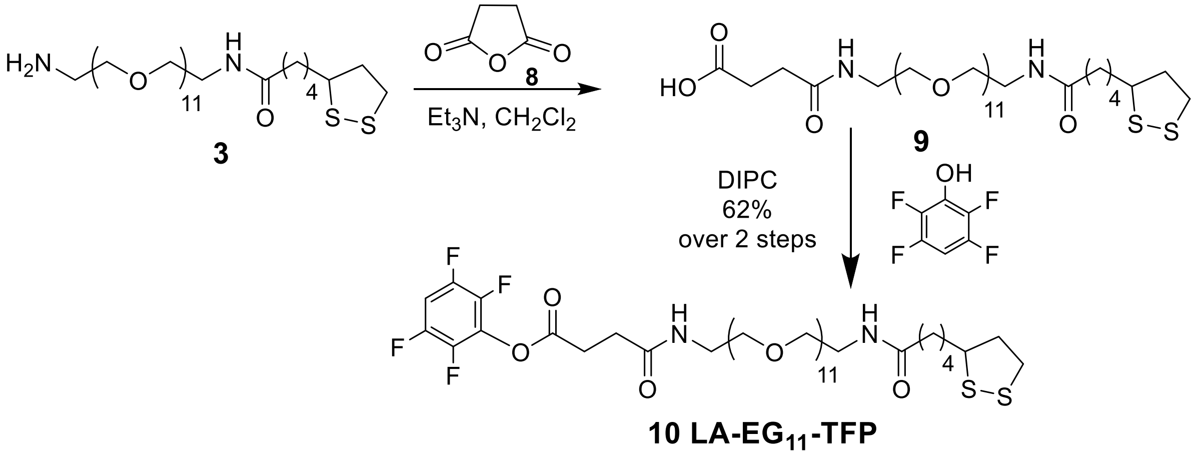

Scheme S2. Synthetic route to the LA-EG$_{11}$-TFP linker molecule.

For synthesis of compound **9** (LA-EG$_{11}$-CO_2H): triethylamine (0.36 mL, 2.58 mmol) was added dropwise to the anhydrous CH_2Cl_2 solution (20 mL) containing LA-EG$_{11}$-NH_2, **3** (757 mg, 1.03 mmol) and succinic anhydride, **8** (207 mg, 2.06 mmol) under nitrogen. After that, the reaction mixture was stirred at room temperature under nitrogen overnight. After confirmation of the completion of reaction by TLC (CH_2Cl_2: CH_3OH = 5:1), the reaction mixture was acidified with 1 M HCl (50 mL) and extracted with CH_2Cl_2 (3 x 50 mL). The organic layer was washed with brine (50 mL), dried over Na_2SO_4 and evaporated to dryness *in vacuo*. The LA-EG$_{11}$-acid compound **9** was used directly in the next step esterification reaction without purification. **HRMS:** calculated *m/z* for $C_{36}H_{68}N_2NaO_{15}S_2$ $(M+Na)^+$ 855.3953; found 855.3971.

For synthesis of compound **10**, an anhydrous THF solution (5 mL) of tetrafluorophenol (189 mg, 1.14 mmol) was added to an anhydrous THF solution (5 mL) of **9** (860 mg, 1.03 mmol) at 0 °C under nitrogen. After stirring for 5 min, *N*,*N'*-diisopropylcarbodiimide (DIPC, 0.18 mL, 1.14 mmol) was added and the reaction was further stirred at 0 °C for another hour before being allowed to warm up to room temperature gradually and then stirred overnight. After TLC (CH_2Cl_2: CH_3OH = 10:1) showed the complete conversion of the starting materials, the reaction mixture was filtered off of the insoluble *N*,*N'*-diisopropylurea byproduct, washed with THF followed by CH_2Cl_2. The organic layers were combined and evaporated *in vacuo*. The crude product was purified by automated flash chromatography using 2-10% CH_3OH in CH_2Cl_2 over 10 column volumes to furnish the LA-EG$_{11}$-TFP ester **10** (630 mg, 62 %) as a pale-yellow solid.

^{1}H NMR (500 MHz, $CDCl_3$) δ: 6.99 (tt, 1H, *J*=9.9, 7.0 Hz), 6.67 (s, 1H, amide N*H*), 6.34 (s, 1H, amide N*H*), 3.74 – 3.70 (m, 1H), 3.66 – 3.62 (m, 38H, C*H*$_2$s in EG$_{11}$ units), 3.55 (td, 4H, *J*=5.5, 4.5 Hz), 3.46 (dtd, 4H, *J*=13.1, 5.6, 4.4 Hz), 3.17 (ddd, 1H, *J*=11.0, 7.1, 5.4 Hz), 3.11 (dt, 1H, *J*=11.0, 6.9 Hz), 3.04 (t, 2H, *J*=7.1 Hz), 2.64 (t, 2H, *J*=7.1 Hz), 2.45 (dtd, 1H, *J*=13.0, 6.6, 5.4 Hz), 2.19 (td, 2H, *J*=7.5, 1.4 Hz), 1.90 (dq, 2H, *J*=12.6, 7.0 Hz), 1.73 – 1.62 (m, 4H), 1.53 – 1.40 (m, 2H), 1.25 (s, 1H) ppm; ^{13}C NMR (125 MHz, $CDCl_3$) δ: 172.9, 170.4, 169.0 (3 x C=O), 103.3, 103.1, 103.0, 72.7, 70.6, 70.5(2), 70.4, 70.2, 70.1, 69.9, 69.8, 61.6, 56.4, 40.2, 39.4, 39.2, 38.4, 36.3, 34.7, 30.4, 29.7, 28.9, 28.8, 25.4 ppm. **HRMS:** calculated *m/z* for $C_{42}H_{69}F_4N_2O_{15}S_2$ $(M+H)^+$ 981.4075; found 981.4098.

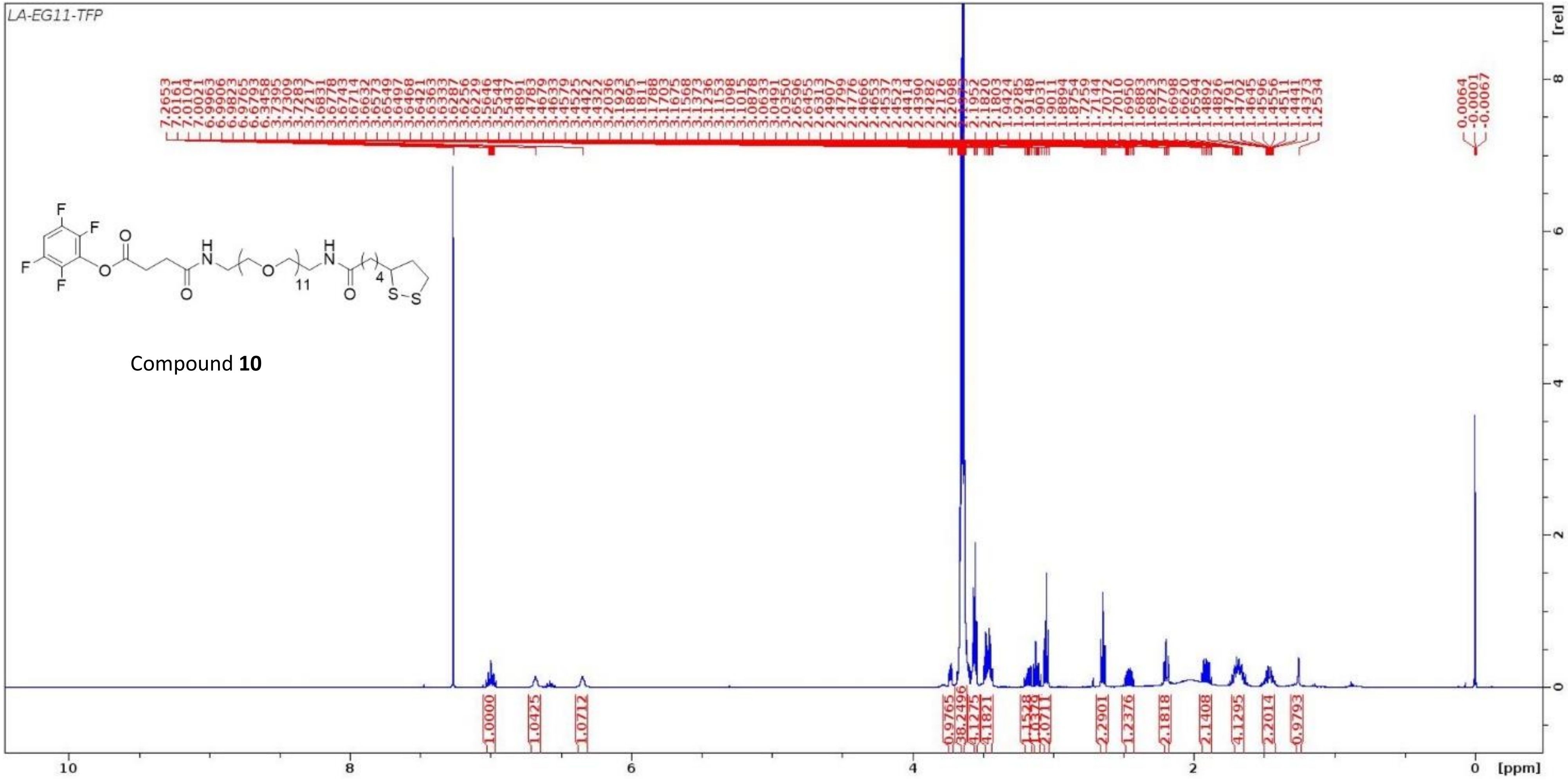

The ^{1}H NMR spectrum of compound **10** in $CDCl_3$

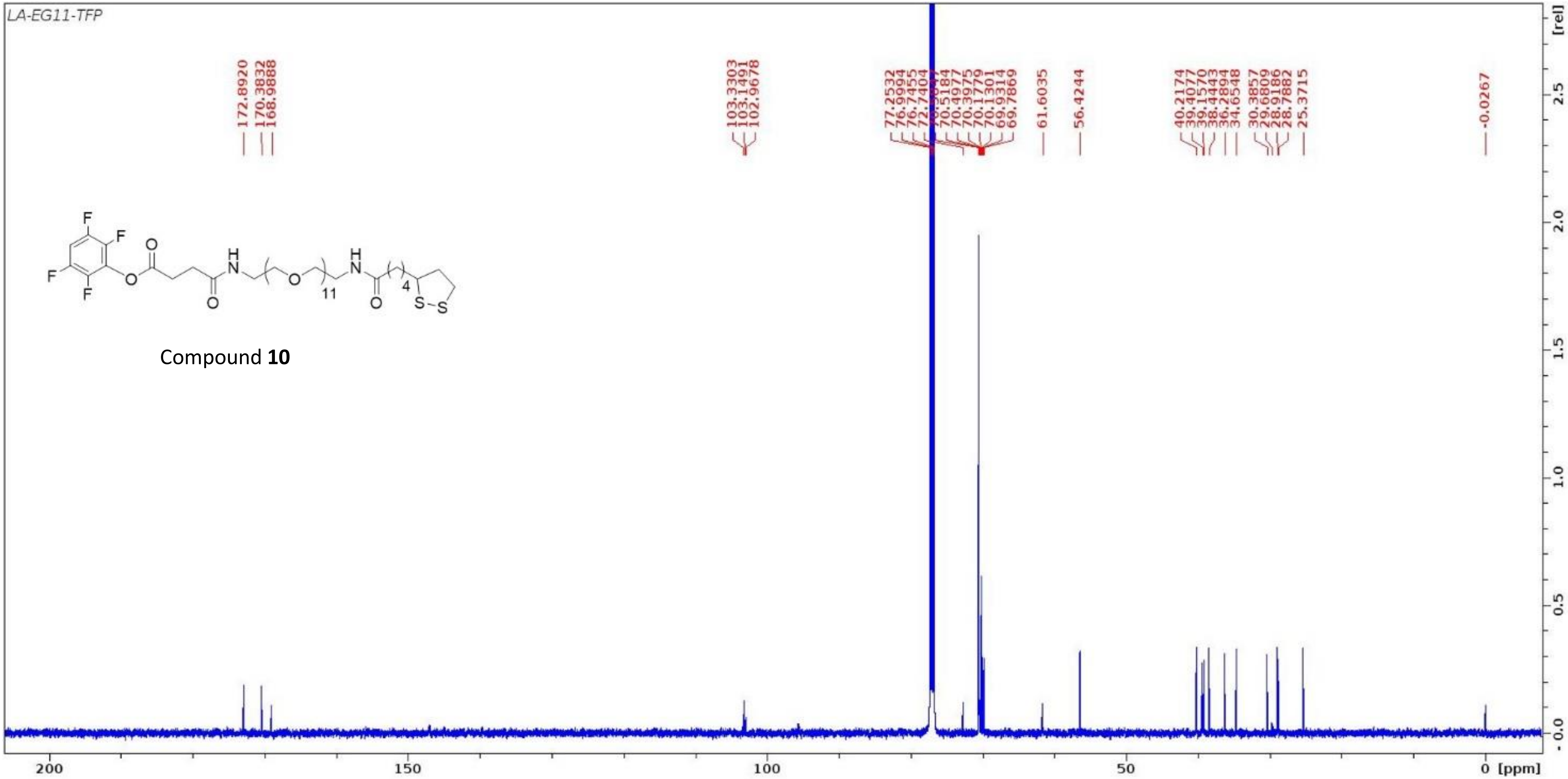

The ^{13}C NMR spectrum of compound **10** in $CDCl_3$.

3.3. Synthesis of Lipoic Acid-EG$_4$-Galactopyranoside (LA-EG$_4$-Gal) Ligand[2-4]

Synthesis of 1-Azido-3,6-dioxaoct-8-yl β-D-galactopyranoside (compound 15) [3,4]

The synthetic route is shown schematically below:

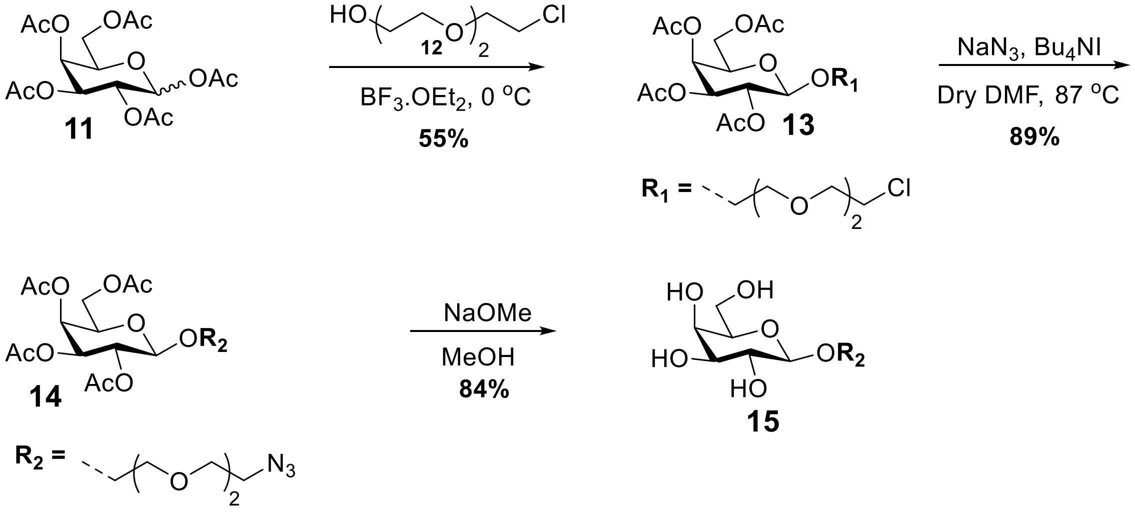

2-[2-(2-chloroethoxy)ethoxy]ethyl 2,3,4,6-tetra-O-acetyl β-D-galactopyranoside (compound 13)

2-(2-(2-Chloroethoxy)ethoxy)ethanol **12** (3.5 mL, 23 mmol) was added to a solution of D-Galactose penta-acetate **11** (4.50 g, 12 mmol) in anhydrous CH_2Cl_2 (45 mL) at 0°C under nitrogen. After stirring for 10 min at 0°C, $BF_3{\cdot}OEt_2$ (15 mL, 122 mmol) was added dropwise to the reaction mixture. The reaction was left stirring at 0 °C for one hour before being allowed to gradually warm up to room temperature and stirred overnight, when TLC (EtOAc: *n*-hexane = 2:1) indicated the full consumption of the starting materials. The reaction mixture was then sequentially washed with water (30 mL), $NaHCO_3$ (2 x 30 mL). The organic layers were combined, dried over Na_2SO_4, and then rotary evaporated to dryness. The crude product was purified by automated silica gel flash chromatography using 10-70% EtOAc in *n*-hexane over 10 column volumes to furnish the desired product **13** (3.16g, 55%) as a yellow oil.

^{1}H NMR ($CDCl_3$, 400 MHz): δ = 5.38 (dd, 1H, *J*=3.5, 1.1 Hz, H-4), 5.20 (dd, 1H, *J*=10.5, 8.0 Hz, H-2), 5.01 (dd, 1H, *J*=10.5, 3.4 Hz, H-3), 4.57 (d, 1H, *J*=8.0 Hz, H-1), 4.19 – 4.09 (m, 2H), 3.99 – 3.89 (m, 2H, H-5, H-6a), 3.76 – 3.73 (m, 4H, H-6b), 3.69 (s, 2H), 3.65 (td, 5H, *J*=2.8, 1.7 Hz), 2.14 (s, 3H), 2.05 (s, 3H), 2.04 (s, 3H), 1.98 (s, 3H) (4 x CH_3 of OAc groups) ppm. ^{13}C NMR (101 MHz, $CDCl_3$): δ (ppm) 170.4, 170.2, 170.1, 169.5 (4xC=O), 101.4 (C-1), 72.4, 71.4, 70.9, 70.7, 70.4, 69.1, 68.8, 67.1, 61.8, 61.3, 42.7, 20.8, 20.7(2), 20.6 (4×C(O)$\underline{C}H_3$). **HRMS** (*m/z)*: Calculated for $C_{20}H_{32}ClO_{12}$ $(M+H)^+$ 499.1582, found 498.1561.

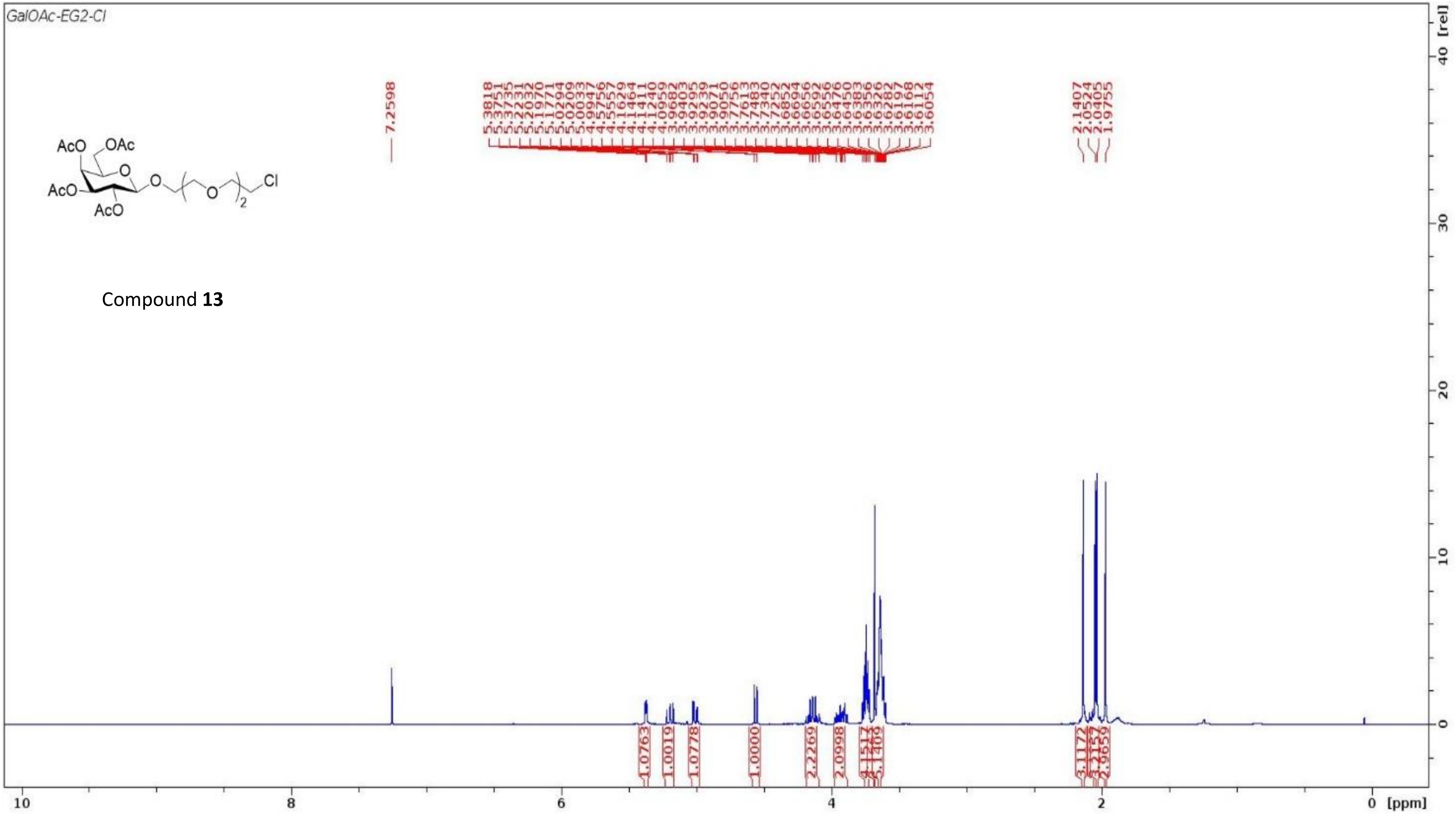

Compound **13**

The ^{1}H NMR spectrum of compound **13** in $CDCl_3$

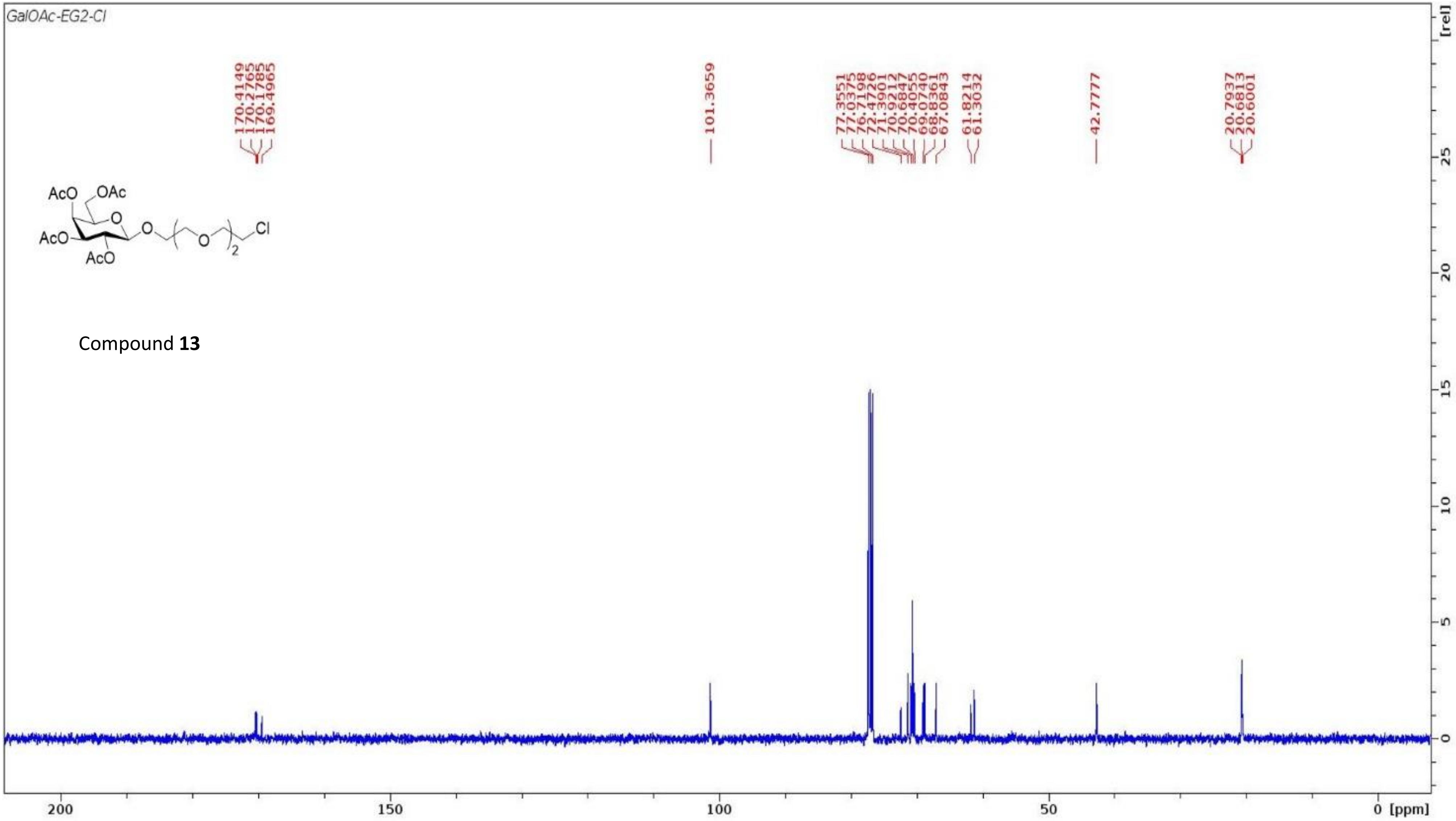

The ^{13}C NMR spectrum of compound **13** in $CDCl_3$.

1-Azido-3,6-dioxaoct-8-yl 2,3,4,6-tetra-O-acetyl β-D-galactopyranoside (Compound 14)

To a stirred solution of compound **13** (1.01g, 2 mmol) in anhydrous DMF (30 mL) under nitrogen was added NaN_3 (0.66 g, 10 mmol) followed by Bu_4NI (0.75 g, 2 mmol), the reaction mixture was then heated at 87 °C overnight. The reaction was stopped when TLC (EtOAc: *n*-hexane = 2:1) confirmed the complete conversion of the starting materials. The solvent was evaporated under reduced pressure, and the residue was dissolved in DCM washed with water (50 mL) and brine (50 mL) sequentially, dried over Na_2SO_4 and evaporated to dryness. The crude product was purified by automated flash chromatography using 20-80% EtOAc in *n*-hexane over 10 column volumes to furnish the desired product **14** (0.91 g, 89%) as a pale-yellow oil.

^1H NMR ($CDCl_3$, 400 MHz): δ = 5.32 (dd, 1H, *J*=3.5, 1.1 Hz, H-4), 5.13 (dd, 1H, *J*=10.5, 7.9 Hz, H-2), 4.95 (dd, 1H, *J*=10.5, 3.4 Hz, H-3), 4.51 (d, 1H, *J*=7.9 Hz, H-1), 4.14 – 4.03 (m, 2H), 3.93 – 3.83 (m, 2H), 3.69 (ddd, 1H, *J*=11.0, 6.8, 4.0 Hz), 3.62 – 3.59 (m, 4H), 3.58 (s, 4H), 3.33 (t, 2H, *J*=5.0 Hz), 2.08 (s, 3H), 1.99 (s, 3H), 1.98 (s, 3H), 1.91 (s, 3H) (4 x CH_3 of OAc groups) ppm. ^{13}C NMR ($CDCl_3$, 100 MHz): δ = 170.3, 170.2, 170.1, 169.4 (4xC=O), 101.3 (C-1), 70.9, 70.7, 70.6 (2), 70.4, 70.0, 69.0, 68.8, 67.1, 61.3, 50.2, 20.7, 20.6(2), 20.5 (4×C(O)$\underline{C}H_3$) ppm. **HRMS** (*m/z)*: Calculated for $C_{20}H_{33}N_3O_{12}$ $(M+H)^+$ 506.1986, found 506.1992.

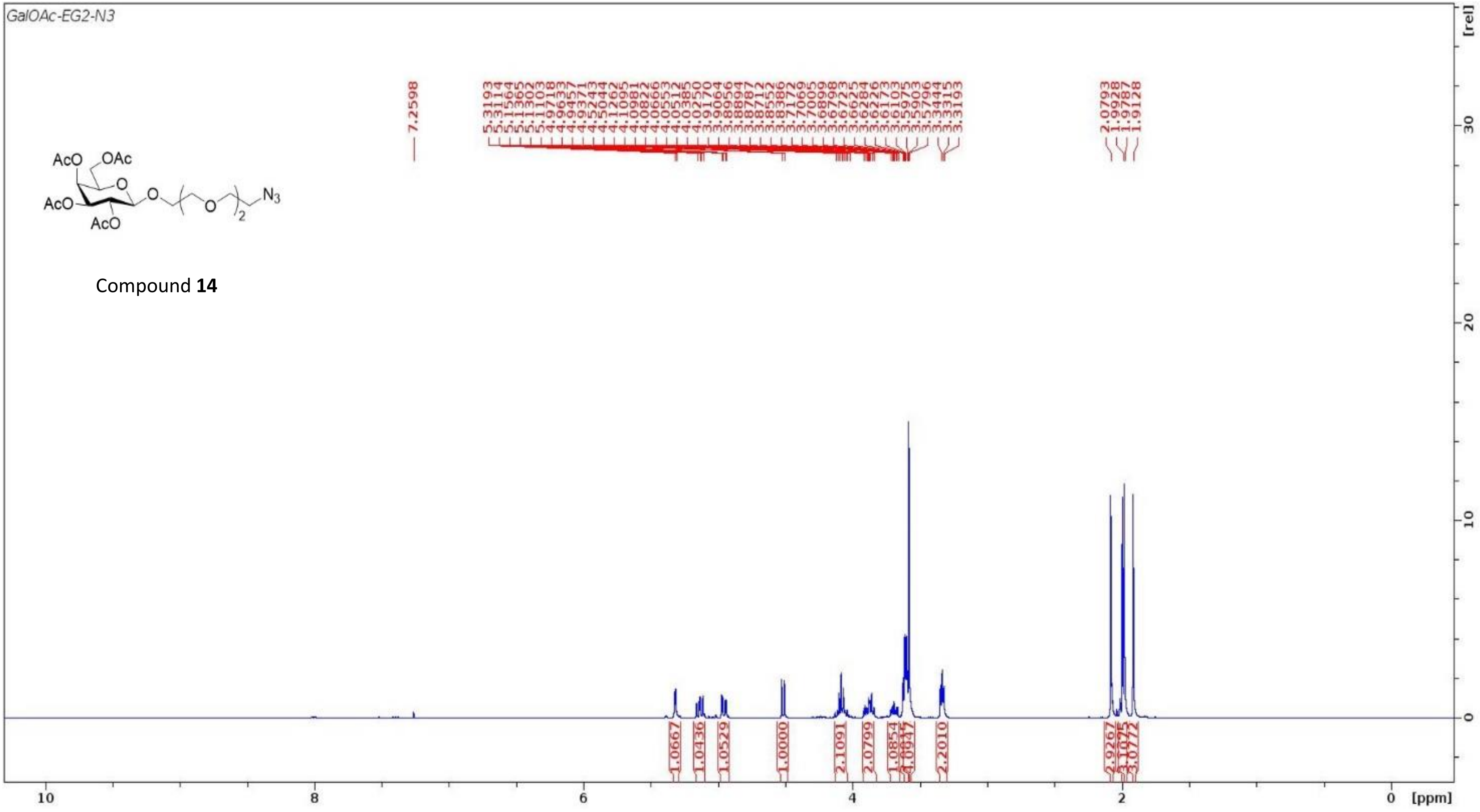

Compound **14**

The ^{1}H NMR spectrum of compound **14** in $CDCl_3$.

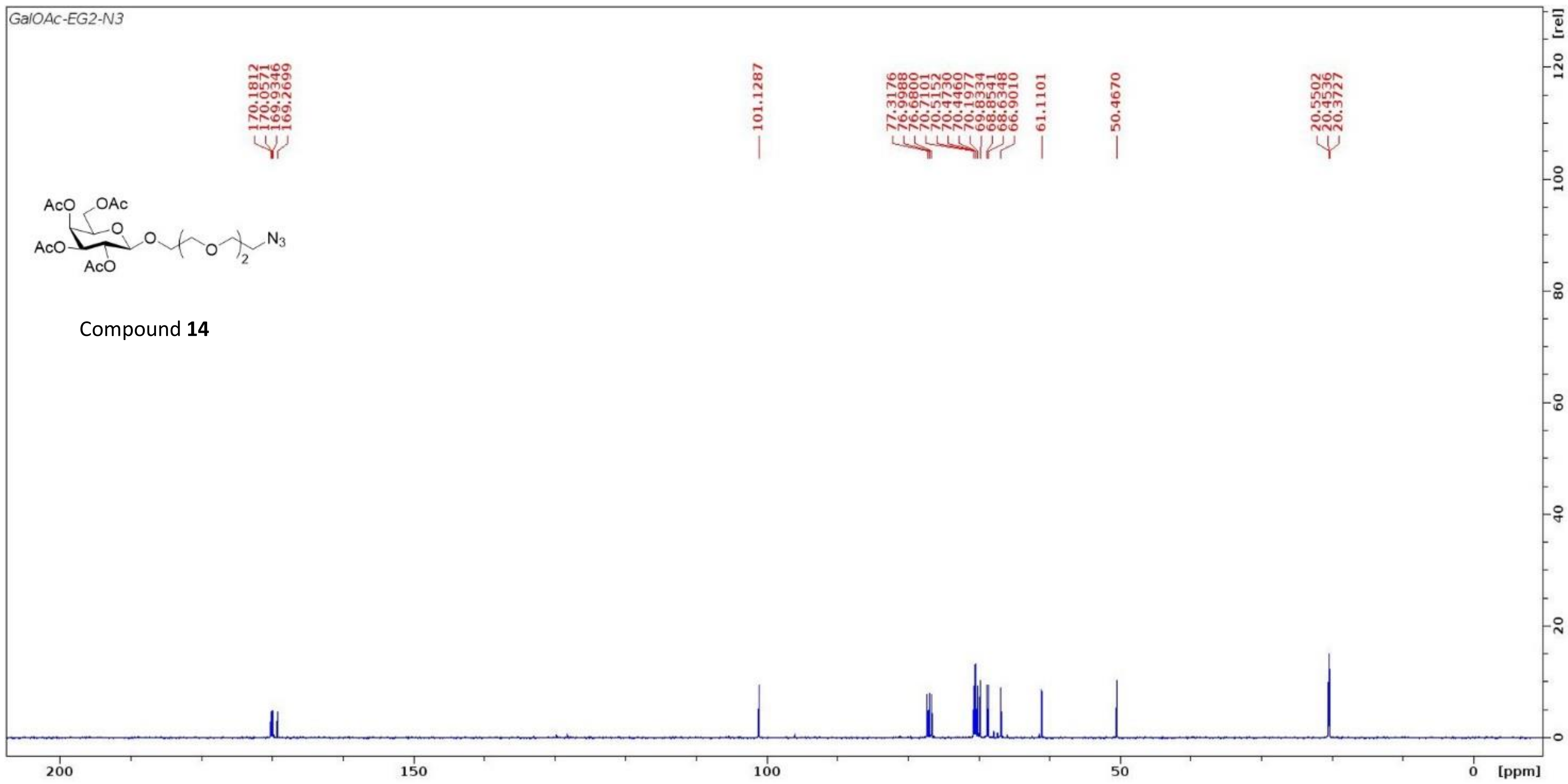

Compound **14**

The ^{13}C NMR spectrum of compound **14** in $CDCl_3$.

1-Azido-3,6-dioxaoct-8-yl β-D-galactopyranoside (compound 15)[2-4]

Compound **14** (0.48 g, 0.95 mmol) was dissolved in anhydrous CH_3OH (15 mL) and NaOMe (0.5 M in CH_3OH, 1.0 mL) was gradually added to the reaction mixture under nitrogen. The reaction mixture was stirred at room temperature for 3 hours till TLC ($CHCl_3$: CH_3OH = 5:1) indicated the complete conversion of the starting material to a more polar compound. Then Amberlite H^+ resins were added in small portions to acidify the reaction mixture to neutral pH. The protonated product was then filtered off to get rid of the resin, and the solvent was evaporated *in vacuo*. The crude product was purified by automated silica gel flash chromatography using 40-80% CH_3OH in CH_2Cl_2 over 10 column volumes to give the desired product **15** (0.27 g, 84% yield) as a colourless oil.

^{1}H NMR (D_2O, 400 MHz): δ = 4.35 (d, 1H, *J*=7.2 Hz, H-1), 4.10 (m, 1H), 3.93 (d, 1H, *J*=3.1 Hz), 3.86 – 3.79 (m, 5H), 3.77-3.74 (m, 6H), 3.64-3.55 (m, 3H), 3.48 (t, 2H, *J*=4.9 Hz) ppm. ^{13}C NMR (CD_3OD, 100 MHz): δ = 104.9 (C-1), 76.5, 74.7, 72.4 (2), 71.3, 70.9, 70.2, 70.1, 69.5, 62.3, 51.6, 49.6-48.4 (CD_3OD solvent) ppm. **HRMS** (*m/z)*: Calculated for $C_{12}H_{23}N_3NaO_8$ $(M+Na)^+$ 360.1383, found 360.1378.

GalOH-EG2-N3

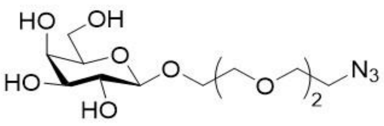

Compound **15**

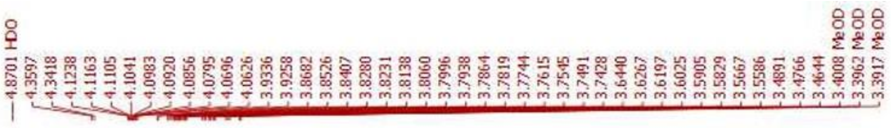

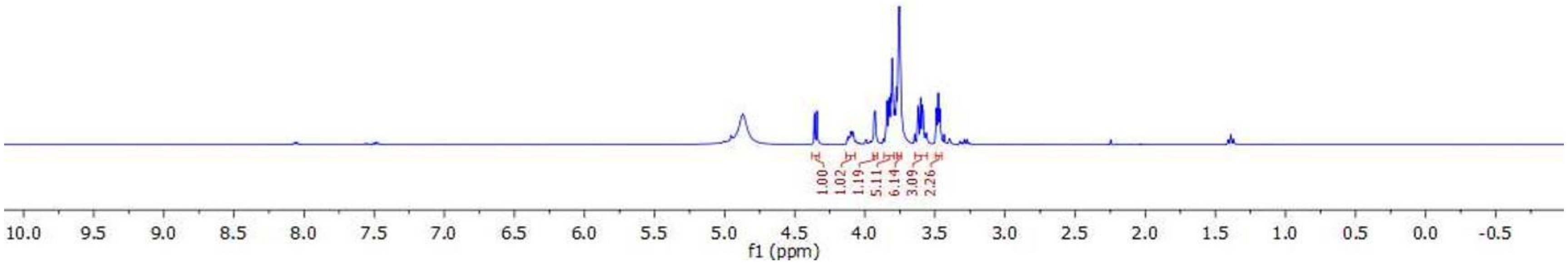

The ^{1}H NMR spectrum of compound **15** in D_2O

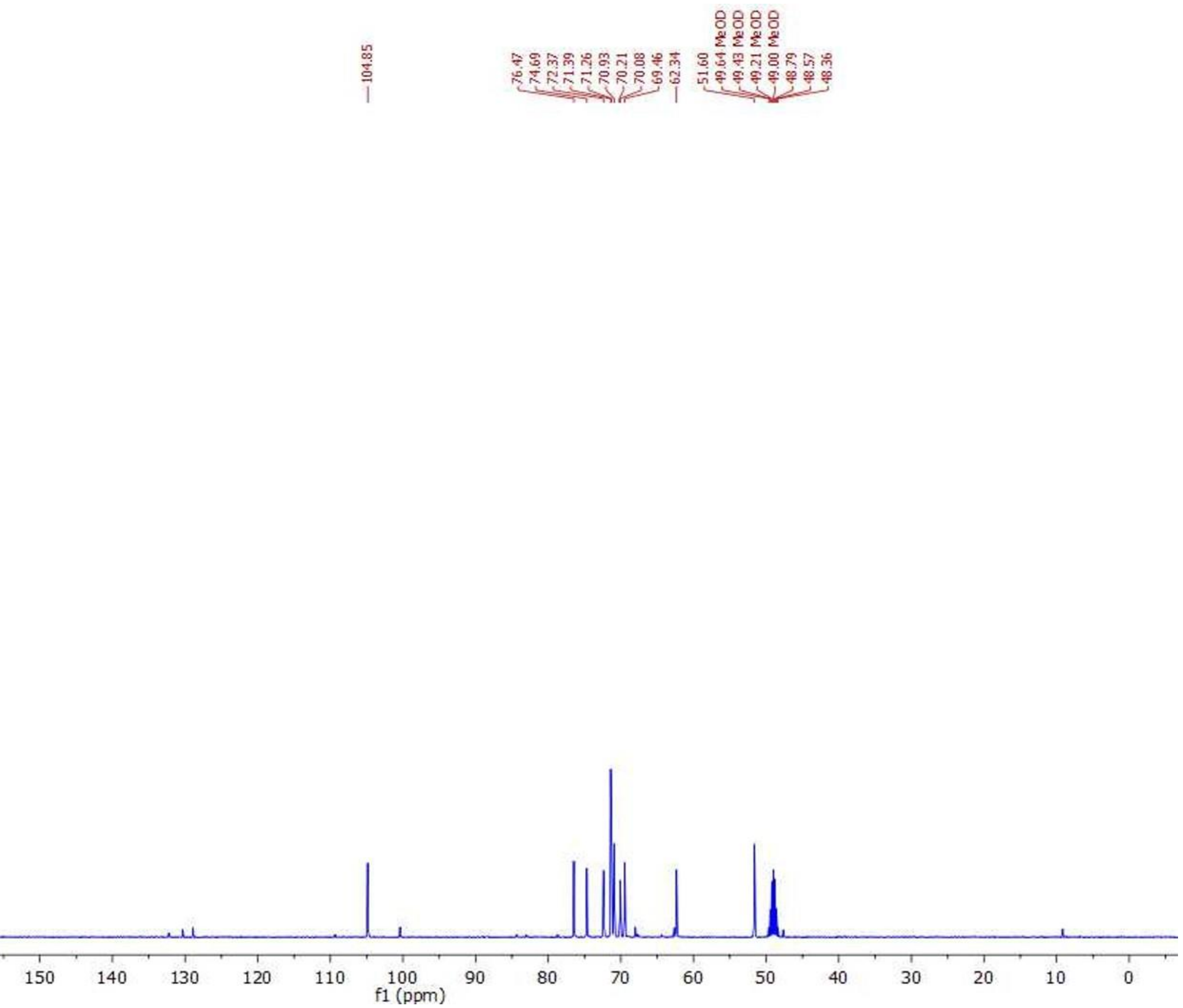

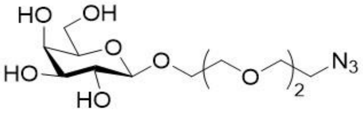

Compound **15**

The ^{13}C NMR spectrum of compound **15** in CD_3OD.

Synthesis of LA-EG_4-C≡CH (compound 16)[2]

To the anhydrous CH_2Cl_2 solution (25 mL) of H_2N-EG_4-C≡CH (2.00g, 8.65 mmol), lipoic acid, **4** (1.80 g, 8.65 mmol) followed by DMAP (0.21g, 1.73 mmol) were added and the reaction mixture was stirred at 0 °C under nitrogen for 15 min. After that, DCC (1.82g, 10.2 mmol) dissolved in anhydrous CH_2Cl_2 (5 mL) was added dropwise to the reaction mixture over 20 min and the resulting reaction was stirred at 0 °C for an hour before being gradually allowed to warm to room temperature and then stirred overnight. Upon completion, the reaction mixture was filtered over celite to remove the DCU byproduct and the solvent evaporated. The crude product was purified by flash silica chromatography ($CHCl_3$: CH_3OH = 10:1) to furnish the desired product, **16**, as a yellowish oil (3.08g, yield 85%).

^{1}H NMR ($CDCl_3$, 500 MHz): δ = 6.50 (t, 1H, *J*=5.7 Hz, amide N*H*), 4.00 (d, 2H, *J*=2.4 Hz), 3.52-3.45 (m, 8H), 3.45-3.30 (m, 7H), 3.24 (td, 2H, *J*=5.6, 4.5 Hz), 3.05 – 2.90 (m, 2H), 2.30 (s, 1H), 2.28 – 2.20 (m, 1H), 2.01 (t, 2H, *J*=7.5), 1.75 – 1.65 (m, 1H), 1.60 – 1.40 (m, 4H), 1.32 – 1.18 (m, 2H) ppm. ^{13}C NMR ($CDCl_3$, 125 MHz): δ 172.4 (C=O), 79.0, 74.4, 69.9(2), 69.8, 69.7, 69.5, 69.2, 68.4, 57.7, 55.8, 39.6, 38.6, 37.9, 35.6, 34.1, 28.3, 24.8 ppm. **HRMS**: calculated *m/z* for $C_{19}H_{34}NO_5S_2$ $(M+H)^+$ 420.1878; found 420.1873.

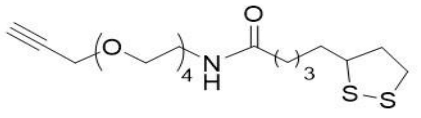

Compound **16**

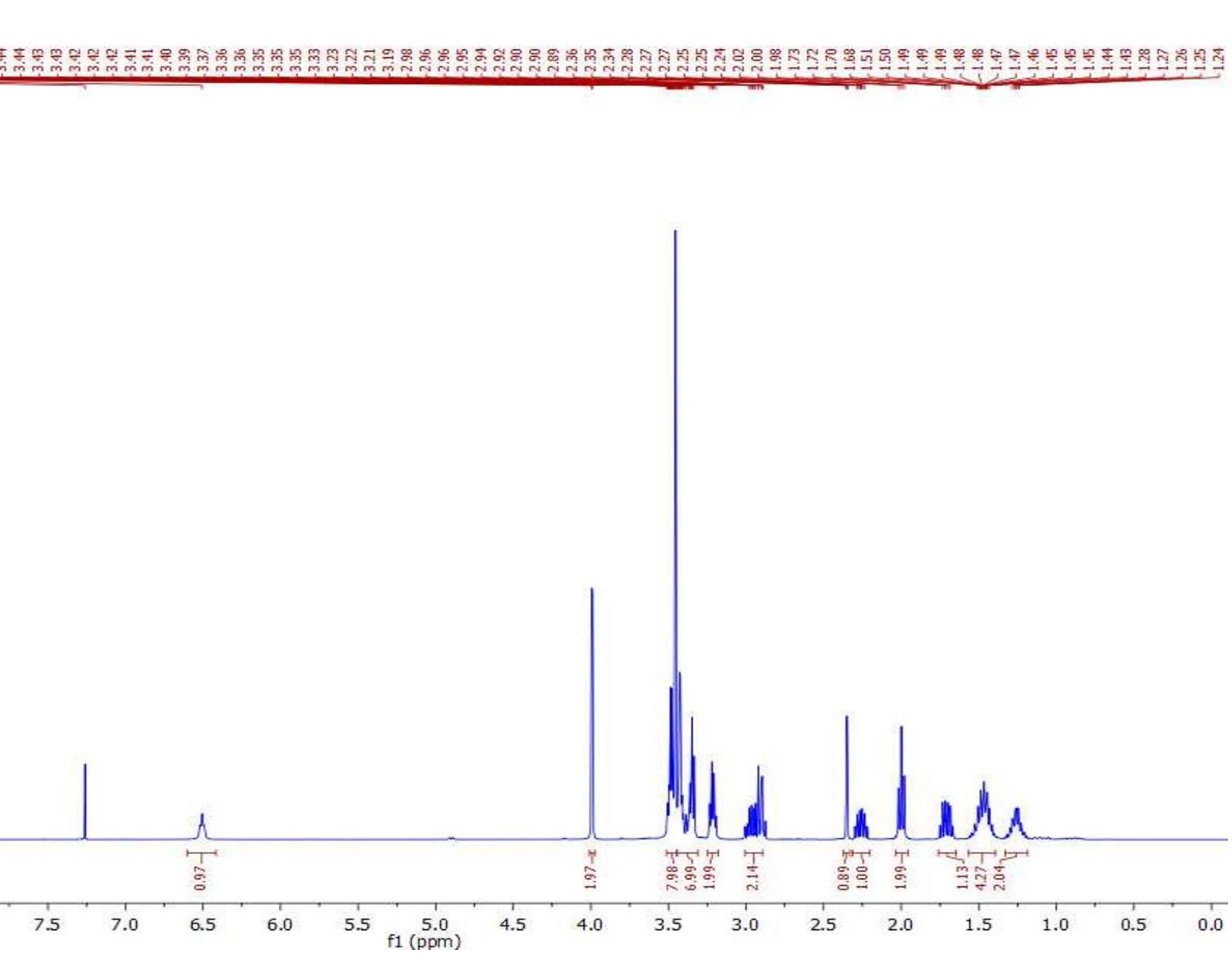

The ^1H NMR spectrum of compound **16** in $CDCl_3$

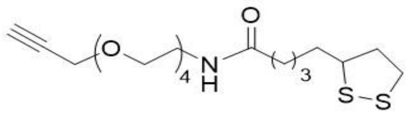

Compound **16**

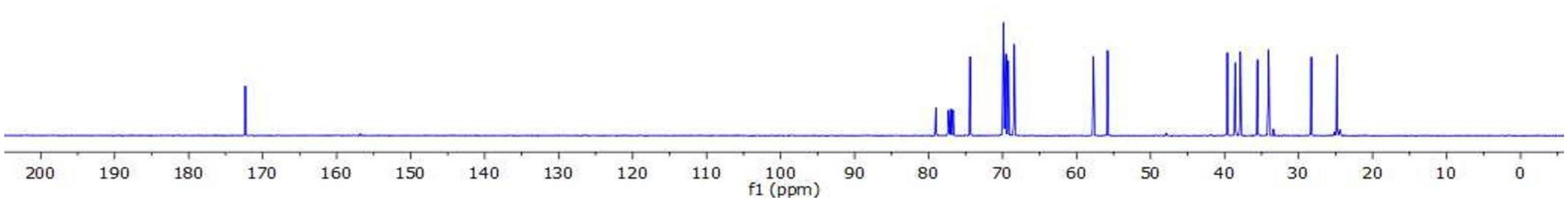

The ^{13}C NMR spectrum of compound **16** in $CDCl_3$.

Synthesis of the LA-EG$_4$-glycan ligands *via* click chemistry

All LA-EG$_4$-glycans (glycan = Gal, DiMan) were synthesised using the conventional Cu-catalyzed click chemistry as shown schematically below.[2,3]

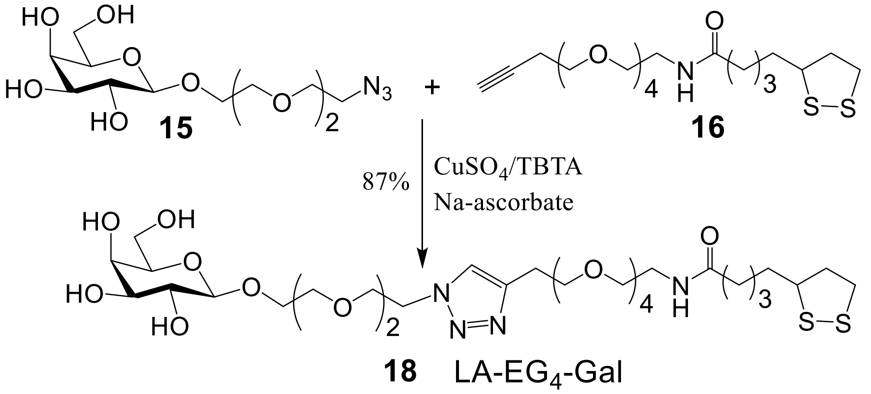

Compound **18**: to a 1:1 (v:v) mixed THF: H_2O solution (3.0 mL) containing compound **15** (40 mg, 119 μmol) and LA-EG$_4$-C≡CH (compound **16**, 45 mg, 108 μmol) was added $CuSO_4.5H_2O$ (1.0 mg, 4.0 μmol), Tris[(1-benzyl-1H-1,2,3-triazol-4-yl)methyl]amine (TBTA, 3.6 mg, 6.8 μmol), followed by sodium ascorbate (2.9 mg, 14.6 μmol), and the resulting solution was stirred at room temperature. After 3 h, TLC confirmed the complete consumption of all starting materials. The solvent was then evaporated, and the crude product was purified by size exclusion chromatography using a Biogel P2 column using water as eluent to afford the desired product, compound **18**, in 87% yield (78 mg).

Compound **18**: ^{1}H NMR (D_2O, 500 MHz): δ = 8.11 (s, 1H, triazole-H), 4.70 (s, 2H), 4.65 (t, 2H, *J*=5.1 Hz), 4.40 (d, 1H, *J*=7.9 Hz, H-1), 3.99 (t, 2H, *J*=5.0 Hz), 3.92 (d, 1H, *J*=3.4 Hz), 3.82 – 3.58 (m, 28H, CH$_2$s in EG$_n$ units), 3.56 – 3.50 (m, 2H), 3.38 (t, 2H, *J*=5.3 Hz), 3.26 – 3.15 (m, 2H), 2.48 (dq, 1H, *J*=12.2, 6.0 Hz), 2.25 (t, 2H, *J*=7.3 Hz), 2.00 – 1.94 (m, 1H), 1.78 – 1.69 (m, 1H), 1.66-1.60 (m, 3H), 1.44-1.38 (p, 2H, *J*=7.6 Hz) ppm. ^{13}C NMR (D_2O, 125 MHz): δ = 176.9 (C=O), 102.8 (C-1), 75.1, 72.7, 70.7, 69.7 (2), 69.6 (3), 69.5 (2), 69.4, 69.2, 68.9, 68.8, 68.7, 68.6 (2), 63.1, 60.9, 56.5, 50.1, 50.0, 40.2, 38.9, 38.0, 35.4, 33.7, 27.8, 25.0 ppm. **HRMS**: Expected $C_{31}H_{56}N_4O_{13}S_2$ *m/z* 757.3319, found 757.3380.

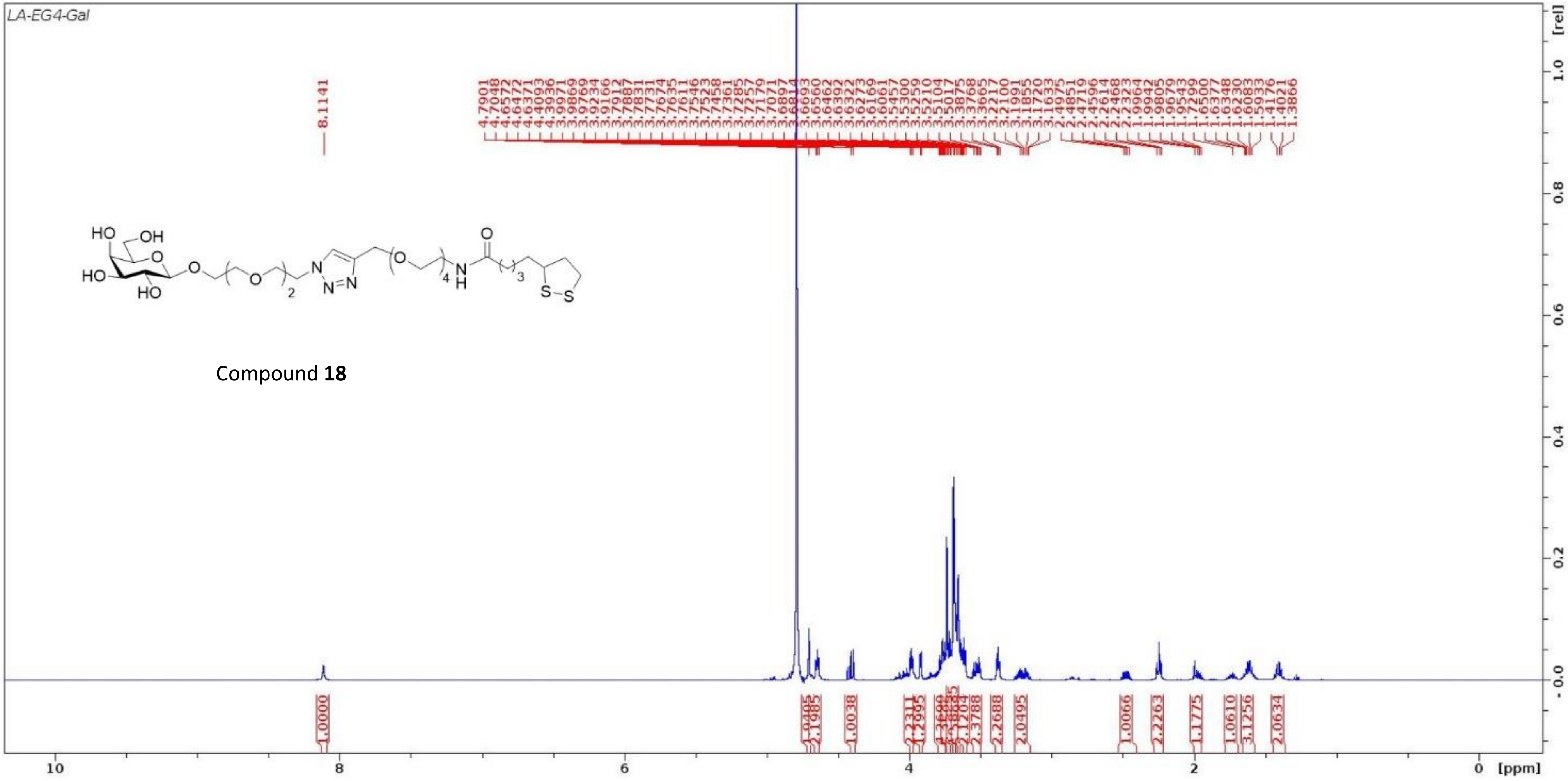

The ^{1}H NMR spectrum of compound **18** in D_2O

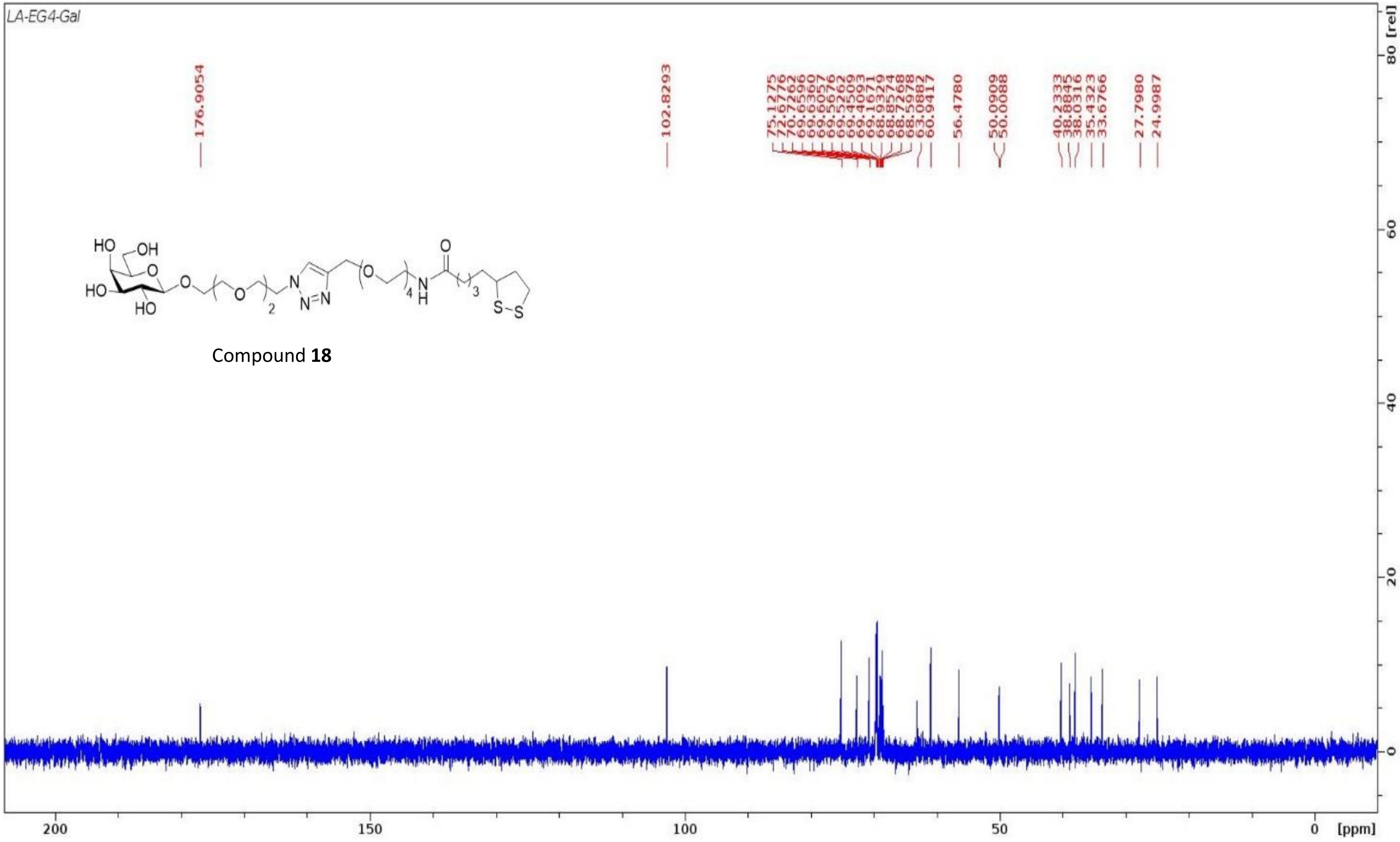

The ^{13}C NMR spectrum of compound **18** in D_2O.

3.4 Synthesis of LA-EG_4-DiMan (compound 20).[2-4]

Compound **19** (DiMan-EG_2-N_3) was synthesized *via* our established protocols as reported previously.[1,2] Its ^1H and ^{13}C NMR and proton-coupled HSQC spectra are shown in the following slides. ^1H NMR (D_2O, 500 MHz): δ = 5.11 (d, 1H, *J* = 1.6 Hz, H-1'), 4.96 (d, 1H, *J* = 1.7 Hz, H-1), 3.98 (dd, 1H, *J* = 3.4, 1.8 Hz), 3.84 (m, 5H), 3.71 – 3.64 (m, 13H), 3.58 (dd, 2H, *J* = 9.3, 3.4 Hz), 3.38 (t, 2H, *J* = 4.9 Hz). ^{13}C NMR (CD_3OD, 125 MHz): δ 104.3 (C-1), 102.2 (C-1'), 80.7, 75.1, 74.7, 72.5, 72.2, 72.0, 71.8, 71.6, 71.5, 71.3, 69.1, 68.9, 68.0, 63.3, 63.2, 51.9 ppm. Proton-coupled HSQC NMR spectrum gives the C1-H1 distance of ~170 Hz, confirming they are α-linkages. **HRMS**: calculated *m/z* for $C_{18}H_{33}N_3NaO_{13}$ $(M+Na)^+$ 522.1911; found 522.1935.

Compound **20** (LA-EG_4-DiMan) was synthesized *via* the Cu-catalyzed click chemistry as shown schematically below.[2-4] Briefly, to a 1:1 (v:v) mixed THF: H_2O solution (3.0 mL) containing compound **19** (50 mg, 100 μmol) and LA-EG_4-C≡CH (compound **16**, 38 mg, 91 μmol) was added $CuSO_4.5H_2O$ (0.84 mg, 3.4 μmol), Tris[(1-benzyl-1H-1,2,3-triazol-4-yl)methyl]amine (TBTA, 3.0 mg, 5.7 μmol), followed by sodium ascorbate (2.4 mg, 12.3 μmol), and the resulting solution was stirred at room temperature. After 3 h, TLC confirmed the complete consumption of all starting materials. The solvent was then evaporated, and the crude product was purified by size exclusion chromatography using a Biogel P2 column using water as eluent to afford the desired product, **20**, in 72% yield (66 mg).

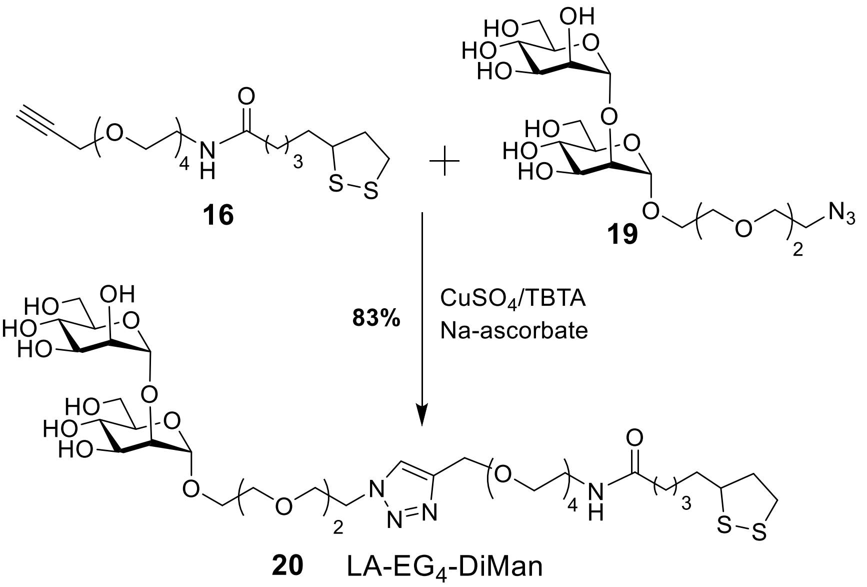

Compound **20** (LA-EG_4-DiMan): ^1H NMR (D_2O, 400 MHz): δ = 8.11 (s, 1H, triazole-H), 5.12 (s, 1H, Man H-1'), 5.03 (s, 1H, Man H-1), 4.71 (s, 2H), 4.66 (t, 2H, *J* = 5.1 Hz), 4.08 (d, 1H, *J* = 3.1 Hz), 3.99 (m, 3H), 3.95 – 3.83 (m, 5H), 3.82 – 3.60 (m, 28H, CH_2s in EG_n units), 3.40 (t, 2H, *J* = 5.3 Hz), 3.27-3.15 (m, 2H), 2.49 (m, 1H), 2.26 (t, 2H, *J* = 7.3 Hz), 1.98 (m, 1H), 1.75 (m, 1H), 1.64 (dd, 3H, *J* = 14.0, 7.6 Hz), 1.41 (p, 2H, *J* = 7.7 Hz) ppm. ^{13}C NMR (D_2O, 100 MHz): δ = 176.9 (C=O), 143.9 (<u>C</u>=CH), 125.5 (C=<u>C</u>H), 102.3 (Man C-1), 98.4 (Man-C-1'), 78.6, 73.3, 72.7, 70.3, 70.2, 69.9, 69.6, 69.5, 69.4, 69.0, 68.9, 68.8, 66.9, 66.5, 63.1, 61.1, 60.89, 56.5, 50.0, 40.3, 38.9, 38.1, 35.5, 33.7, 27.8, 25.0 ppm. **HRMS**: calculated *m/z* for $C_{37}H_{67}N_4O_{18}S_2$ $(M+H)^+$ 919.3886; found 919.3899.

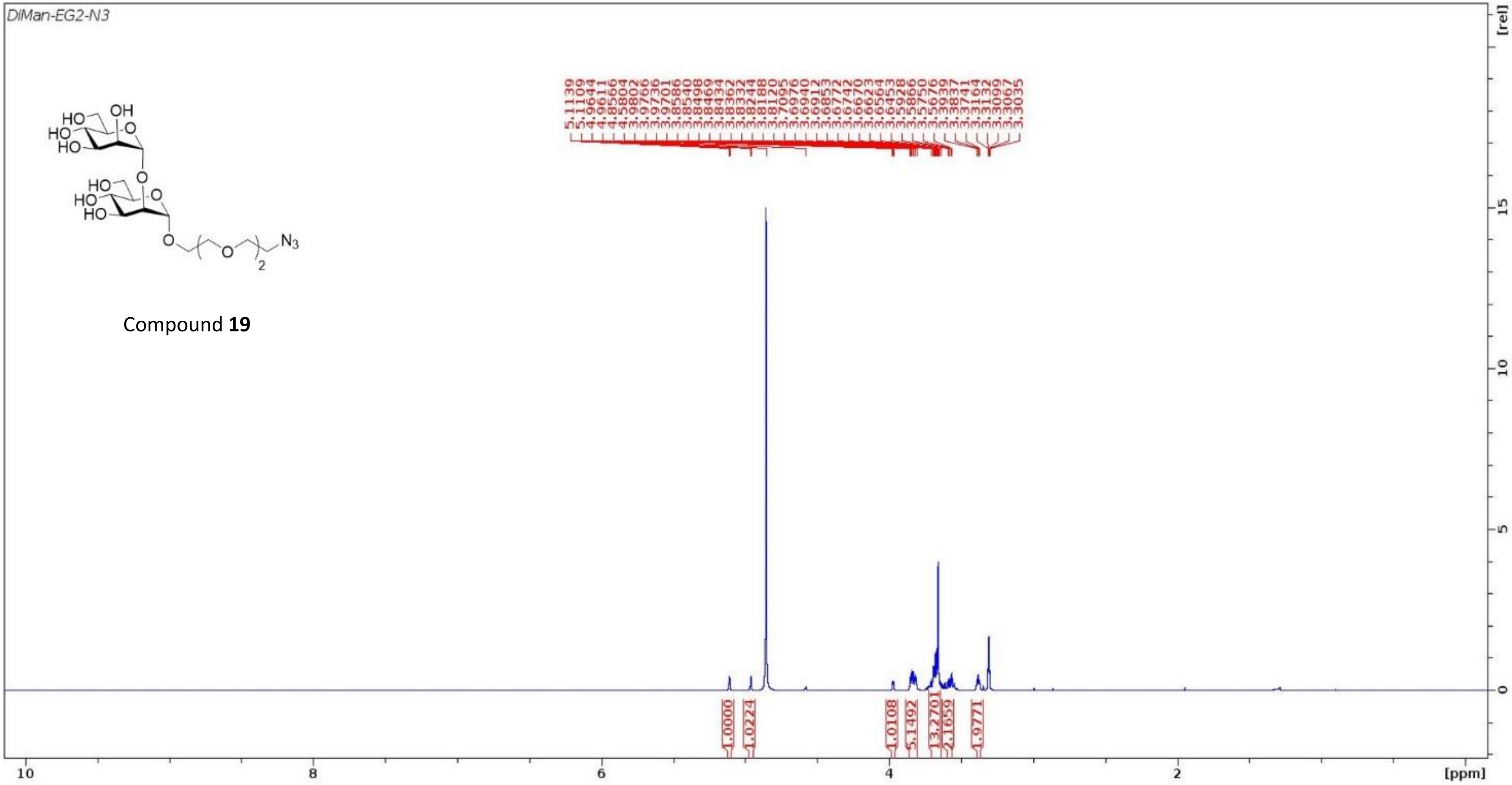

The ^{1}H NMR spectrum of compound **19** in D_2O

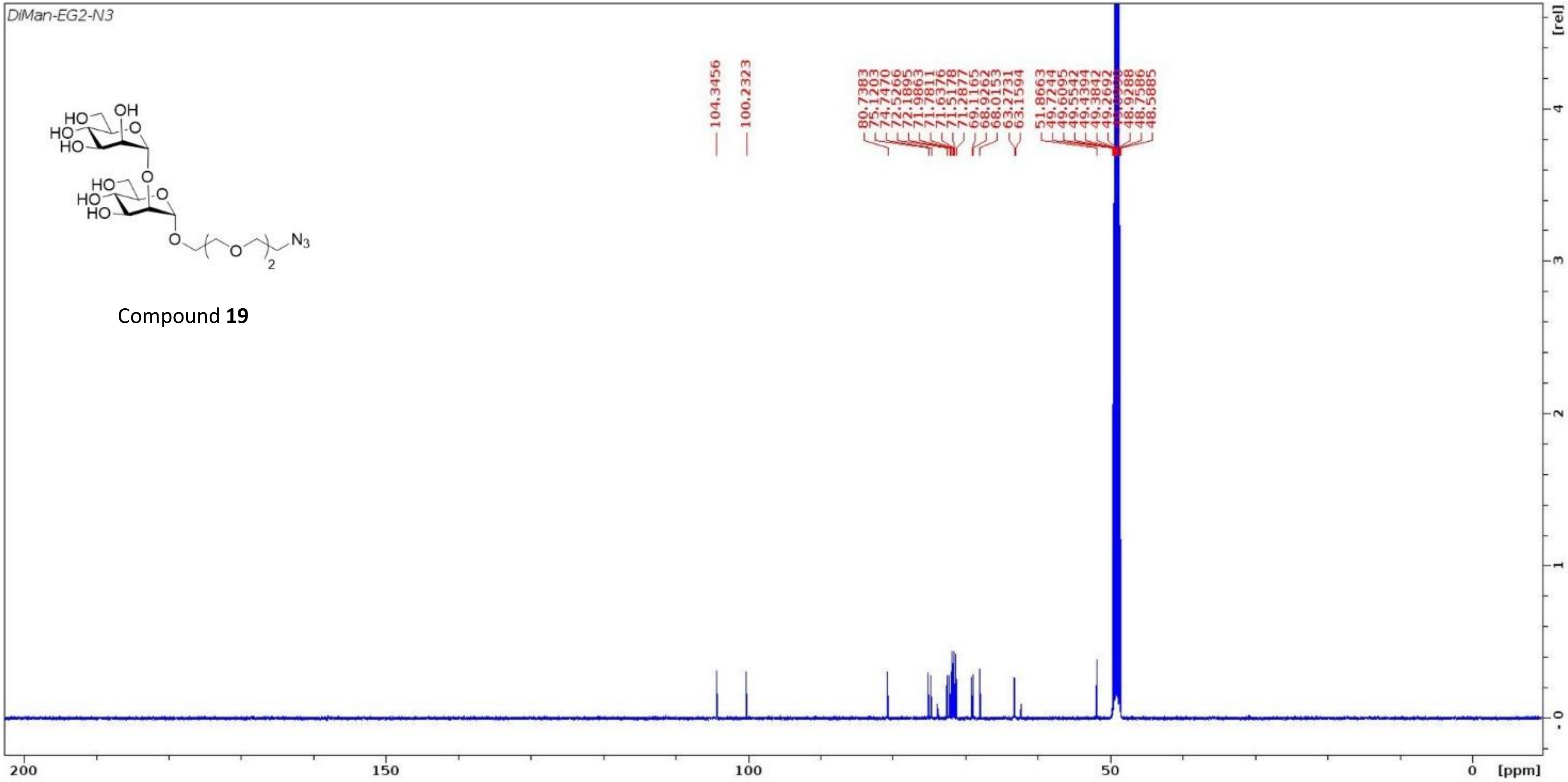

Compound **19**

The ^{13}C NMR spectrum of compound **19** in CD_3OD.

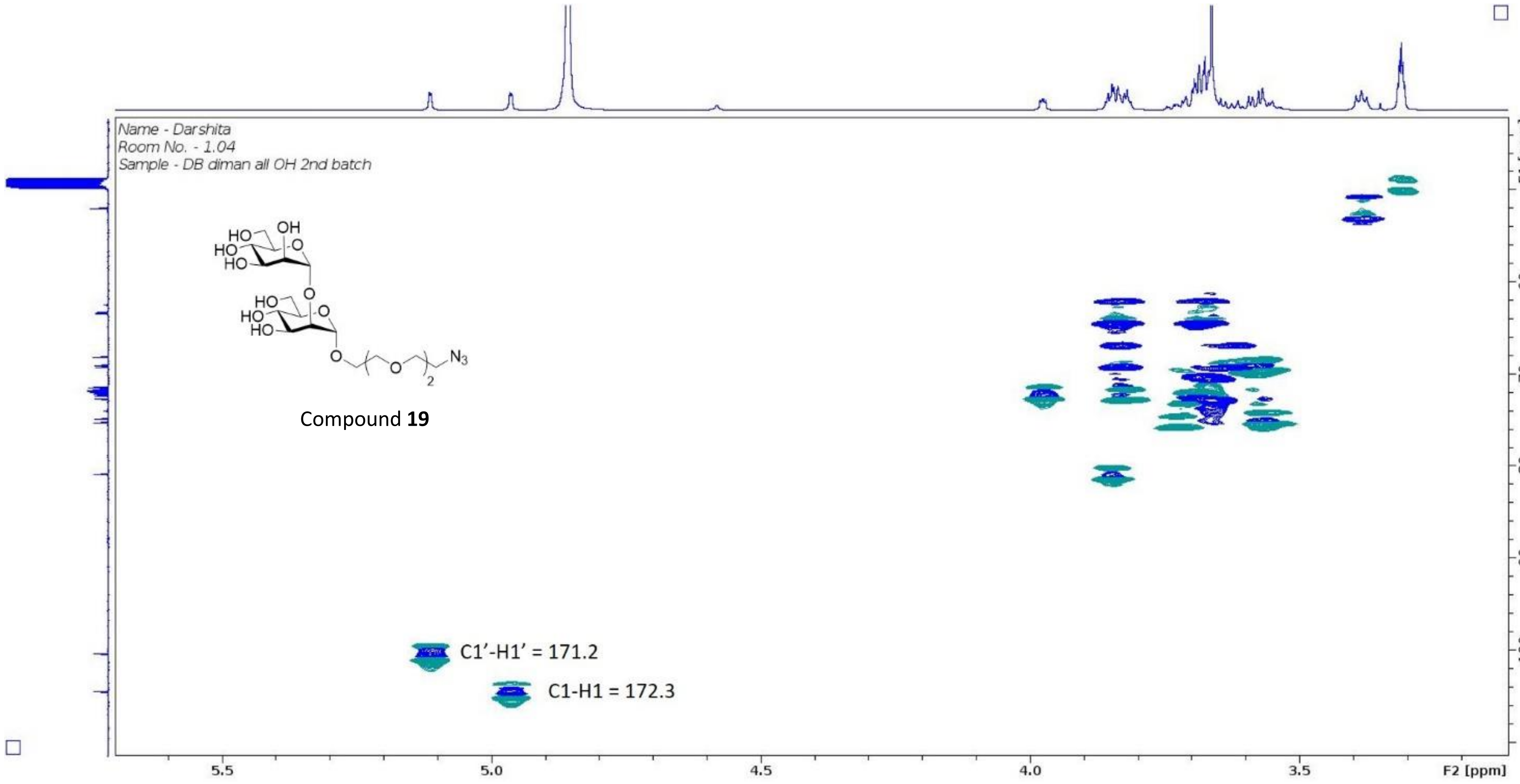

The coupled HSQC NMR spectrum of compound **19**.

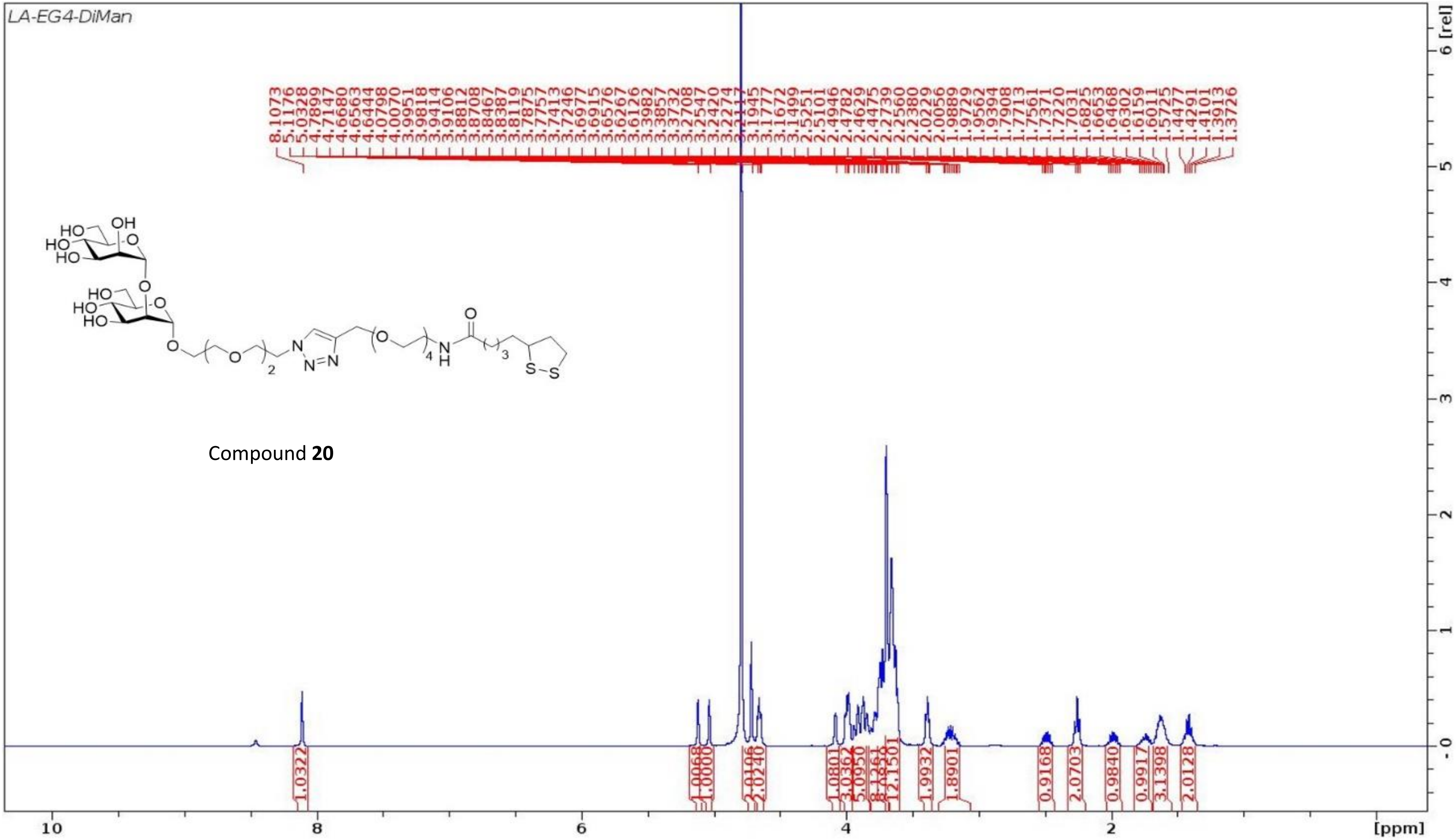

The ^{1}H NMR spectrum of compound **20** in D_2O.

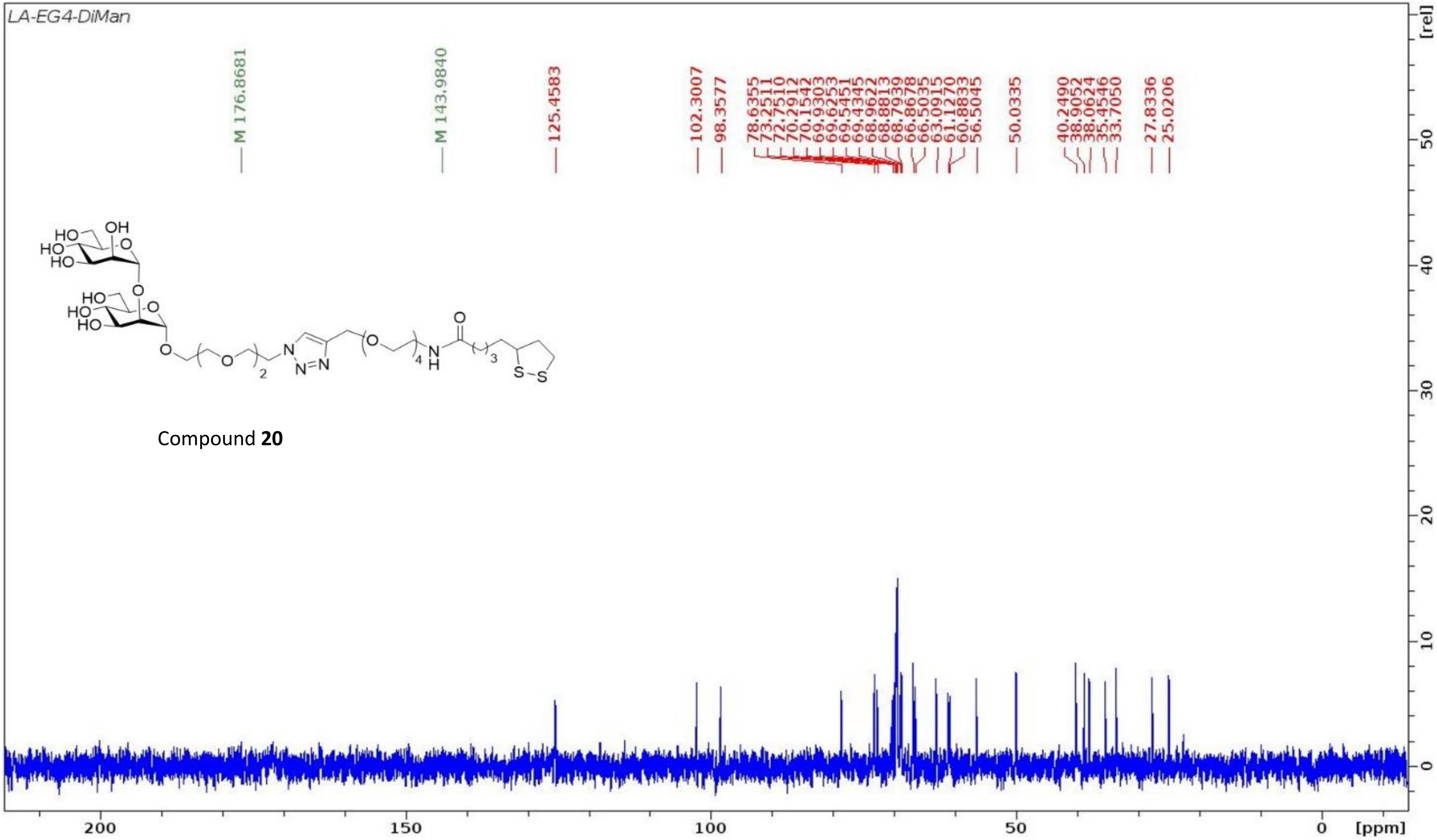

The ^{13}C NMR spectrum of compound **20** in D_2O.

General protocols for preparing DHLA-glycan ligands *via* TCEP reduction[5]

To the aqueous solution of LA-EG_4-glycans (20 mM, 200 µL, 4 µmol) in an Eppendorf tube, an aqueous solution of tris(2-carboxyethyl)phosphine hydrochloride (TCEP.HCl, 120 mM, 40 µL, 4.8 µmol) was added. The reaction mixture was shaken at room temperature for 1 h till LCMS indicated the complete reduction of the starting material. The solvent was evaporated *in vacuo* and the crude product was purified using a very small silica gel column and eluted with $CHCl_3$: CH_3OH = 2:1 for DHLA-EG_4-Gal and 1:1 for DHLA-EG_4-DiMan. The fractions containing the desired product was characterised by LCMS and combined and were used directly for the synthesis of glycan-capped QDs.

DHLA-EG_4-Gal (compound 21)

Yield = 80%. **LCMS**: calculated *m/z* for $C_{31}H_{59}N_4O_{13}S_2$ $(M+H)^+$ 759.35; found 759.30.

DHLA-EG_4-DiMan (compound 22)

Yield = 72%. **LCMS**: calculated *m/z* for $C_{37}H_{69}N_4O_{18}S_2$ $(M+H)^+$ 921.40; found 921.33.

4. ECD and CRD N-terminal labeling with LA-EG_{11}-TzTFP or LA-EG_{11}-TFP

DC-SIGN ECD or CRD N-terminal labeling was conducted in a low pH buffer (pH = 6.2) containing 20 mM HEPES, 150 mM NaCl, and 10 mM $CaCl_2$ (denoted as labeling buffer). The pKa of N-terminal amine is ~2-4 pH units lower than that of other amines on protein surface (*e.g.* lysine residues). Therefore, performing the labeling reaction at a low pH can ensure that only the N-terminal amine will be able to react with the TFP ester to form singly N-terminal labeled proteins, and not with other surface amines. [6] This is important to ensure that after conjugation to G13, the ECD or CRD will be oriented to have the glycan binding site facing outward and thus readily available for binding. Here LA-EG_{11}-TFP linker (in dry DMSO) was added to CRD in labeling buffer at a linker: CRD molar ratio of 1.5:1. The mixture was mixed on a rotating mixer at room temperature for 40 min and then diluted with the binding buffer (20 mM HEPES, 100 mM NaCl, 10 mM $CaCl_2$, pH 7.8). Any unbound free linkers were removed by washing with the binding using 10 kDa MWCO ultra-filtration membrane.

<u>Analysis ECD/CRD labeling efficiency with LA-EG_{11}-Tz-TFP by HRMS.</u> The calculated molecular weights of singly labeled LA-Tz-ECD and LA-Tz-CRD are 40512.89 and 19108.82, respectively and the observed MW are: 40517.99 and 19105.76, respectively. Species of molecular weight of 39202.46 and 17793.15 are unlabeled ECD and CRD. The peak area of each species were calculated and used to estimate the labeling efficiency, giving a single-labeling efficiency of 18% and 22% for ECD and CRD, respectively.

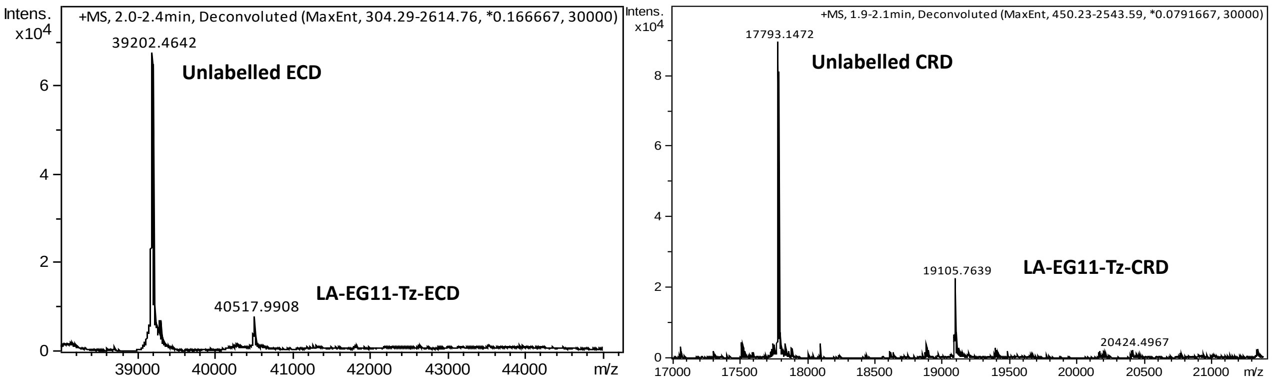

Figure S4.1. HRMS spectra showing the formation of single-linker labeled ECD (left) and CRD (right) using the LA-EG_{11}-Tz-TFP labelling.

Estimation of CRD labeling with LA-EG_{11}-TFP by HRMS:

The labeling efficiency was confirmed by HRMS, which gave three different specifies with MWs of 17793.22, 18608.63 and 19424.01 (see below), corresponding to the unlabeled CRD, single labeled-CRD (LA-EG_{11}-CRD), and double labeled-CRD ($(LA\text{-}EG_{11})_2$-CRD), respectively. The calculated MWs of each species are 17794.72, 18610.06, and 19424.40, respectively. The peak area of each species were calculated and used to estimate the labeling efficiency, giving an efficiency of 19% and 2% for the single- and double- labeled CRD, respectively. The total labeling efficiency was 21% under such conditions.

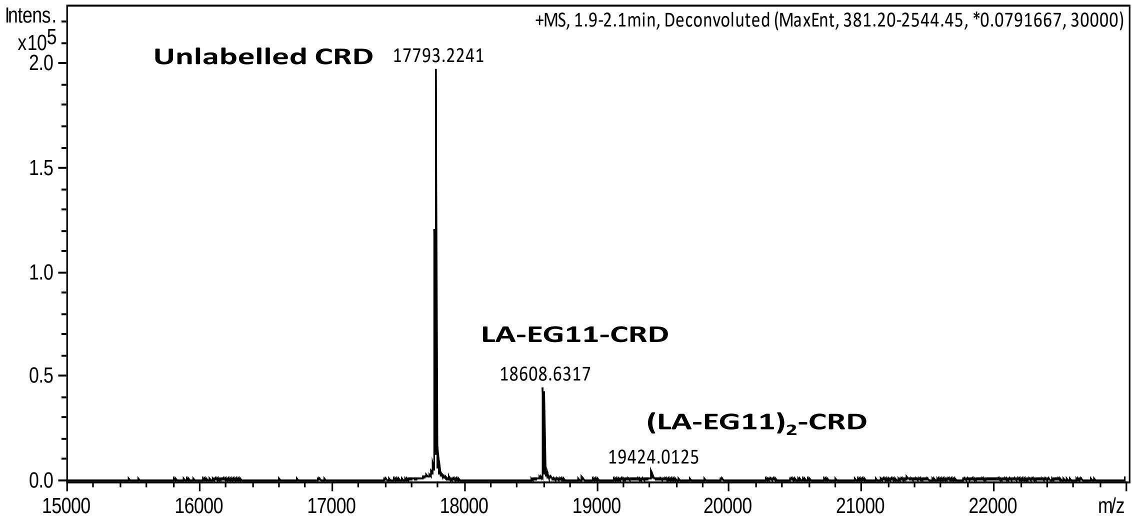

Figure S4.2. HRMS spectrum of CRD after labeling with LA-EG_{11}-TFP.

5. Preparation and modification of 13 nm diameter gold nanoparticle (G13)

5.1. Citrate stabilized G13 preparation. G13 was prepared using the standard citrate reduction of $HAuCl_4$ method *via* our established procedures. [7] The hydrodynamic diameter (D_h) of G13 was characterized by dynamic light scattering on a Malven Zetasizer-Nano using disposable polystyrene cuvette as before.[1,2] A total of 10 scans for each sample were recorded and the resulting size distributions were added up to produce the overall size distribution histogram (volume population). The histogram was then fitted by log-normal Gaussian function to determine the mean size and full-width at half-maximum (FWHM), giving a D_h of 15.1 ± 3.2 nm (mean ± ½ FWHM, see **Figure S5.3**). The concentration of G13 was determined by the Beer-Lambert Law using its peak absorbance at 519 nm and an extinction coefficient of 2.32×10^8 M^{-1} cm^{-1}. [7]

5.2 G13 PEGylation and G13-ECD, G13-CRD conjugation

5.2.1 G13 PEGylation

a) Estimation of ligand: G13 ratio required for G13 PEGylation.

- Surface area of a G13 particle: $A = \pi \times D^2 = 3.14 \times 13^2 = 531$ nm^2
- Assuming thiol occupies the 3 fold hollow site as that in self-assembled monolayers of thiolate molecules on a gold (111) surface, then each thiol molecule would occupy a surface area: $a = 0.214$ nm^2/molecule.
- Minimal thiol molecule: G13 molar ratio to fully cap the G13 surface: N = 531/0.214 = 2480.
- Therefore, a minimal thiol ligand: G13 molar ratio of 2480:1 is required to cap the G13 surface gold atoms fully (assuming all added thiol molecules are bound onto the G13 surface). To ensure the G13 surface was fully capped, a thiol ligand: G13 molar ratio of 5000 (~doubling the minimal ligand:G13 ratio) was used to prepare the fully PEGylated G13 negative control (G13-OH).

b) Preparation of partially PEGylated G13 (ppG13-OH).

To prevent G13 aggregation during protein conjugation which needs to be carried out in a binding buffer (20 mM HEPES, 100 mM NaCl, 10 mM $CaCl_2$, pH =7.8), the citrate stabilised G13 was first treated with a hepta(ethylene glycol) thiol (HS-EG_7-OH) to partially PEGylate the G13 surface. Briefly, citrate stabilised G13 was mixed with HS-EG_7-OH in water at a HS-EG_7-OH: G13 molar ratio of 2000:1 under stirring at RT for 48 h. The resulting mixture was then concentrated using 100 kDa MWCO filter tubes and washed with 100 mL water to remove any unbound free ligands. This yielded partially PEGylated G13 (denoted as ppG13-OH) that was found to be highly stable in binding buffer and is monodispersed.

c) Preparation of fully PEGylated G13 control (G13-OH).

Citrate stabilised G13 was incubated with HS-EG_7-OH at a thiol ligand:G13 molar ratio of 5000:1 (twice that required for full surface capping) for 48 h, and then it was washed using the same method as above to give the negative control, G13-OH.

5.2.2. Preparation of G13-ECD and G13-CRD.

The partially PEGylated ppG13-OH in water was first added 1/4 of its volume of a 5x binding buffer (100 mM HEPES, 750 mM NaCl, 50 mM $CaCl_2$, pH 7.8) to make it in a final 1x binding buffer, and then linker labeled ECD or CRD was added. For LA-EG_{11}-Tz-ECD, LA-EG_{11}-Tz-CRD and LA-EG_{11}-CRD, the molar ratio of protein: ppG13-OH of 100:1 were used. To further investigate how CRD:G13 ratio affect its conjugation efficiency and viral inhibition, the molar ratio of LA-EG_{11}-CRD: ppG13-OH of 115:1 was used. The resulting mixed solutions were stirred at 4 ºC overnight (~16 h). After that, the conjugation mixture was transferred to an ultrafiltration tube equipped with a 100 kDa MWCO membrane and was centrifuged to collect conjugates (which cannot pass through the membrane). The flow through filtrate was collected and characterised by HRMS. As shown in resulting HRMS below, only unlabeled ECD or CRD, was detected in the filtrate, suggesting that all of linker labeled ECD or CRD have bound to G13. Therefore, G13-Tz-ECD, G13-Tz-CRD and G13-CRD prepared at a labeled protein:G13 ratio of 100 and 115 would give a CRD valency of ~100 and ~115 per G13, abbreviated as G13-Tz-ECD_{100}, G13-Tz-$CRD_{100,}$ G13-CRD_{100} and G13-CRD_{115}, respectively. The resulting GNP-lectin conjugates were further washed three times with the binding buffer using the same 100 kDa MWCO ultrafiltration tube, before being transferred to sample vials. G13-lectin conjugates concentration were determined from its UV-vis absorbance at 520 nm using G13's extinction efficient (2.32 x 10^8 M^{-1}.cm^{-1}, ECD and CRD show no absorption at this wavelength).

LA-EG11-Tz-ECD before mixing with ppG13-OH

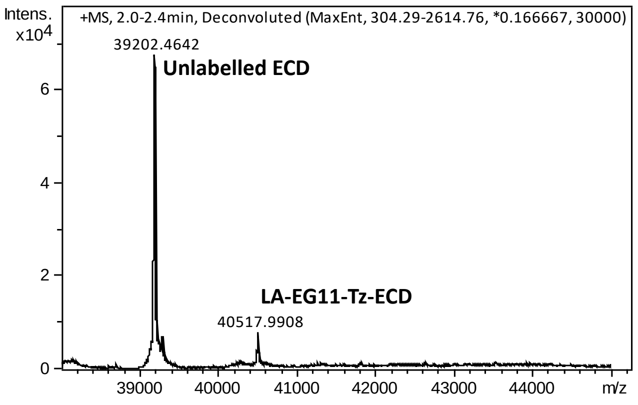

Filtrate separated from G13-Tz-ECD

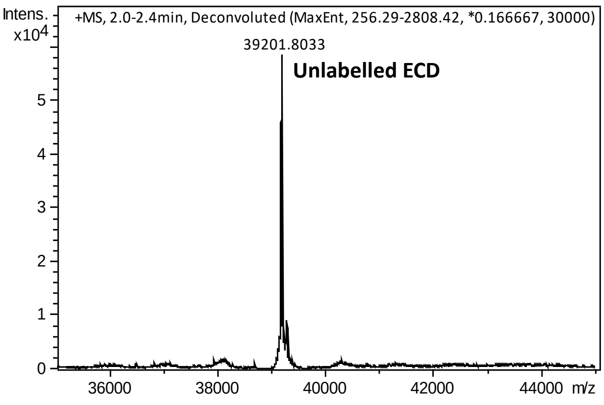

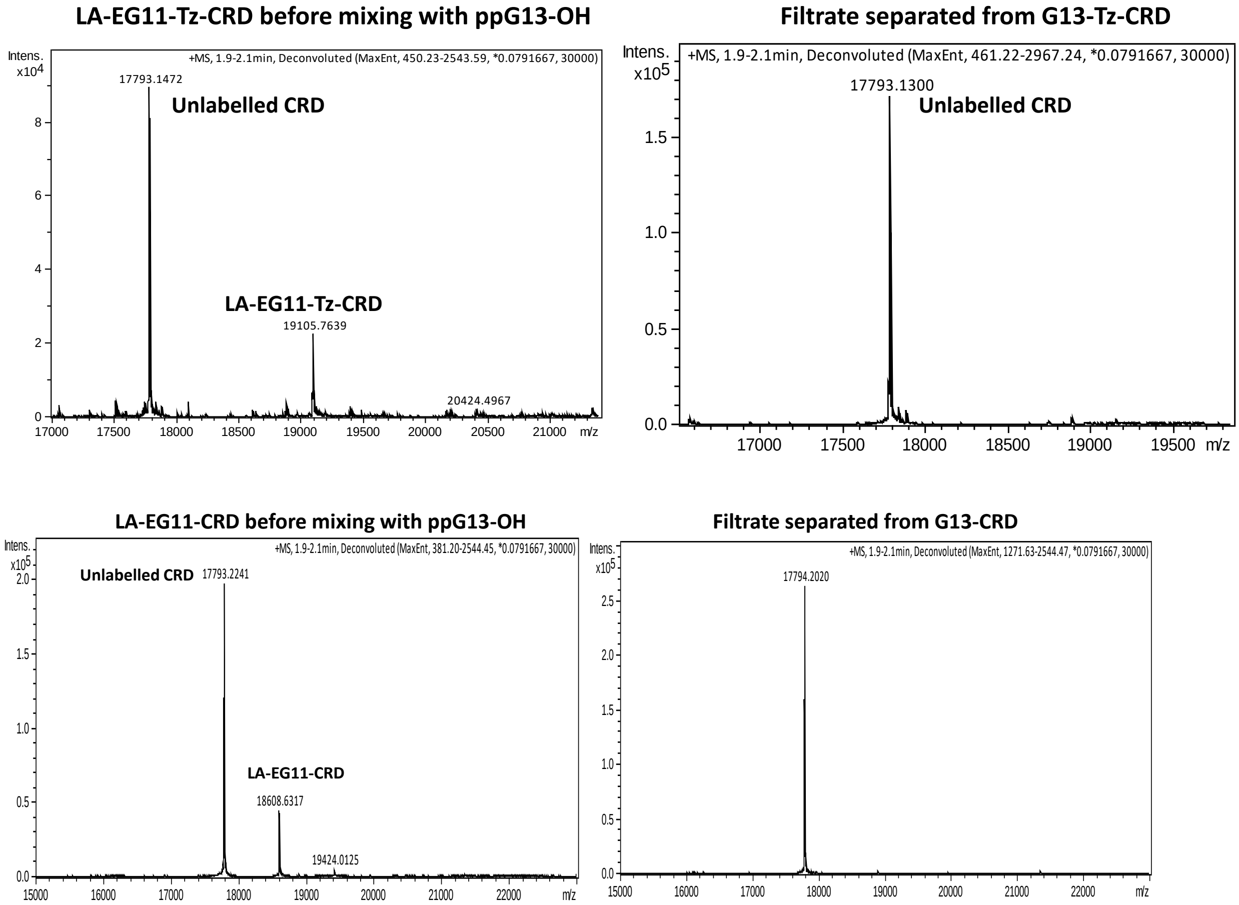

Figure S5.1. HRMS study of linker labeled protein before mixing with ppG13-OH and filtrate after conjugation. All linker labeled proteins have completely disappeared in the filtrate, leaving only the unlabeled proteins, suggesting that all linked labeled protein have conjugated to G13.

5.3 GNP-CRD characterization

5.3.1. Conjugates sizes and state of dispersion by agarose gel and DLS:

The successful preparation of G13 controls and G13-CRD were confirmed by gel electrophoresis. G13-$(EG_7\text{-}OH)_{2000}$ before and after conjugation with CRD as well as the control G13$(EG7\text{-}OH)_{5000}$ were analysed by 1.5% agarose gel in TAE buffer. The samples were mixed with 60% glycerol and then loaded onto the gel and run at 100m V for 40 min. As shown in the gel image below, G13-CRD exhibited reduced gel mobility compared to partially PEGylated ppG13-OH, most likely due to an increase of size after CRD conjugation. While G13-OH control showed the lowest gel mobility, likely due to greatly reduced charge density after full surface PEGylation.

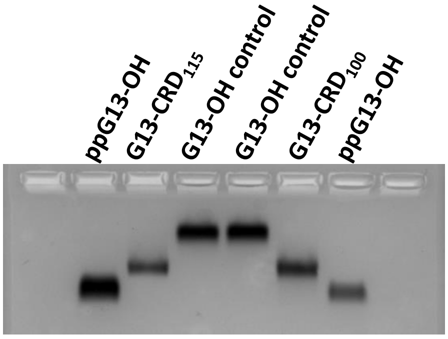

Figure S5.2. Agarose gel electrophoresis showing the different gel mobility of various G13 conjugates. ppG13-OH has the highest gel mobility, followed by G13-CRD, and the fully PEGylated G13-OH control has the lowest mobility.

5.3.2. Hydrodynamic size measurement

The hydrodynamic diameters (D_hs) of ppG13-OH, and G13-OH control (in water) and G13-TZ-ECD_{100}, G13-Tz-CRD_{100}, G13-CRD_{100} and G13-CRD_{115} in a binding buffer (20 mM HEPES, 100 mM NaCl, 10 mM $CaCl_2$, pH = 7.8) were measured on a Malven Zetasizer-Nano as described above. Their D_h histograms were fitted by Gauss distribution to estimate their mean size as below.

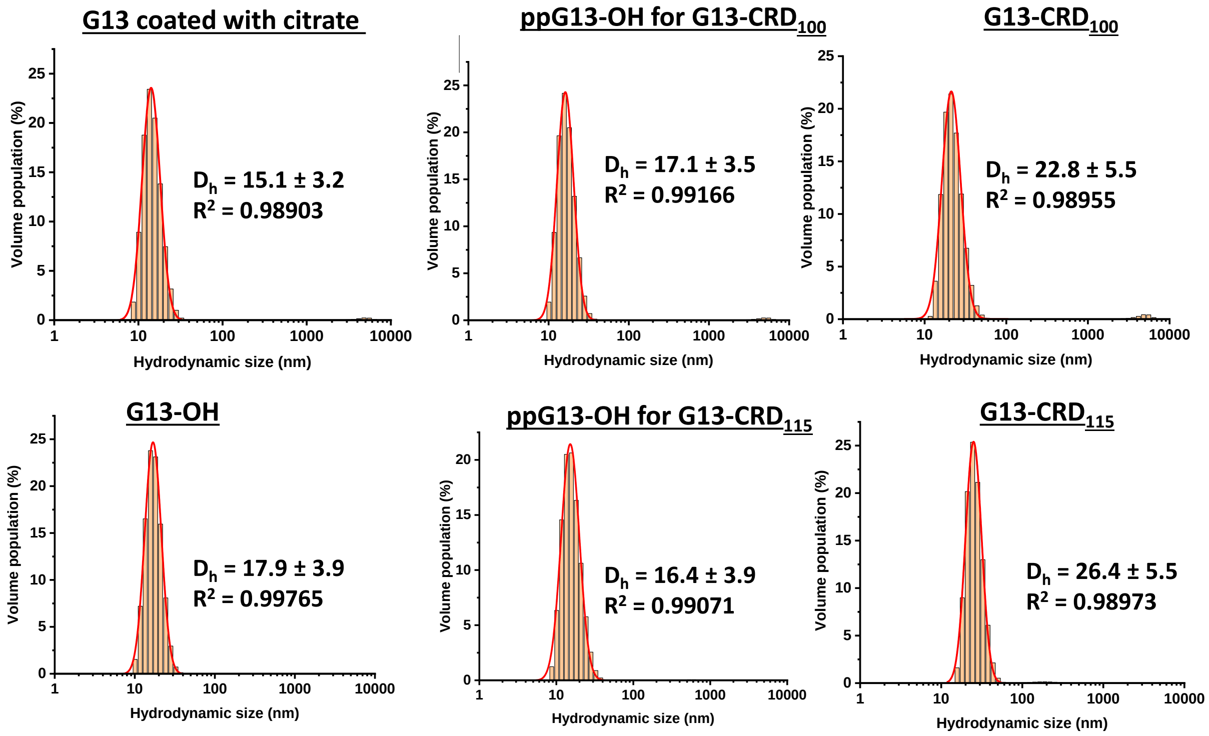

Figure S5.3. D_h size histograms of various G13 conjugates measured by DLS. The data were fitted by log-normal Gaussian function, and the D_h sizes were given as mean ± ½ FWHM (full width at half maximum) of the fit.

5.3.3. G13-CRD binding specificity and affinity with mannose coated QDs *via* fluorescence quenching

LA-EG_4-Man-α1,2-Man or LA-EG_4-galactose are reduced to DHLA-EG_4-Man-α1,2-Man or DHLA-EG_4-galactose by TCEP and then conjugated to a CdSe/ZnS core/shell with peak emission at ~605 nm (QD_{605}) to make QD-DiMan or QD-Gal as a positive or a negative control, respectively as described previously. [2] The QD concentration was determined by measuring its absorbance at the first exciton peak at 589 nm using an extinction coefficient of 3.3×10^5 $M^{-1}\cdot cm^{-1}$. [2]

QD-DiMan or QD-Gal (final concentration = 2 nM) was mixed with G13-CRD or G13-OH control (final concentration = 1 nM) in an Eppendorf tube in a binding buffer (20 mM HEPES, 100 mM NaCl, 10 mM $CaCl_2$, pH7.8) containing 1 mg/mL BSA to minimise any nonspecific interactions and absorption of the QD and GNP on surfaces. The mixture was incubated at room temperature for 20 min before the fluorescence spectra were recorded (λ_{EX} = 400 nm, emission range: 570-700 nm) using a Cary Eclipse Fluorescence Spectro-photometer.

To quantify binding affinity, different concentrations of QD-DiMan was mixed with G13-CRD under a fixed QD: G13 molar ratio of 1:1 in the binding buffer containing 1 mg/ml BSA. The samples were incubated at room temperature for 20 min before fluorescence spectra were recorded (λ_{EX} = 400 nm, emission range: 570-700 nm). Adjustments of the PMT voltages and EX/EM slit widths were used to compensate the low

fluorescence signal at low concentrations, ensuring all fluorescence intensities were in the linear response range and below instrument saturation levels.

To analyse the binding data quantitatively, quenching efficiency (QE) at each concentration (C) was calculated against the QD only control. The apparent binding dissociation constant (K_d) was derived from the QE-C relationship by fitting the data using Hill's equation as described before. [3] Briefly, quenching efficiency was calculated as:

$$QE\% = \frac{(IF_0 - IF)}{IF_0} \times 100\%$$

Where IF_0 and IF are the integrated fluorescence of the QD-DiMan in the absence and presence of each G13 -CRD, respectively. The apparent equilibrium dissociation constant (K_d) can be derived by fitting with Hill's equation:

$$QE\% = \frac{QE_{max} \times C^n}{K_d n + C^n}$$

Where QE_{max}, K_d, C, and n are the maximum QE (fixed at 100), apparent binding dissociation constant, QD-DiMan concentration, and Hill coefficient, respectively.

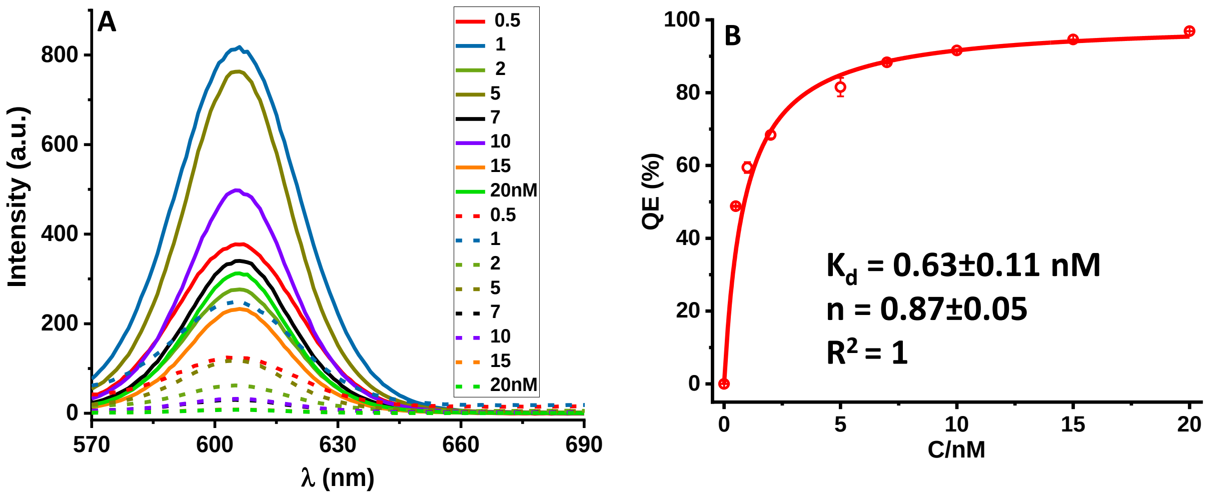

Figure S5.4. (**A**) Fluorescence spectra varying concentrations of QD-DiMan in the absence (solid line) or presence (dash line) of 1:1 mixed G13-CRD_{100}. (**B**) The relationship between QD-DiMan quenching efficiency (QE) and G13-CRD100 concentration fitted by the Hill's equation, giving an apparent binding equilibrium dissociation constant, K_d, of 0.63 ± 0.11 nM.

6. Pseudo-SARS-CoV-2 virus preparation and inhibition studies.

6.1. Pseudo-SARS-CoV-2 virus preparation

Vesicular stomatitis virus (VSV) particles pseudotyped with SARS-CoV-2 S protein and encoding a luciferase gene were generated as described previously.[8] We and others have shown previously that these particles adequately model SARS-CoV-2 entry into cells and its inhibition.[9-11] All cell culture was performed in Dulbecco's modified Eagle medium (DMEM) (PAN-Biotech, Aidenbach, Germany), supplemented with 10% fetal bovine serum (FBS) (Biochrom Berlin, Germany) and penicillin (100 U/mL)/streptomycin (0.1 mg/mL) solution (P/S) (PAA Laboratories GmbH, Cölbe, Germany) as reported previously. [9]

6.2. Inhibition of Pseudo-SARS-CoV-2 infection

To evaluate G13-CRD's inhibitory effect on SARS-CoV-2 S protein-driven cell entry, Vero76 cells were seeded in 96-well plates at a density of $2x10^5$ cells per well. Equal volumes of pseudotype preparations and G13-CRD were incubated in DMEM containing 10% FBS at 37°C for 2 h. Medium was aspirated from the cells (at 24 h post seeding), then pseudotype viral particles and G13-CRD mixture (100 μL) was added to each well and cells were incubated at 37°C for 16-18h. After that, the cell medium was removed and cells were lysed using PBS supplemented with 0.5% Triton X-100 (Carl Roth, Germany) for 30 min at RT. Then 30 μL of cell lysates were transferred into white 96-well plates, mixed with luciferase substrate (Beetle- Juice, PJK) and then luminescence signals were measured with a Hidex Sense Plate luminometer (Hidex). The luciferase activities in cell lysates from each treatment were normalized against the corresponding control measured in the absence of G13-CRD. The normalized infection (*NI*)-*C* relationship was fitted by the modified inhibition model to derive the apparent viral inhibition potencies (EC_{50} and n values) as described in the main text.

The same protocol was used to evaluate how DC-SIGN-binding glycan molecules (mannose, glucose and mannan) may compete with pseudotypes bearing SARS-CoV-2 (B.1 variant) S-protein for binding to G13-CRD_{115} (labeled as G13-CRD1 in **Fig. S6.5 A**), thereby reducing G13-CRD_{115} ability to inhibit viral transduction. Each glycan competitor (various doses) was pre-incubated with G13-CRD_{115} (50 nM final dose) at 37 ^{o}C for 1 h before being added to B.1 pseudotype particles and further incubated for 2 h at 37 ^{o}C. Finally, the B.1 pseudotype/glycan/G13-CRD1 mixture was added to Vero76 cells to evaluate their antiviral properties using the same steps as above.

6.3 Statistical analysis

Microsoft Excel (as part of the Microsoft Office software package, version 2019, Microsoft Corporation) and GraphPad Prism 9 version 9.0.2 (GraphPad Software) were used to analyze the data. Statistical analysis was carried out by a Brown-Forsythe and Welch ANOVA analysis with Dunnett's T3 multiple comparison test. Only p-values of 0.05 or less were considered to be statistically significant (NS (not significant) $p > 0.05$; * $p \leq 0.05$; ** $p \leq 0.01$; *** $p \leq 0.001$; **** $p \leq 0.0001$).

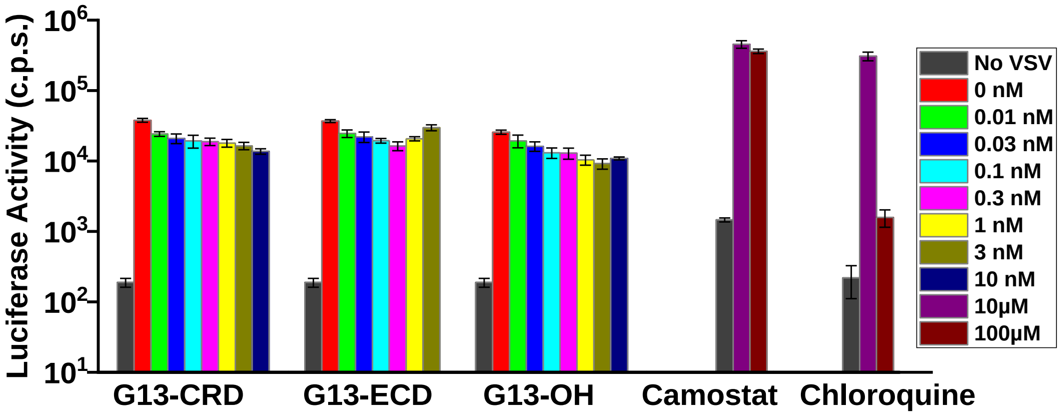

Figure S6.1 Inhibition of pseudotype particles bearing the vesicular stomatitis virus glycoprotein (VSV-G, as a negative control) by various G13-conjugates (G13-Tz-CRD_{100}, G13-Tz-ECD_{100}, and G13-OH) together with Camostat or chloroquine. No significant inhibition was observed for all G13-conjugates as expected. Camostat showed no apparent inhibition even at a high dose of 100 μM, while chloroquine showed significant inhibition at 100 μM as expected.

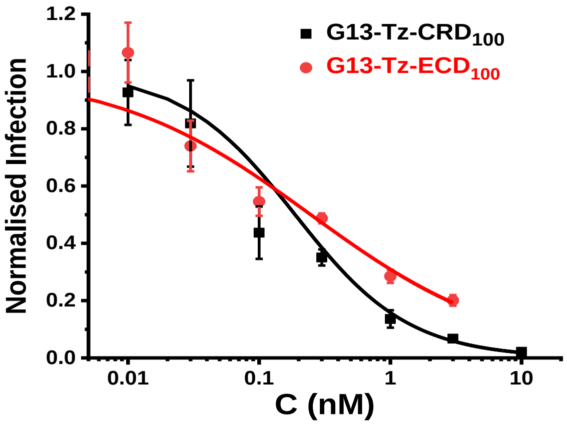

Figure S6.2. Normalised dose-dependent inhibition of VSV particles pseudotyped with SARS-CoV-2 (Hu-1) S protein entry Vero76 cells by G13-Tz-CRD_{100} (black) and G13-Tz-ECD_{100}. The data were fitted by a modified inhibition model, $NI = 1/[1 + (C/EC_{50})^n]$, yielding EC_{50} = 0.25±0.04 nM, n = 0.57 ± 0.06, R^2 = 0.970 for G13-Tz-ECD_{100} and EC_{50} = 0.19 ± 0.02 nM, n = 1; R^2 = 0.974 for G13-Tz-CRD_{100}, respectively.

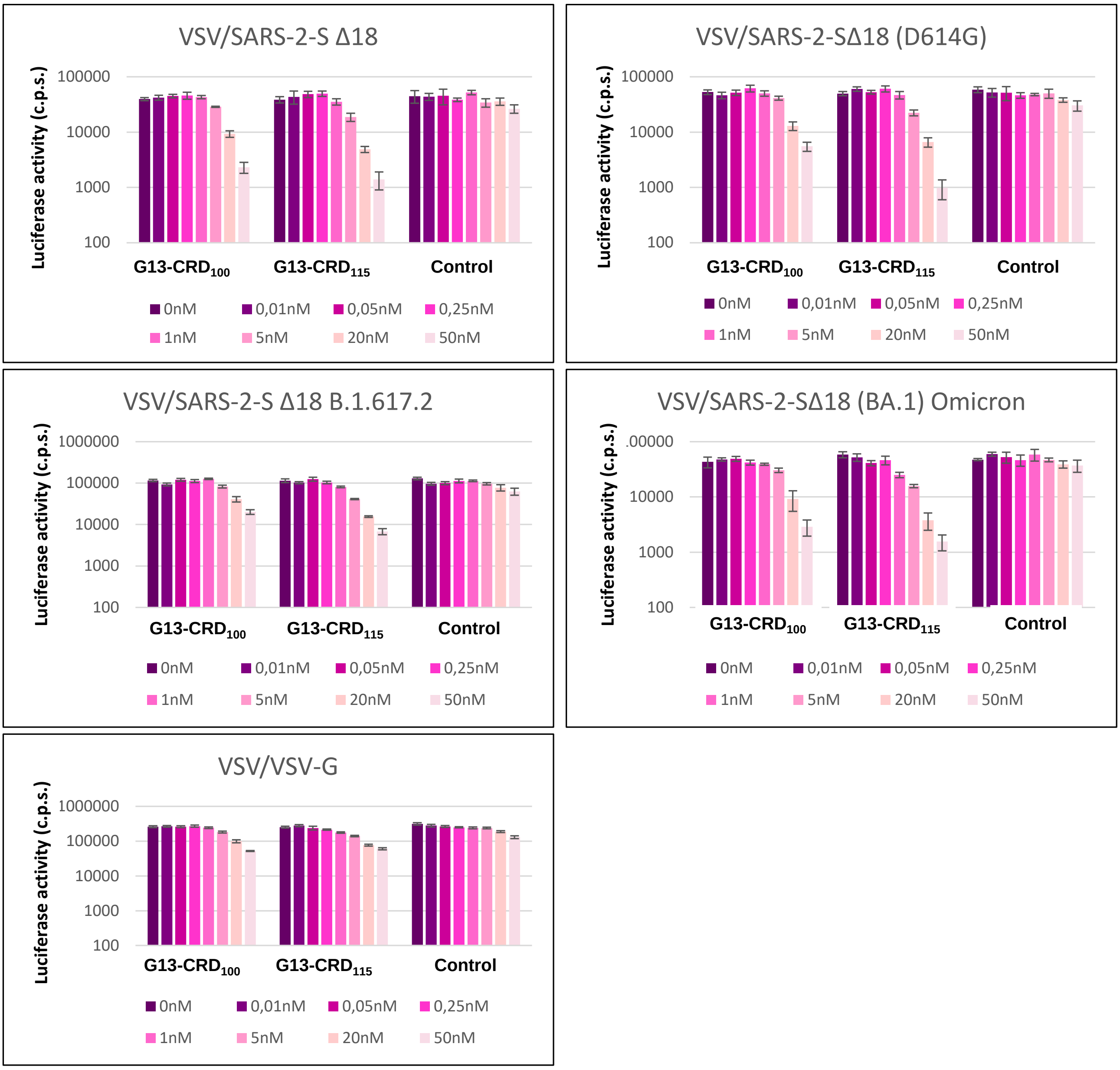

Figure S6.3. Summary of dose-dependent inhibition by G13-CRD$_{100}$, G13-CRD$_{115}$ and G13-OH control against VSV particles pseudotyped with the following SARS CoV-2 S-proteins, WT (Wuhan wild-type, **A**); B.1 (**B**); B.1.617.2 (Delta, **C**) and BA.1 (Omicron, **D**) or a control vesicular stomatitis virus glycoprotein (VSV-G) (**E**) infection of Vero 76 cells. G13-CRD$_{115}$ gives the highest inhibition while G13-OH control shows no significant inhibition.

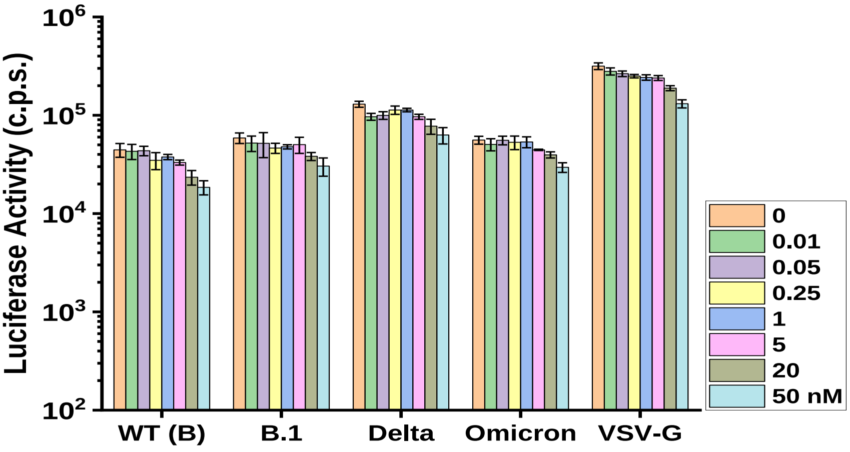

Figure S6.4. The G13-OH control does not significantly inhibit entry driven by the S proteins of the indicated SARS-CoV-2 variants or VSV-G. VSV particles bearing the indicated SARS-CoV-2 S proteins (Wuhan Hu-1 (WT), B.1, Delta (B.1.617.2), Omicron (BA.1)) were pre-incubated with G13-CRD$_{115}$ (**A**) at the indicated concentrations in DMEM containing 10% FBS, and then added to Vero76 cells. Entry into Vero76 cells was determined by quantifying luciferase activity in cell lysates. The results of a single representative experiment performed with technical quadruplicates are shown here and were confirmed in two separate experiments. Error bars indicate standard errors.

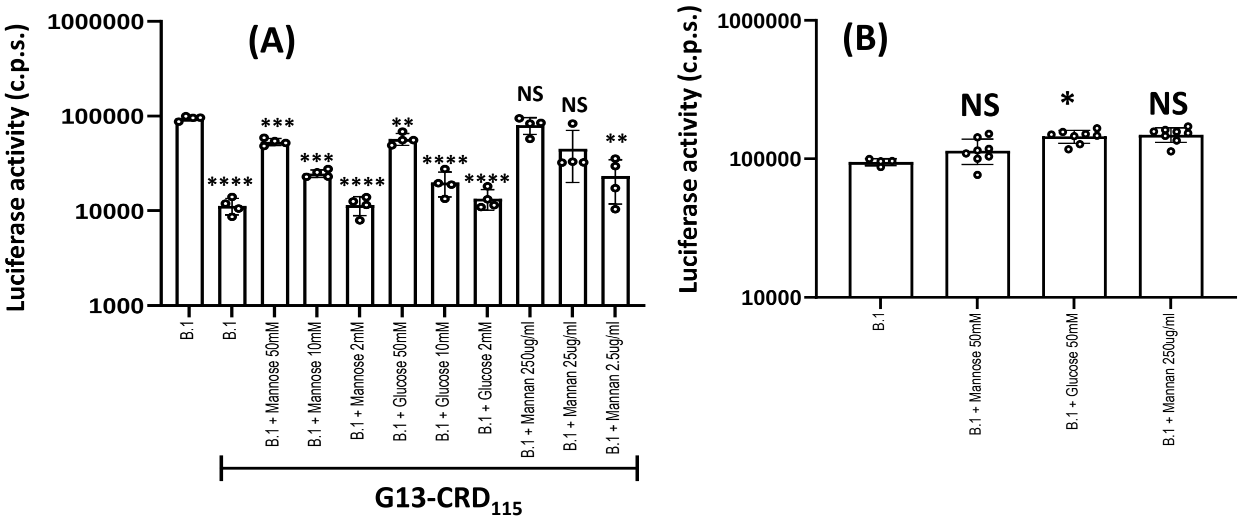

Figure S6.5. (**A**) Glycan competitors (mannose, glucose and mannan) dose-dependently reduce G13-CRD$_{115}$ (50 nM)-mediated inhibition of Vero76 cell entry of VSV particles pseudotyped with the SARS-CoV-2 B.1 S protein. Compared with untreated particles (B.1, left column), G13-CRD$_{115}$ (50 nM) significantly reduced B.1 S protein-mediated transduction (by ~90%, 2nd left column). The presence of mannose, glucose and mannan (all can bind to DC-SIGN to compete with its binding with other glycan targets) significantly and dose-dependently restored B.1 S protein-mediated transduction, demonstrating that the anti-SARS-CoV-2 properties of G13-CRD$_{115}$ originate from specific glycan binding. (**B**) Treatment of VSV particles with glycan competitor molecules alone (at the highest concentrations used in panel **A**) does not modulate B.1 S protein-driven entry into Vero76 cells.

Statistical differences between B.1 alone and individual groups with different treatments were assessed by a Brown-Forsythe and Welch ANOVA analysis with Dunnett's T3 multiple comparison test as reported previously. NS (not significant) $p > 0.05$; $^*p \leq 0.05$; $^{**}p \leq 0.01$; $^{***}p \leq 0.001$; $^{****}p \leq 0.0001$.

7. Inhibition of authentic SARS-CoV-2 infection by G13-CRD_{115}

All work with infectious SARS-CoV-2 was conducted under BSL-3 conditions at the German Primate Centre, Göttingen, Germany. Vero76 cells were seeded in 96-well plates at a density of 2 x 10^5 cells per well. Different doses (ranging from 0.1 to 50 nM) of G13-CRD_{100}, G13-OH, or Sotrovimab (kindly provided by Sebastian Schulz and Hans-Martin Jäck from Friedrich-Alexander University of Erlangen-Nürnberg, Germany) were each incubated with SARS-CoV-2 isolate NK, Pango lineage B.1.513 (kindly provided by Stephan Ludwig, Institute of Virology, University of Münster, Münster, Germany) or SARS-CoV-2 isolate Omicron BA.1, Pango lineage BA.1 (kindly provided by Christian Drosten, Institute of Virology, Charité-Universitätsmedizin Berlin, Germany) at 37 ^{o}C for 2 h in an inoculation volume of 100 µL. Afterwards, Vero76 cells were infected with the virus-inhibitor mixtures at an MOI of 0.01 at 37 ^{o}C. After one hour incubation, the inoculum was removed, cell cultures were washed with PBS two times, and 100 µL of culture medium was added to the cells. Supernatants were collected at 0 and 48 h post infection (hpi) and stored at -80 °C until further usage. Viral titres were determined by plaque assay on Vero76 cells as described previously,[9,10] and are given as PFU/mL.

8. Estimation of inter-spike distance on virion surface.

The Cryo-EM image of the authentic early pandemic wildtype SARS-CoV-2 (B.1) revealed that the virion particle is roughly spherical of ~95 nm in diameter (D = 95 nm) at the foot of S-proteins. Each SARS-CoV-2 virion particle is randomly decorated with ~40 S-proteins (N = 40).[13]

Using these structural data, the inter-S-protein distance can be estimated using the literature method as follows.[2,14]

The footprint of each S-protein on the virion surface is given as,

$$k = 4\pi r^2/N \qquad (1)$$

Where r is the radius of the virion particle (r = D/2).

The average deflection angle (θ, in degrees) of each S-protein on virion particle surface can be estimated as:

$$\theta = 360^o/(k\pi)^{1/2} = 229.3^o/(N)^{1/2} = 229.3^o/(40)^{1/2} = 36.3^o \qquad (2)$$

The average inter-S-protein distance (X) can be calculated as:

$$X = 2\,r \sin(\theta/2) = 95 \sin(18.15^o) = \sim 30 \text{ nm}$$

References

(1). Guo, Y.; Nehlmeier, I.; Poole, E.; Sakonsinsiri, C.; Hondow, N.; Brown, A. et al., Dissecting Multivalent Lectin–Carbohydrate Recognition Using Polyvalent Multifunctional Glycan-Quantum Dots. *J. Am. Chem. Soc*. **2017**, *139*, 10833-10844.

(2) Budhadev, D.; Poole, E.; Nehlmeier, I.; Liu, Y. Y.; Hooper, J.; Kalverda, E.; Akshath, U. S.; Hondow, N.; Turnbull, W. B.; Pohlmann, S.; Guo, Y.; Zhou, D. J. Glycan-Gold Nanoparticles as Multifunctional Probes for Multivalent Lectin-Carbohydrate Binding: Implications for Blocking Virus Infection and Nanoparticle Assembly. *J. Am. Chem. Soc.* **2020,** *142*, 18022-18034.

(3) Abellán Flos; M., García Moreno, M. I.; Ortiz Mellet, C., Garcia Fernandez, J. M., Nierengarten, J. F., Vincent, S. P. Potent glycosidase inhibition with heterovalent fullerenes: Unveiling the binding modes triggering multivalent inhibition. *Chem. Eur. J.* **2016**, *22,* 11450-11460.

(4) Ribeiro-Viana, R.; Sánchez-Navarro, M.; Luczkowiak, J.; Koeppe, J. R.; Delgado, R.; Rojo, J.; *et al.* Virus-like Glycodendrinanoparticles Displaying Quasi-equivalent Nested Polyvalency Upon Glycoprotein Platforms Potently Block Viral Infection. *Nat. Commun.* **2012**, *3,* 1-9.

(5) Burns, J. A.; Butler, J. C.; Moran, J.; Whitesides, G. M. Selective Reduction of Disulfides by Tris (2-carboxyethyl) Phosphine. *J. Org. Chem.* **1991**, *56,* 2648-2650.

(6) Rosen, C. B.; Francis, M. B., Targeting the N terminus for Site-Selective Protein Modification. *Nat. Chem. Biol.* **2017,** *13*, 697-705.

(7) Song, L.; Ho, V. H. B.; Chen, C.; Yang, Z. Q.; Liu, D. S.; Chen, R. J.; Zhou, D. J., Efficient, pH-Triggered Drug Delivery Using a pH-Responsive DNA-Conjugated Gold Nanoparticle. *Adv. Healthcare Mater.* **2013,** *2*, 275-280.

(8) Chaudhry, M. Z.; Eschke, K.; Hoffmann, M.; Grashoff, M.; Abassi, L.; Kim, Y.; Brunotte, L.; Ludwig, S.; Kröger, A.; Klawonn, F.; Pöhlmann, S. H.; Cicin-Sain, L. Rapid SARS-CoV-2 Adaptation to Available Cellular Proteases *J. Virol.,* 2022, 96, e02186-21.

(9) Hoffmann, M.; Kleine-Weber, H.; Schroeder, S.; Kruger, N.; Herrler, T.; Erichsen, S.; Schiergens, T. S.; Herrler, G.; Wu, N. H.; Nitsche, A.; Muller, M. A.; Drosten, C.; Pohlmann, S., SARS-CoV-2 Cell Entry Depends on ACE2 and TMPRSS2 and Is Blocked by a Clinically Proven Protease Inhibitor. *Cell* **2020,** *181*, 271-280.

(10) Hoffmann, M.; Krüger, N.; Schulz, S.; Cossmann, A.; Rocha, C.; Kempf, A.; Nehlmeier, I.; Graichen, L.; Moldenhauer, A. S.; Winkler, M. S.; Lier, M.; Dopfer-Jablonka, A.; Jäck, H.-M.; Behrens, G. M. N.; Pöhlmann, S., The Omicron Variant Is Highly Resistant Against Antibody-Mediated Neutralization: Implications for Control of the COVID-19 Pandemic. *Cell* **2022,** *185,* 447-456.

(11) Schmidt, F.; Weisblum, Y.; Muecksch, F.; Hoffmann, H. H.; Michailidis, E.; Lorenzi, J. C. C.; Mendoza, P.; Rutkowska, M.; Bednarski, E.; Gaebler, C.; Agudelo, M.; Cho, A.; Wang, Z.; Gazumyan, A.; Cipolla, M.; Caskey, M.; Robbiani, D. F.; Nussenzweig, M. C.; Rice, C. M.; Hatziioannou, T.; Bieniasz, P. D., Measuring SARS-CoV-2 Neutralizing Antibody Activity Using Pseudotyped and Chimeric Viruses. *J. Exp. Med.* **2020,** *217*, e20201181.

(12) Mitchell, D. A.; Fadden, A. J.; Drickamer, K. A Novel Mechanism of Carbohydrate Recognition by the C-type Lectins DC-SIGN and DC-SIGNR. *J. Biol. Chem*. **2001**, *276*, 28939-28945.

(13) Ke, Z.; Oton, J.; Qu, K.; Cortese, M.; Zila, V.; McKeane, L.; Nakane, T., *et al.* Structures and Distributions of SARS-CoV-2 Spike Proteins on Intact Virions *Nature* **2020**, *588,* 498-502.

(14) Hill, H. D.; Millstone, J. E.; Banholzer, M. J.; Mirkin, C. A., The Role Radius of Curvature Plays in Thiolated Oligonucleotide Loading on Gold Nanoparticles. *ACS Nano* **2009,** *3*, 418-424.
